# Structure-activity relationship study of mesyl and busyl phosphoramidate antisense oligonucleotides for unaided and PSMA-mediated uptake into prostate cancer cells

**DOI:** 10.3389/fchem.2024.1342178

**Published:** 2024-03-04

**Authors:** O. Sergeeva, E. Akhmetova, S. Dukova, E. Beloglazkina, A. Uspenskaya, A. Machulkin, D. Stetsenko, T. Zatsepin

**Affiliations:** ^1^ Skolkovo Institute of Science and Technology, Moscow, Russia; ^2^ Department of Chemistry, Lomonosov Moscow State University, Moscow, Russia; ^3^ Department for Biochemistry, People’s Friendship University of Russia Named after Patrice Lumumba (RUDN University), Moscow, Russia; ^4^ Institute of Cytology and Genetics, Siberian Branch of the Russian Academy of Sciences, Novosibirsk, Russia

**Keywords:** ASO, alkanesulfonyl phosphoramidate, PSMA, prostate cancer, MALAT1 lncRNA

## Abstract

Phosphorothioate (PS) group is a key component of a majority of FDA approved oligonucleotide drugs that increase stability to nucleases whilst maintaining interactions with many proteins, including RNase H in the case of antisense oligonucleotides (ASOs). At the same time, uniform PS modification increases nonspecific protein binding that can trigger toxicity and pro-inflammatory effects, so discovery and characterization of alternative phosphate mimics for RNA therapeutics is an actual task. Here we evaluated the effects of the introduction of several *N*-alkane sulfonyl phosphoramidate groups such as mesyl (methanesulfonyl) or busyl (1-butanesulfonyl) phosphoramidates into gapmer ASOs on the efficiency and pattern of RNase H cleavage, cellular uptake *in vitro*, and intracellular localization. Using Malat1 lncRNA as a target, we have identified patterns of mesyl or busyl modifications in the ASOs for optimal knockdown *in vitro*. Combination of the PSMA ligand-mediated delivery with optimized mesyl and busyl ASOs resulted in the efficient target depletion in the prostate cancer cells. Our study demonstrated that other *N*-alkanesulfonyl phosphoramidate groups apart from a known mesyl phosphoramidate can serve as an essential component of mixed backbone gapmer ASOs to reduce drawbacks of uniformly PS-modified gapmers, and deserve further investigation in RNA therapeutics.

## 1 Introduction

Antisense oligonucleotides (ASOs) are key players among RNA therapeutics that can target undruggable so far proteins by downregulating their gene expression (Migawa et al., 2019; Shen et al., 2019; Crooke et al., 2021a). The field of ASO has evolved a lot since the first experimental proof 45 years ago, when an ASO showed activity *in vivo* (Zamecnik and Stephenson, 1978). It took about a decade to establish the first “antisense” pharmaceutical companies, which immediately faced numerous problems during preclinical and clinical studies, including poor stability, toxicity and immunostimulatory activity of ASO. Obviously, ASO can benefit from longer duration of their action, enhanced endosomal escape, decreased toxicity and immunogenicity as well as from fewer off-target effects. Most of these risks can be mitigated by targeted delivery and chemical modification (Gagliardi and Ashizawa, 2021). Massive efforts over the last 30 years have led to the technology success with more than 10 clinically approved ASO drugs (Crooke et al., 2021b), including Eteplirsen for Duchenne Muscular Dystrophy (2016), Nusinersen for spinal muscular atrophy (SMA) (2016), Volanesorsen for Familial Chylomicronemia Syndrome or Hypertriglyceridemia (2019) and others (Crooke et al., 2021a). All the approved ASOs are used to treat rare diseases, and they also provide an excellent opportunity to develop personalized drugs such as Milasen (Kim et al., 2019) and splice-switching ASOs for ataxia-telangiectasia treatment (Kim et al., 2023). This amazing opportunity to create a drug for a single patient was never achieved by any other modality previously. Nusinersen has changed the life of patients with SMA, and is the most commercially successful RNA drug with >2 billion annual revenue (Finkel et al., 2017). Thus, RNA therapeutics are recognized as an emerging drug modality that should revolutionize the field of genetic medicine, and the way of treatment for many other diseases.

ASO can rely on different molecular mechanisms of action such as RNase H-dependent mRNA and lncRNA degradation, correction of pre-mRNA splicing, inhibition of mRNA translation, blocking of pre-microRNA maturation or microRNA-mRNA interactions, and modulation of nonsense-mediated mRNA decay (Crooke et al., 2021b; Sergeeva et al., 2022). Since the initial attempts of ASO development, chemical modification of the phosphate backbone, ribose moiety, and nucleobases were recognized as one of the main approaches that allow the *in vivo* application of ASOs, and affect the mechanism of their action (Bennett and Swayze, 2010). Unmodified DNA oligonucleotides are quickly degraded by nucleases, so the first generation of ASOs incorporated phosphorothioate modification (PS) to greatly improve stability. PS ASOs supported RNase H-mediated mRNA digestion, showed reliable biodistribution and pharmacokinetics but demonstrated structure-related toxicity (Crooke et al., 2020). To improve the RNA binding and nuclease stability, 2ʹ-*O*-methyl (2′-OMe) and 2ʹ-*O*-methoxyethyl (2′-MOE) ribonucleotides were introduced into ASOs, but the fully 2′-modified oligonucleotides were unable to recruit RNase H anymore (Crooke et al., 2021c). The latter found application in splice switching, while the combination of phosphorothioate modification, internal DNA gap and 2ʹ-modifications in the outer wings helped to develop a second generation gapmer ASOs with the increased potency, stability and reduced pro-inflammatory effects (Monia et al., 1997). 2′-Modified nucleotides in the wings additionally prevented nucleolytic degradation of the ASO by exonucleases, while several DNA nucleotides in the middle permitted RNase H recruitment (Monia et al., 1993; Crooke et al., 1995; Wu et al., 1999). Apart from phosphorothioate backbone and 2ʹ-*O*-alkyl ribose moieties, 2ʹ-deoxy-2ʹ-fluoro-D-arabino nucleic acids (FANA) and locked or constrained nucleic acids (LNA and cEt) were developed for further improvement of nucleolytic and RNA duplex stability as well as pharmacokinetic/pharmacodynamic characteristics of ASOs (Damha et al., 1998; Kurreck et al., 2003; Pellestor and Paulasova, 2004; Hagedorn et al., 2018). A majority of chemistry efforts were directed at sugar moieties and nucleobases due to wide opportunities to build diverse structures, while phosphate modification was somewhat more conservative. Among the approved drugs only two phosphate-modified ASOs are present: phosphorothioates (PS) and phosphorodiamidate morpholino oligomers (PMOs) (Wan and Seth, 2016). One more molecule with modified phosphate groups, namely, N3′→P5′ thiophosphoramidate oligonucleotide Imetelstat may be approved this year after successful Phase III of clinical trials as a treatment for patients with transfusion-dependent anemia in lower-risk myelodysplastic syndromes (Chiappori et al., 2015). The PS linkages are also beneficial for gapmer ASOs due to enhanced interactions with many plasma, cell surface and intracellular proteins (Crooke et al., 2021b), thus facilitating uptake of ASOs into cells (Liang et al., 2015; Wang et al., 2016; Miller et al., 2016). However, multiple adverse effects for modified ASOs are still common (Henry et al., 1997; Jason et al., 2004). Non-specific protein binding of PS ASOs is the major driver for pro-inflammatory effects, species-specific complement activation, and thrombocytopenia (Crooke et al., 2021a). PMO is a very distinct type of oligonucleotides widely used for splice switching during aberrant splicing events. PMO are uncharged at neutral pH, and demonstrate poor interactions with proteins and low toxicity. However, these benefits lead to poor pharmacokinetic/pharmacodynamic properties. There are also examples of boranophosphates, carboxymethyl C-phosphonates, phosphoryl guanidines and other phosphate modifications that show some promising results in early discovery (Kupryushkin et al., 2014; Clavé et al., 2020). So, new phosphate mimics in ASO that can reduce toxicity and improve efficacy are needed. Recently, it was shown that *N*-mesyl (methanesulfonyl) phosphoramidate (MsPA, or μ) (Miroshnichenko et al., 2019; Patutina et al., 2020; Anderson et al., 2021; Chelobanov et al., 2017) or *N*-(4-acetamidobenzenesulfonyl) phosphoramidate (Kandasamy et al., 2022) groups can be efficient replacements to phosphorothioate (PS) groups in ASOs. Introduction of mesyl phosphoramidate modifications into ASOs led to higher targeted specificity, reduced toxicity and improved activity over the PS analogues for targeting miRNAs (Miroshnichenko et al., 2019). Not so far ago, another alkanesulfonyl phosphoramidate group, namely, 1-butanesulfonyl (busyl) phosphoramidate (β) has been described (Burakova et al., 2019).

In spite of significant progress in RNA therapeutics, their targeted delivery to cells remains a major challenge that limits clinical applications (Dhuri et al., 2020; Gagliardi and Ashizawa, 2021). There are few clinically approved solutions such as delivery of naked ASO to the liver, kidney and CNS, and *N*-acetylgalactosamine (GalNAc) conjugates to the liver. For example, GalNAc conjugation improves ASO delivery into hepatocytes by approximately 30-fold due to receptor-mediated endocytosis via asialoglycoprotein receptor (Prakash et al., 2014). Alternative advanced approaches for targeted ASO delivery include antibody (Malecova et al., 2023) and FAB (Desjardins et al., 2022) conjugates for delivery to the muscles, heart, and GLP-1 peptide for β-cells in the pancreas (Tsoumpra et al., 2019; Knerr et al., 2021; Gurlo et al., 2023). These examples clearly demonstrate prospects for targeted delivery of ASO conjugates with ligands of internalizable cellular receptors.

Prostate cancer (PСa) is the most frequent male cancer worldwide, with high mortality and morbidity rates. PCa pathogenesis is characterized by high levels of androgen receptor (AR). The primary way to treat the disease is androgen deprivation therapy (ADT). However, many tumors progress into the castration-resistant form (CRPC) after ADT, as the AR signaling pathway can be restored due to mutations, alternative splicing, and increased intra-tumoral hormone levels (Heinlein and Chang, 2004). ASOs demonstrated the efficiency in silencing the AR gene and suppressing prostate tumor growth in castration-naive and castration-resistant murine models (De Velasco et al., 2019). PS ASO IONIS-AR-2.5Rx (AZD5312) targeting the androgen receptor mRNA demonstrated limited results in patients with metastatic castration-resistant prostate cancer (mCRPC) due to PK/PD limitations. The same ASO IONIS-AR-2.5Rx is under further investigation in combination with the anti-androgen drug enzalutamide in mCRPC (De Velasco et al., 2019). Consequently, androgen-independent cells express high amounts of prostate-specific membrane antigen (PSMA) on their surface (Schülke et al., 2003). In several clinical studies PSMA ligands showed good results for the delivery of diagnostic and therapeutic agents (Wu et al., 2011; Barve et al., 2014; Wang et al., 2021). Also, radioisotope labeled PSMA ligands were applied for a diagnostical PSMA-based PET imaging, and in 2022 FDA approved a radiopharmaceutical ^177^Lu-PSMA-617 to treat PCa (Hoshi et al., 2023).

**FIGURE 1:**
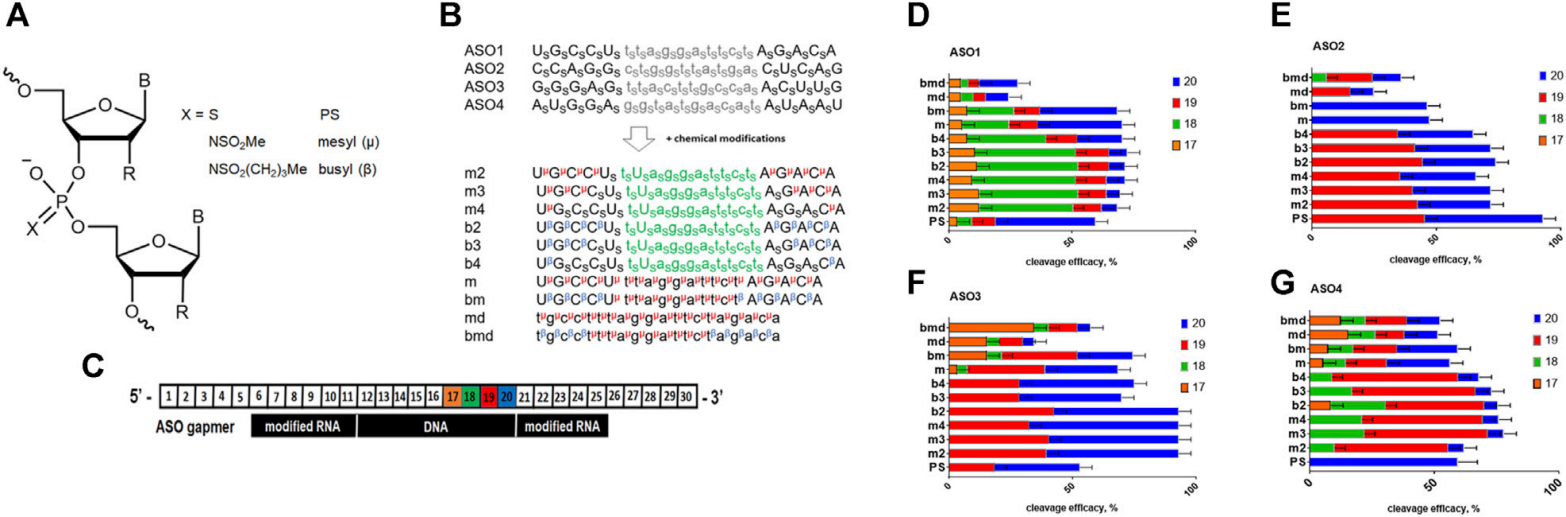
**(A)** Structure of the ASO modifications used in this study; R = H or OMe. **(B)** Scheme of the modified ASO structure. _S_–phosphorothioate; ^μ^–mesyl phosphoramidate; ^β^–busyl phosphoramidate; A, G, C, and U: 2′-OMe RNA nucleotides; a, g, c, and t: deoxynucleotides. **(C)**. Scheme of the duplex between Malat1 RNA and modified ASO with RNA nucleotide positions numbered. **(D–G)** RNase H cleavage patterns of ASO1-4: RNA heteroduplexes.

In this study, we combined the PSMA ligand-mediated delivery of ASOs with *N*-alkanesulfonyl phosphoramidate groups, mesyl and busyl (Figure 1A) to achieve efficient knockdown of Malat1 lncRNA in prostate cancer cells *in vitro*. We studied the effects of ASO modifications on the efficiency of RNase H cleavage, cellular uptake, and intracellular localization using Malat1 lncRNA as a target. After ASO optimization, we evaluated targeted delivery to 22Rv1 PSMA-positive prostate cancer cells using two previously published PSMA ligands–a common Glu-Lys urea-based peptidomimetic MA-415, and an advanced version with improved binding to the receptor MA-257 (Supplementary Figure S2) (Machulkin et al., 2019; Machulkin et al., 2021). Mesyl and busyl ASOs conjugated to a PSMA ligand demonstrated receptor-mediated delivery *in vitro* in the case of the improved PSMA binder.

## 2 Results

### 2.1 Design and synthesis of mesyl and busyl phosphoramidate 2′-OMe ASO gapmers

LncRNA metastasis-associated lung adenocarcinoma transcript 1 (Malat1) is overexpressed and act as an oncogene in most of malignant tumors such as breast cancer, lung cancer, and hepatocellular carcinoma. Previous studies also claimed that increased Malat1 expression was positively correlated with poor prognosis in pancreatic cancer and PCa (Ren et al., 2013; Chang et al., 2018a; Goyal et al., 2021). Malat1 expression is substantially higher in CRPC than in primary prostate cancer, and knocking it down resulted in decreased cell proliferation, invasion and migration, as well as in an increase in apoptosis (Ren et al., 2013). Moreover, positive correlation between Malat1 RNA level in PCa cells and the resistance to enzalutamide suggests the involvement of Malat1 in the production of androgen receptor splicing variant 7 (AR-v7) − the predominant AR splicing variant in enzalutamide-resistant PCa (Wang et al., 2017). Also, the sponge effect of Malat1 on miR-1, miR-320b and miR-1-3p affects PCa progression (Chang et al., 2018a; Dai et al., 2019). These features make Malat1 a perspective target for ASO-mediated therapy of PCa (Chang et al., 2018b). Therefore, we decided to focus on Malat1 as a target in this study.

We used four previously validated ASO sequences targeting Malat1 (Gutschner et al., 2013; Dong et al., 2017; Liang et al., 2017; Ämmälä et al., 2018), and designed a number of oligonucleotides with different patterns and combinations of chemical modifications (Supplementary Table S1). ASOs are usually more tolerant to various chemical modifications in comparison to other RNA therapeutics like siRNA or sgRNA. However, there are numerous examples when modified ASO changed selectivity or mechanism of action in comparison to parental sequences (Sergeeva et al., 2022). Previously, it was shown that duplexes of fully modified mesyl phosphoramidate oligonucleotides with RNA are good substrates of RNAse H (Miroshnichenko et al., 2019). Since we are interested in a deeper understanding of the influence on the level mRNA knockdown for mesyl and a more hydrophobic busyl phosphoramidate modifications, we decided to perform a more detailed study with the accurate positioning of modified residues within the ASO. Mesyl and busyl phosphoramidates were inserted into ASOs at different positions (Figure 1B), namely: a) mesyl phosphoramidate gapmers (m); b) gapmers with mesyl or busyl phosphoramidate groups in the wings, except for the two PS flanking the DNA gap (m2, b2); c) same as in b), but with +1×PS (m3, b3); d) same as in b), but with +2×PS (m4, b4); e) gapmers with busyl phosphoramidate groups in the wings and mesyl phosphoramidate groups in the gap (bm); f) all-DNA mesyl ASO (md); and g) all-DNA ASO with busyl phosphoramidate groups in the wings with mesyl phosphoramidates in the middle section (bmd). Since phosphate modifications exert a strong influence on oligonucleotide-protein interactions, we wanted to study the effects of mesyl and busyl phosphoramidate groups both in the first generation ASO and gapmer contexts. Synthesis of all-deoxy and 2′-*O*-methyl RNA/deoxy gapmer oligonucleotides with mesyl (μ) and busyl (β) phosphoramidate groups was accomplished according to previously published method of automated solid-phase assembly via phosphoramidite coupling followed by Staudinger reaction with 0.5 M solution of either methanesulfonyl azide or 1-butanesulfonyl azide in acetonitrile instead of iodine oxidation [61]. We have previously detected some enrichment in one of the two diastereomers (ca. 60% vs. 40%) for a single mesyl or busyl modifications on HPLC (Stetsenko, 2023) (Supplementary Table S2). However, with the rise in the number of modifications it becomes increasingly more difficult to attribute the peaks to individual diastereomers by ^31^P NMR. Some data on stereoregular mesyl oligonucleotides are avalable from Anderson *et al.* where no significant improvement over stereorandom oligomers were noted (Anderson et al., 2021). Such a set of stereorandom ASOs as was obtained was expected to be suitable for finding positions tolerant to mesyl or busyl phosphoramidate modifications both in deoxy and gapmer contexts. As even one modified residue can significantly change the geometry and interactive surface of the oligonucleotide and its duplex with RNA, we believe that the classical “ladder” approach, when a single modification “slides” along the sequence would have been less informative in this case.

### 2.2 Modulation of RNase H cleavage pattern by ASOs modified with mesyl and busyl phosphoramidate groups

Firstly, we studied the effects of mesyl and busyl phosphoramidate modifications on the RNase H cleavage patterns in ASO:RNA heteroduplexes. We used Cy5-labeled short fragments of Malat1 RNA, which contained sites for ASO hybridization (Supplementary Table S2). All modified ASO were hybridized with the appropriate RNA template followed by incubation with bacterial RNase H (Figure 1C). Catalytic domain of RNAse H from *Escherichia coli* is similar to the one of the human enzyme (Kielpinski et al., 2017), and demonstrates similar selectivity. We adjusted the conditions for RNase H cleavage to obtain around 50% efficacy of the RNA scission for ASO1 all-PS gapmer. All-PS ASO2 was more active, so its cleavage efficacy was *ca*. 90% under the same conditions, while in the case of all-PS ASO3 and ASO4 it was close to that for ASO1 (Figure 1E). We demonstrated that in the case of all-PS modified ASOs the main cleavage by RNase H occurs after the 20th nucleotide, with some cut after the 19th nucleotide in the RNA target. At the same time, for ASO1 we observed a minor cleavage at positions 17 and 18. Addition of mesyl and busyl phosphoramidate modifications to ASOs drastically changed the pattern of cleavage with the prevalence of the cut at the 19th position. For ASO4 and ASO1, significant cleavage after the 18th position was observed (m2-m4, b2-b4). If mesyl and busyl phosphoramidate modifications were introduced into the DNA gap (bmd, md, bm, m) of ASO1, ASO3, ASO4, additional sites of cleavage appeared (Figures 1D–G). Thus, we can conclude that the presence of mesyl or busyl phosphoramidate modifications within the gap region of the ASOs significantly changed the cleavage pattern. Thus, one might expect some changes in the RNase H cleavage pattern for the m type vs. b type gapmers. Fully mesylated oligodeoxynucleotides are nearly as good as the unmodified DNA in supporting RNase H activity (Miroshnichenko et al., 2019), whereas fully busylated oligodeoxynucleotides are rather poor substrates for the enzyme (Stetsenko and Sugimoto, 2023). At the same time, the influence of three to four mesyl and busyl groups in the 2′-modified wings of a gapmer in comparison to all-PS ASOs was much less pronounced.

### 2.3 Functional activity of mesyl or busyl phosphoramidate modified ASOs in prostate cancer cells

**FIGURE 2:**
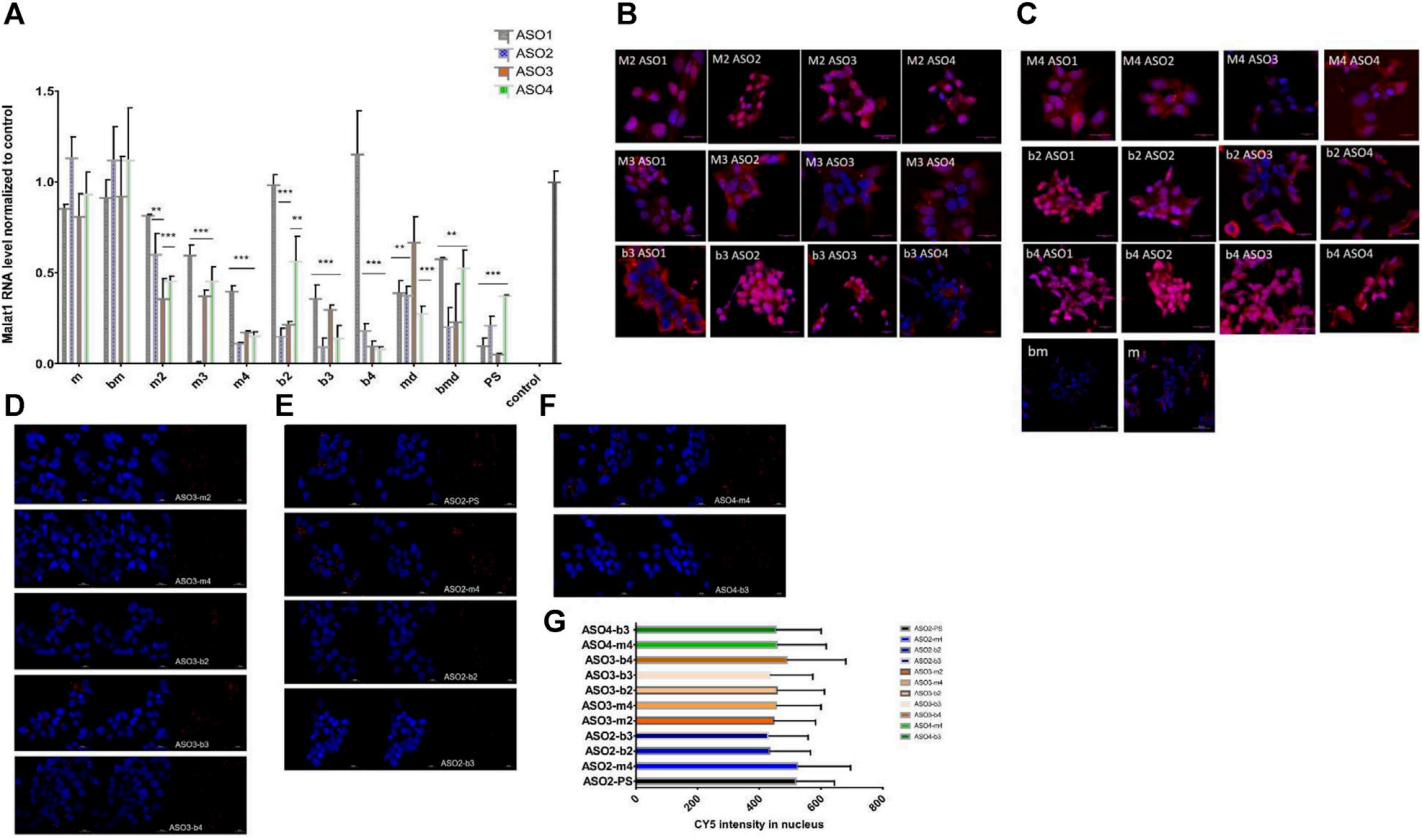
**(A)** Activity of mesyl and busyl ASOs in 22Rv1 cells. mRNA GAPDH was used as a reference gene. Results show mean ± SD. **p < 0.01 and ***p < 0.001. **(B,C)** Confocal microscopy images (z-stacks) for ASO internalization in fixed 22Rv1 cells. Cy5-labeled ASO are in red, DAPI-stained cell nuclei are in blue. **(D–F)** Confocal microscopy images of modified ASOs internalized in living 22Rv1 cells. Panels: **(D)** ASO2, **(E)** ASO3 and **(F)** ASO4, respectively; PS–phosphorothioate, m–mesyl, and b, busyl modified oligonucleotides named as in Figure 1A. Cy5-labeled ASO are shown in red, DAPI-stained cell nuclei are shown in blue. **(G)** Graph of the calculated Cy5 intensity in the cell nucleus.

After initial evaluation of ASOs containing mesyl and busyl phosphoramidate groups, we studied Malat1 RNA knockdown (KD) in prostate cancer cells 22Rv1. ASOs (10 nM) were transfected in 22Rv1 cells using lipofectamine, and the level of Malat1 RNA was analyzed by RT-qPCR in 24 h post-transfection (Figure 2A). As a control we used an oligonucleotide with random sequence (scr-ASO1, Supplementary Table S1).

Transfection of the cells with all-mesyl or busyl phosphoramidate-modified ASOs (m and bm) at a concentration of 10 nM did not lead to any noticeable decrease of Malat1 RNA level. Despite promising results in the RNase H cleavage assay for these ASOs, they were nearly inactive in the cell contrary to most gapmer combinations. Based on the results of RT-qPCR, we can conclude that the activity of ASO with the same pattern of modifications strongly depended on the sequence of an ASO. All-PS ASO1–3 showed similar activity in Malat1 depletion (∼80%), while similar patterns of mesyl and busyl modifications changed their potency in rather different directions. Mesyl and busyl modified ASO1 became less active in comparison to PS. At the same time all-PS ASO4 was the least active in comparison to other PS ASOs, whereas its mesyl and busyl versions were comparable to ASO1 in KD efficacy. All-PS ASO2 was one of the most active, and most ASO2 variants with mesyl and busyl groups also showed high KD (m3, m4, b2, b3, b4), whereas md and bmd modified all-DNA ASOs demonstrated lower activity compared to others with average depletion level of around 50%. Patterns of modifications m3, m4, b2, b3, b4 with a PS DNA gap and one to four mesyl or busyl modifications in the 2′-OMe wings demonstrated comparable activity with PS ASO. Thus m3, m4, b2, b3, b4 patterns of ASO modifications may be used as an alternative to all-phosphorothioate gapmer modifications.

### 2.4 Uptake of mesyl and busyl phosphoramidate modified ASOs into prostate cancer cell

To clarify discordant results between RNase H assay and KD *in vitro*, we studied intracellular distribution and nuclear uptake of Cy5-labeled mesyl and busyl ASOs after passive uptake (gymnosis) at 1 μM concentration and 24 h exposure in 22Rv1 cells (Figures 2B, C). Confocal micrographs confirm that fully modified mesyl or busyl ASOs were not localized in the cell nucleus, since we observed no fluorescent signal (Figure 2C, m and bm). Since Malat1 is mainly localized in the nucleus (Dong et al., 2017), this observation explains the absence of Malat1 RNA downregulation in the case of ASO with m and bm pattern of modification. For others modified ASOs (m2, m3, m4, b2, b3, b4) we observed fluorescence in the cell nuclei. Based on the previous results, we selected ASO2-PS, ASO2-m4, ASO2-b2, ASO2-b3, ASO3-m2, ASO3-m4, ASO3-b2, ASO3-b3, ASO3-b4, ASO4-m4, ASO4-b3 for the studies of intracellular distribution in live 22Rv1 cells after passive uptake (gymnosis) during 16 h at 0.1 μM concentration. For all these ASOs we observed nuclear localization by fluorescent microscopy (Figures 2D–F). The fluorescence level of Cy5-labeled mesyl and busyl modified ASOs was comparable to the phosphorothioate ASO (Figure 2G). Increasing the number of mesyl or busyl modifications per oligonucleotide slightly enhanced the fluorescent signal in the nucleus.

### 2.5 Receptor-mediated delivery of mesyl and busyl phosphoramidate modified ASOs conjugated with PSMA ligands to prostate cancer cells

**FIGURE 3:**
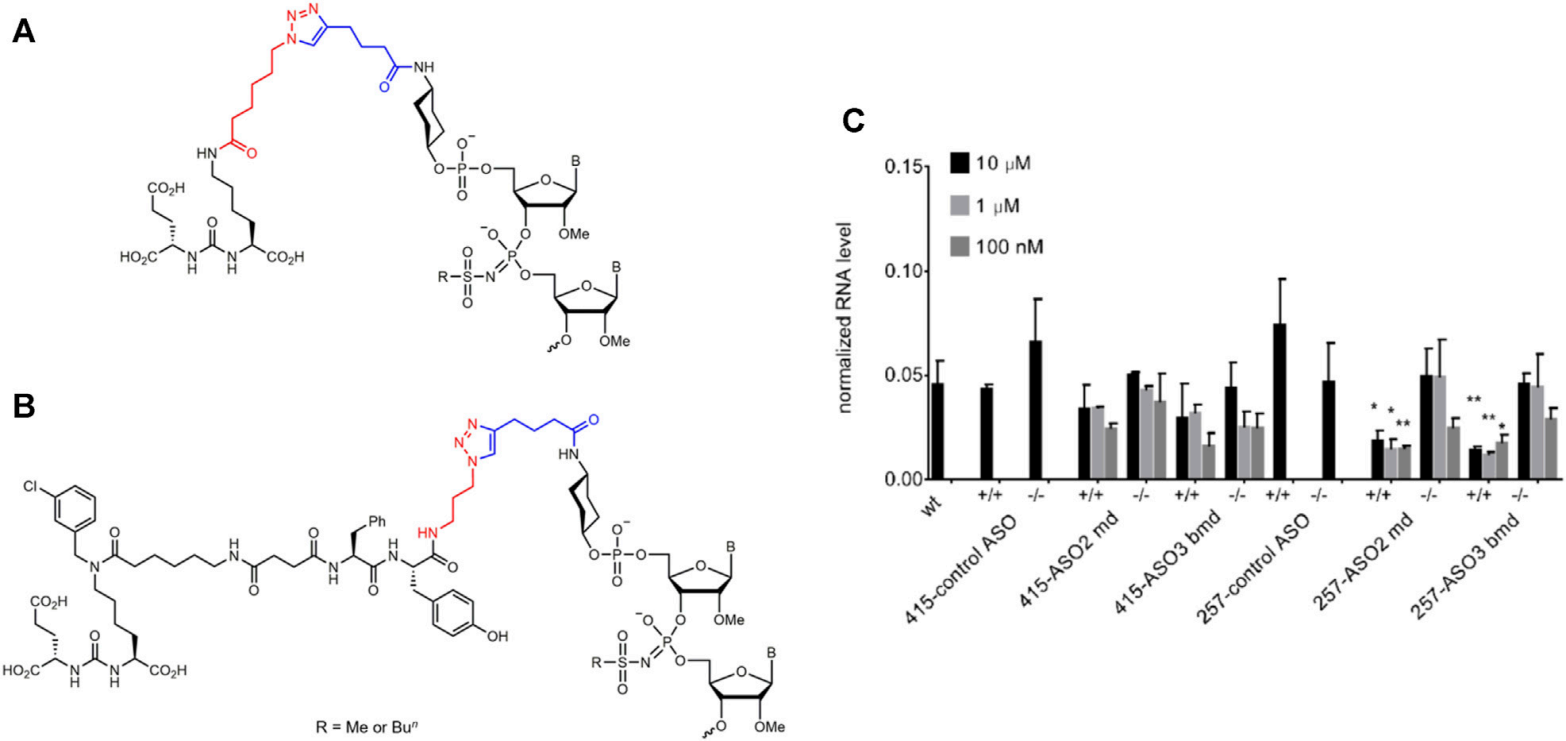
Schemes for the attachment of the PSMA ligands MA-415 **(A)** and MA-257 **(B)** to the 5′-end of a mesyl or busyl oligonucleotide via copper (I)-catalyzed azide-alkyne 1,3-dipolar cycloaddition (CuAAC). Highlighted in color are the respective azide (red) and alkyne (blue) fragments. B- nucleobase. **(C)** KD of Malat1 by fully mesyl (md) or mixed mesyl (gap) and busyl (wings) all-deoxy ASOs conjugated with PSMA ligands MA-257 and MA-415 in PSMA^+/+^ and PSMA^−/−^ 22Rv1 cells. GAPDH mRNA was used as a reference gene. Results show mean ± SD. *p < 0.05 and **p < 0.01.

Since PSMA is overexpressed on the surface of prostate cancer cells, various ligands targeting PSMA were used for targeted delivery to these cells (Tateishi, 2020; Stetsenko, 2023). We selected two variants of PSMA ligands, namely, MA-257 and MA-415 (Supplementary Figure S2) validated in previous works (Machulkin et al., 2019; Machulkin et al., 2021). MA-257 and MA-415 derivatives containing an azido group were conjugated with 5′-alkynyl ASOs of the md and bmd series (ASO2; ASO3; ASO4) by copper (I)-catalysed azide-alkyne 1,3-dipolar cycloaddition chemistry (CuAAC) (Farzan et al., 2017). Under these conditions we observed significant desulfurization of the all-PS ASO, so we were unable to get pure MA-257 and MA-415 conjugates for this ASO. Since the decrease of PS content may compromise ASO stability to nucleases, we decided not to use these mixed PS/PO oligonucleotide conjugates as controls. Therefore, we prepared two alternative control conjugates using random sequences and bmd pattern of chemical modifications. We evaluated the activity of PSMA-ASO conjugates (100 nM—10 µM) in the 22Rv1 cell line with the high level of PSMA expression (PSMA^+/+^) in comparison with CRISPR/Cas9 engineered 22Rv1 cell line with PSMA deletion (PSMA^−/−^) (Supplementary Figure S3), prepared as described previously (Caromile et al., 2017b). After 72 h, total RNA was isolated from PSMA^+/+^ and PSMA^−/−^ cells, and the efficacy of Malat1 depletion was measured by RT-qPCR (Figure 3).

We found that the conjugates of ASO2-md and ASO3-bmd with PSMA ligand 257 demonstrated specific activity in PSMA^+/+^ cells, and were not active in PSMA^−/−^ cells. The efficacy of Malat1 depletion was more than 70% (Figure 3). Also, the conjugates of ASO2-md and ASO3-bmd with PSMA ligand 257 demonstrated similar activity under all studied concentrations. At the same time, ASO conjugates with PSMA ligand 415 did not show statistically significant activity more than 50% in comparison to the control conjugate. Thus, we demonstrated selective depletion of Malat1 lncRNA by mesyl or busyl ASO conjugates with a PSMA ligand in PSMA^+/+^ prostate cancer cells.

## 3 Discussion

The phosphorothioate group (PS) is one of the most common modifications in RNA therapeutics since it stabilizes an oligonucleotide towards nucleolytic degradation, and supports RNase H-mediated digestion of the ASO-bound RNA target (Eckstein, 2014). However, avid non-specific binding of PS oligonucleotides to proteins, although it conveys beneficial PK/PD properties, at the same time can result in the increased toxicity and pro-inflammatory effects (Migawa et al., 2019; Shen et al., 2019; Crooke et al., 2021a). Consequently, new chemical modifications that can modulate ASOs properties without perturbing RNase H recruitment and stability in comparison to PS will be a significant improvement for antisense technology. Recent applications of phosphoryl guanidine (Kupryushkin et al, 2014), mesyl (Burakova et al., 2019) and busyl (Caromile et al., 2017a) phosphoramidate groups clearly demonstrate considerable benefits of their use for modification of oligonucleotides. The overall lipophilicity of new modified oligonucleotides increases from the μ to β modification, with PS somewhere in between of the two. However, the enhanced protein binding in the case of the phosphorothioate ASOs cannot be attributed to just the lipophilicity alone; this phenomenon is more complex and is more to do with the ‘soft’ polarizable nature of the sulfur atom in the PS group (Liang et al., 2015). The better cellular uptake of PS oligonucleotides, in particular, has been recently ascribed to the interaction with cystine-containing proteins via transient disulfide bridges formation (Liang et al., 2015; Crooke et al., 2017; Shen et al., 2015). Fully busylated oligonucleotides have better cellular uptake than fully mesylated ones (Burakova et al., 2019), yet, it has not translated into higher *in vivo* activity at least in the case of a splice-switching application (Hammond et al., 2021). We can assume that protein binding would be lower for both busyl and, especially, mesyl than for the phosphorothioate in the fully substituted oligonucleotides. With a mixed patterm of modification, of course, it will be more complex. The available data suggests that an optimal balance, e.g., of mesyl and PS groups can result in a marked increase in the overall potency (Anderson et al., 2021). However, further studies are required to ascertain specific protein binding properties of the sulfonylated vs. thioated oligos, especially *versus* human plasma proteins, which will have significant impact on pharmacokinetics.

In this work, we have studied the influence of replacing some or all PS groups in all-deoxy or gapmer ASOs with mesyl (μ) and busyl (β) phosphoramidate modifications (Figure 1A) on the RNase H cleavage pattern of RNA targets, cellular localization of ASO in prostate cancer cells, and their potency in comparison to the classic all-phosphorothioate gapmer ASOs. We designed and synthesized sets of ASOs with different patterns of the μ, β, and PS modifications from one mesyl or busyl group at the tip of each wing of a gapmer to the fully modified oligomers (Figure 1B). Also, we synthesized μ- and β-modified all-DNA ASOs (md and bmd, respectively). We demonstrated that addition of mesyl and busyl modifications in ASO wings moved the specificity of RNase H-mediated cleavage to the 18–19th position of the Malat1 template (m2-m4, b2-b4) in comparison with 19–20th position for the PS ASOs. If the μ and β modifications were included in the DNA gap (bmd, md, bm, m), the additional cleavage appeared after the 17th nucleotide (Figures 1D–G). Thus, the increase of the number of mesyl and busyl groups in the wings, and addition of the modifications in the gap changed the RNase H cleavage pattern in comparison to the PS ASOs. Our results correlate with previously published data from Ionis Pharmaceuticals that the introduction of two mesyl phosphoramidate groups at the 3′- or 5′-ends of an ASO away from the DNA gap had a minimal effect on the pattern of cleavage. At the same time, a μ group in the DNA gap resulted in a site-specific impact on the cleavage pattern (Anderson et al., 2021). These results are contradictory to a previous report that uniformly μ-substituted DNA ASOs had improved RNase H activation relative to PS DNA ASOs (Miroshnichenko et al., 2019). We want to emphasize that RNA duplexes with fully modified 2′-OMe or 2′-MOE RNA, or locked nucleic acids (LNA) are not the substrates for RNase H (Eckstein, 2014), so a direct comparison may be irrelevant. Previous studies on mesyl phosphoramidate oligonucleotides have demonstrated a similar binding affinity to either DNA or RNA as for the natural oligonucleotides by either thermal melting studies or gel retardation assay (Chelobanov et al., 2017; Miroshnichenko et al., 2019). Independently, a similar RNA affifinity for mesyl ASOs as for all-PS ASOs was confirmed (Anderson et al., 2021). These datasets convincingly demonstrated that a mesyl phosphoramidate group can be relatively seamlessly combined with sugar modifications without adverse impact on the melting temperature of ASO: RNA duplexes. Therefore, no thermal melting experiments for the ASOs were included in this particular study. However, we do plan to compare the RNA binding affinity of the PS gapmers vs. mesyl/busyl modified gapmers in future.

Cellular uptake and intracellular localization of ASO is driven by endocytosis and trafficking, and is mediated by interactions with many proteins. More than 60 proteins can interact with ASO and modulate their activity in the cell (Crooke et al., 2017). Several proteins such as Ku70, Ku80, P54nrb, and hnRNPs compete with RNase H for the binding to ASO, which can reduce the activity and affect subcellular localization of ASOs (Liang et al., 2015; Shen et al., 2015). We found that several mesyl/busyl phosphoramidate groups in Cy5-labeled ASO did not have a negative influence on the cellular uptake and intranuclear localization in live prostate cancer cells (Figures 2D–G). Further increase of the number of μ or β modifications in the ASO slightly enhanced the fluorescent signal in the nucleus. However, fully modified ASOs demonstrated only cytoplasmic localization, there was no signal in the cell nucleus (Figure 2C, m and bm). Previously, it was demonstrated that an increase in hydrophobicity of the ASO either in the 2′-deoxy → 2′-OMe → 2′-MOE row, or from mesyl to busyl had affected the cellular uptake, endosomal release, and intracellular distribution of the ASOs including nuclear localization (Shen et al., 2015).

Inhibitory activity of mesyl and busyl phosphoramidate ASOs was tested in prostate cancer cells in comparison with PS gapmer ASOs. Fully modified (m and bm) gapmer ASOs were nearly inactive in the cells at 10 nM concentration (Figure 2A). These findings seem to be somewhat discordant with earlier data where all-mesyl DNA ASOs showed improved activity relative to all-PS DNA ASOs although the latter data were obtained at higher concentrations of 100–200 nM (Miroshnichenko et al., 2019), but correlate with previously published results that replacing nine or more PS with mesyl phosphoramidate groups from either side of the gap resulted in a significant reduction of the potency (Anderson et al., 2021). Malat1 lncRNA is mainly localized in the nuclear speckles, whereas m and bm modified ASOs demonstrated poor nuclear localization, which may contribute to lower activity. We found that the ASO sequence influences the activity even within the same pattern of chemical modifications. Gapmer ASOs with all-phosphorothioate DNA gap and one to four mesyl or busyl modifications in the RNA wings had similar or sometimes better activity as control PS ASOs in the prostate cancer cells (Figure 2A). Thus, m3, m4, b2, b3, b4 patterns of ASO modification can be used as an alternative to all-phosphorothioate gapmers without compromising potency. Similar results were obtained previously for the mesyl/busyl modified ASO acted as a splice switching and anticancer therapeutics. Peritumoral injection of all-mesyl modified ASOs resulted in efficient accumulation in the tumor (Miroshnichenko et al., 2019; Patutina et al., 2020). Also it was shown that up to five μ-modifications near 5′-side of the gap in PS gapmers showed similar or improved activity and decreased pro-inflammatory effects, but uniform modification with mesyl groups across the entire sequence or just in the DNA gap resulted in a reduced potency (Anderson et al., 2021). Thus, a combination of phosphorothioates in the gap and mesyl or busyl phosphoramidates in the wings of a gapmer may lead to improved efficacy, reduced inflammation, and increased stability in the design of future ASOs (Miroshnichenko et al., 2019; Patutina et al., 2020; Anderson et al., 2021). In particular, one of the sequences tested herein, namely, ASO2/m3 seems to have exceptionally high activity towards Malat1 lncRNA (Figure 2), which certainly warrants further investigation. We plan to evaluate it in a dose-dependent manner in comparison to all-PS ASO2.

Prostate-specific membrane antigen (PSMA), a prostate cancer specific cell surface biomarker, can be used for prostate cancer targeting, since it is highly overexpressed in nearly all types of prostate cancer but demonstrated only limited expression in normal tissues. To achieve highly efficient binding to PSMA for improved cellular uptake, small molecule PSMA ligands have been developed, such as DUPA for the delivery of siRNAs to PLK1 (Abdelaal and Kasinski, 2021), or KUE for the delivery of siRNAs to STAT3 (Yang et al., 2021). However, numerous studies on the optimization of the structure and properties of the linker in the PSMA ligand resulted in a significant improvement in the affinity and pharmacokinetic parameters of PSMA conjugates (Baranski et al., 2017). Fully mesyl or mixed mesyl (gap) and busyl (wings) all-deoxy ASOs (md and bmd, respectively) were conjugated with two variants of the previously validated PSMA ligands, namely, MA-257 and MA-415 (Machulkin et al., 2019; Machulkin et al., 2021) for targeted delivery to prostate cancer cells. We observed efficient and selective depletion of Malat1 lncRNA in PSMA^+/+^ cancer cells with almost no activity in PSMA^−/−^ cells for AS02-md and ASO3-bmd conjugates with PSMA ligand MA-257 at concentrations from 100 nM to 10 μM, which confirms the promise of mesyl and busyl phosphoramidate ASO modifications for the targeted RNA therapy. However, conjugation of the classical MA-415 PSMA derivative did not lead to any significant improvement of targeted delivery *in vitro* in comparison with naked ASOs.

In conclusion, the introduction of the mesyl (μ) and busyl (β) phosphoramidate groups into ASOs did not significantly influence the RNase H activity but changed the cleavage pattern depending on the ASO sequence and positions of the modified phosphates. Fully mesyl and busyl modified ASO gapmers were nearly inactive in prostate cancer cells at 10 nM possibly due to poorer intranuclear localization, yet partially modified gapmers had similar or even improved activity in comparison with all-PS ASO *in vitro*. We studied PSMA ligand-mediated delivery of all-deoxy ASOs having either mesyl (md) or a mesyl and busyl combination (bmd) groups, and no phosphorothioate groups at all, and observed efficient depletion of the Malat1 lncRNA *in vitro*. Thus, we combined recently developed mesyl and busyl phosphoramidate ASO modifications with PSMA ligand conjugation, and uncovered an efficient alternative to the classical all-phosphorothioate gapmer ASOs with improved targeted delivery to prostate cancer cells.

## 4 Materials and methods

### 4.1 Design and synthesis of oligonucleotides

Four previously validated ASO sequences targeting Malat1 (Gutschner et al., 2013; Dong et al., 2017; Liang et al., 2017; Ämmälä et al., 2018) were selected, and a pattern of mesyl and busyl modifications and combinations with other chemical modifications were designed. The synthesis of 2′-*O*-methyl mesyl (μ) and busyl (β) phosphoramidate oligonucleotides (Supplementary Table S1) was done according to our previously published method of automated solid-phase phosphoramidite synthesis replacing aqueous iodine oxidation with Staudinger reaction with either methanesulfonyl azide or 1-butanesulfonyl azide in each cycle of chain elongation (Caromile et al., 2017a; Miroshnichenko et al., 2019). The Staudinger reaction between polymer-bound phosphite triester formed upon phosphoramidite coupling, and methanesulfonyl azide or 1-butanesulfonyl azide (0.5 M in acetonitrile), respectively, was carried out for 40 min at ambient temperature. Oligonucleotides containing 5′-*O*-dimethoxytrityl group (DMTr) were isolated by reverse-phased (RP) HPLC and, if necessary, purified to homogeneity by denaturing PAGE under the same conditions as phosphodiester or PS ONs followed by RP-HPLC. The molecular masses of the µ- and β-oligonucleotides obtained have been confirmed by ESI LC-MS.

For confocal microscopy studies, 3′-alkynylated mesyl and busyl phosphoramidate oligonucleotides were labeled with Cy5 azide using Cu(I)-catalyzed 1,3-dipolar alkyne-azide cycloaddition reaction (CuAAC) (Farzan et al., 2017). As PS oligonucleotides have been reported to undergo significant desulfurization under Cu(I)-mediated click reaction (Honcharenko et al., 2019), *N*-hydroxysuccinimidyl (NHS) ester of Cy5 carboxylic acid dye has been used for acylation of 3′-aminoalkylated oligonucleotide in the latter case. Molecular masses of the oligonucleotides were verified by LC-ESI-MS (Supplementary Table S1).

### 4.2 Determination of RNAse H cleavage efficacy

The RNA template (2 pmol) was mixed with the ASO (1:3 ratio), and preheated at 37°C for 5 min in annealing buffer of 100 mM KCl, 0.1 mM EDTA. Then 10 U of *E. coli* RNase H enzyme (NEB, MA, USA) was added in the reaction buffer. The mixture was incubated at 37°C for 1 h, then put on ice. An aliquote (5 Μl) of the reaction mixture was mixed with 15 μL of the RNA loading buffer (95% formamide, 0.025% SDS, 0.5 mM EDTA), and incubated at 95°C for 5 min to destroy any secondary structures of the RNA or ASO. After that, 5 μL was loaded on a polyacrylamide gel (1:19, 15%). Electrophoresis was carried out and the gel was photographed using Bio-Rad ChemiDoc™ MP Imager. The images were processed using ImageJ™ software, and analyzed by GelAnalyzer™. The cleavage efficacy was calculated as the intensity of each band in the gel divided on the sum of all bands’ intensities (Supplementary Figure S1).

### 4.3 Evaluation of inhibitory activity of modified ASOs in 22Rv1 prostate cancer cells

22Rv1 cells (CRL-2505) were cultured in RPMI-1640 medium (ThermoFisher Scientific, MA, USA), 10% fetal bovine serum (FBS), 1% penicillin-streptomycin at 37°C, and 5% CO_2_ in a 6-well plate for 24 h. After 24 h cells were washed with PBS and transfeсted with 10 nM ASO using lipofectamine RNAiMAX transfection reagent (ThermoFisher Scientific, MA, USA) premixed in OptiMEM I Reduced Serum Medium (ThermoFisher Scientific, MA, USA) according to the manufacturer’s protocol. As a control, we used an ASO with a random sequence (scr-ASO1, Supplementary Table S1). Cells were incubated for 24 h before RNA isolation. Total RNA was isolated using TRIzol (ThermoFisher Scientific, MA, USA) according to the manufacturer’s instructions. Then, ∼0.5–1 μg of RNA was further treated with DNase I (ThermoFisher Scientific, MA, USA), supplied with RiboLock RNase Inhibitor (40 U/μL) to the final concentration of 0.4 U/μL. For RT-qPCR, treated total RNA was used to synthesize cDNA using Maxima First Strand cDNA Synthesis Kit, followed by qPCR using PowerUp™ SYBR™ Green Master Mix (ThermoFisher Scientific, MA, USA) according to the manufacturer’s protocol. GAPDH mRNA was used as a control. We used Malat1 Forward primer 5′-d(AACCAGTTTCCCCAGCTTTT), Malat1 Reverse primer 5′-d(CTACATTCCCACCCAGCACT), human GAPDH Forward primer 5′-d(GTCTCCTCTGACTTCAACAGCG) and human GAPDH Reverse primer 5′-d(ACCACCCTGTTGCTGTAGCCAA). Statistical analyses were conducted with Prism v.8 GraphPad Software, which was also used to plot the figures.

### 4.4 Analysis of nuclear uptake of Cy5-labeled oligonucleotides by confocal microscopy in fixed and live 22Rv1 cells

Solutions of Cy5-labeled ASOs in 1× phosphate-buffered saline buffer (PBS) were added to the final concentration of 1 µM to human 22Rv1 cells in RPMI-1640 medium supplemented with 10% FBS, 100 μg/mL streptomycin/penicillin, cultured on poly-L-lysine coated cover glass slides placed in the bottom of a 24-well plate. Cells were cultured at 37°C in humidified air with 5% CO_2_ for 24 h. Then glasses with cells were washed with 1×PBS, fixed in 1×PBS containing 4% formaldehyde at room temperature for 20 min, washed with 1×PBS, and permeabilized in 1×PBS with 0.1% Triton X-100 for 5 min. Then glasses were washed twice with 1×PBS for 10 min. Cells were embedded in Mowiol with DAPI (Sigma, MO, USA) prior to imaging. Confocal microscopy was performed using Nikon A1+MP confocal imaging system using a Plan Apo 20x/0,75 Dic N objective (numerical aperture 0.75, Nikon, Japan), Apo LWD 40x/1,15 S water immersion objective (numerical aperture 0.15, Nikon, Japan) and Apo tirf 60x/1,49 DIC oil immersion objective (numerical aperture 0.49, Nikon, Japan). Images were scanned sequentially using 561 nm diode lasers in combination with a DM561 nm dichroic beam splitter. To describe the nuclear membrane contour, each Z-stack was processed by the intensity threshold of the blue channel (cell nucleus) in the first stage of calculations. Thresholding was performed by the following parameters: area, roundness, intensity of fluorescence. Next, the binary layer of nuclei was subtracted from the binary layer of the sample, allowing to detect fluorescence intensity of the ASOs inside the nuclei only.

For the live cells imaging, 1 × 105 22Rv1 cells in RPMI-1640 medium supplemented with 10% FBS, 100 μg/mL streptomycin/penicillin cultured in the confocal dishes 24 h up to achieving 70% confluence. Selected ASOs at 0.1 μM concentration were studied after unaided uptake (gymnosis) during 16 h. The cells were washed 3 times with 1×PBS, and the fresh RPMI-1640 medium with DAPI was added. Live cell imaging of ASO internalization was carried out at 37°C in humidified air with 5% CO2 on a Nikon A1+MP confocal imaging system using a Plan Apo 20x/0,75 Dic N objective (numerical aperture 0.75, Nikon, Japan), and an Apo LWD 40x/1,15 S water immersion objective (numerical aperture 1.15, Nikon, Japan). Images were scanned sequentially using 561 nm diode lasers in combination with a DM405/488/561 nm dichroic beam splitter. Differential Interference Contrast Imaging (DIC) microscopy was used to visualize cell contours. The images were analyzed with NIS-elements AR (Nikon, Japan).

### 4.5 *In vitro* evaluation of PSMA ligand conjugates with all-deoxy ASOs containing either all-mesyl or mixed mesyl (gap) and busyl (wings) groups in prostate cancer cells

Since PSMA receptor is overexpressed on the surface of prostate cancer cells, a number of PSMA ligands were used for targeted delivery of various drugs to these cells (Ämmälä et al., 2018). We selected two variants of PSMA ligands, namely, MA-257 and MA-415 (Supplementary Figure S2), which were validated in previous works (Machulkin et al., 2019; Machulkin et al., 2021). MA-415 is an azido derivative of the most common PSMA ligand that was clinically validated for radiotherapy (Machulkin et al., 2019; Hoshi et al., 2023). The key point of the more advanced MA-257 ligand is a linker bearing a set of aromatic residues that improves binding to the PSMA receptor due to hydrophobic interactions with the channel (Machulkin et al., 2021). Azido derivatives of the ligands: 6-azidohexanoyl for MA-415 (Figure 3A) and 3-azidopropyl for MA-257 (Figure 3B), respectively, were conjugated with hexynoylated at the corresponding 5′-amino-modifier residue ASOs of the md and bmd series (ASO2; ASO3; ASO4) by copper (I)-catalysed azide-alkyne 1,3-dipolar cycloaddition chemistry (CuAAC) as described previously (Farzan et al., 2017). Briefly, we prepared Cu(I)-TBTA complex by *in situ* reduction of CuSO_4_ with ascorbic acid for CuAAC, and full conversion was achieved in aq. DMSO mixture after overnight incubation. Since peptidomimetic ligands can form stable complexes with copper ions that can induce additional toxicity, we incubated reaction mixture with 100 mM EDTA for 3 h before RP-HPLC purification to trap any Cu^+^. Structures of oligonucleotide conjugates as well as examples of LC-UV traces and ESI-MS spectra are shown in Supplementary Figure S3. Under these conditions we observed significant desulfurization of the all-PS ASO, so we were unable to get pure MA-257 and MA-415 conjugates for this ASO. Since the decrease of PS content may compromise ASO resistance to nuclease digestion, we decided not to use the resulting mixed PS/PO oligonucleotide conjugates as controls.

The activity of the PSMA-ASO conjugates were tested in the 22Rv1 cell line with high level of PSMA expression (PSMA^+/+^) in comparison with generated 22Rv1 cell line with the deletion of PSMA (PSMA^−/−^) (Suppl. Fig. 3) (Caromile et al., 2017b). PSMA^−/−^ cell line was previously generated from 22Rv1with the CRISPR/Cas9 using sgRNA: 5′-r(CAGCCGAGUCGGUUUCGUGA), which was cloned into the sgRNA scaffold of the pX458 plasmid (Addgene plasmid #48138) according to the Zhang Lab general cloning protocol. Generated clones were validated by Sanger sequencing and RT-qPCR (Supplementary Figure S3).

For the testing of the PSMA-ASO conjugates activity, PSMA^+/+^ and PSMA^−/−^ cells were cultured in RPMI-1640 medium (Thermo Scientific, MA, USA), 10% fetal bovine serum (FBS), 1% penicillin-streptomycin at 37°C and 5% CO2 in 12-well plate and then different concentration of conjugates were added to the cells: 100 nM, 1 μM, 10 µM. After 72 h cells were washed with 1x PBS and total RNA was isolated from PSMA^+/+^ and PSMA^−/−^ cells using TRIzol (ThermoFisher Scientific, MA, USA) according to the manufacturer’s instructions. Then, ∼0.5–1 μg of RNA was further treated with DNase I (ThermoFisher Scientific, MA, USA), supplied with RiboLock RNase Inhibitor (40 U/μL) to the final concentration of 0.4 U/μL. For RT-qPCR, treated total RNA was used to synthesize cDNA using Maxima First Strand cDNA Synthesis Kit, followed by qPCR using PowerUp™ SYBR™ Green Master Mix (ThermoFisher Scientific, MA, USA) according to the manufacturer protocol. GAPDH mRNA was used as a control. We used Malat1 Forward primer 5′-d(AACCAGTTTCCCCAGCTTTT), Malat1 Reverse primer 5′-d(CTACATTCCCACCCAGCACT), human GAPDH Forward primer 5′-d(GTCTCCTCTGACTTCAACAGCG) and human GAPDH Reverse primer 5′-d(ACCACCCTGTTGCTGTAGCCAA). Statistical analyses were conducted with Prism v.8, GraphPad Software, which was also used to plot the figures.

## Data Availability

The original contributions presented in the study are included in the article/Supplementary Material, further inquiries can be directed to the corresponding author.
