# Supplementary material for: Structure-activity relationship study of mesyl and busyl phosphoramidate antisense oligonucleotides for unaided and PSMA-mediated uptake into prostate cancer cells
Source: Front Chem. 2024 Mar 4;12:1342178. doi: 10.3389/fchem.2024.1342178 (PMC10944894; doi:10.3389/fchem.2024.1342178)
Supplement: Supplementary file 1 [file DataSheet1.doc]

Supplementary Material Part 1

Structure-activity relationship study of mesyl and busyl phosphoramidate antisense oligonucleotides for unaided and PSMA-mediated uptake into prostate cancer cells

**Table S1. List of ASOs used in the study and their ESI MS data.**

| **Name** | **Oligonucleotide sequence, 5′-3′** | **Molecular mass, Da** | |
| --- | --- | --- | --- |
|  |  | **Calc. [M]** | **Exp. [M]** |
| ASO1/m2 | U^µ^G^µ^C^µ^C^µ^U_s_t_s_U_s_a_s_g_s_g_s_a_s_t_s_t_s_c_s_t_s_A^µ^G^µ^A^µ^C^µ^A-NH_2_ | 7381.9 | 7383.2 |
| ASO2/m2 | C^µ^C^µ^A^µ^G^µ^G_s_c_s_U_s_g_s_g_s_t_s_t_s_a_s_t_s_g_s_a_s_C^µ^U^µ^C^µ^A^µ^G-NH_2_ | 7422.6 | 7423.2 |
| ASO3/m2 | G^µ^G^µ^G^µ^A^µ^G_s_t_s_U_s_a_s_c_s_t_s_t_s_g_s_c_s_c_s_a_s_A^µ^C^µ^U^µ^U^µ^G-NH_2_ | 7423.8 | 7425.0 |
| ASO4/m2 | A^µ^U^µ^G^µ^G^µ^A_s_g_s_G_s_t_s_a_s_t_s_g_s_a_s_c_s_a_s_t_s_A^µ^U^µ^A^µ^A^µ^U-NH_2_ | 7479.7 | 7481.2 |
| scr-ASO1/m2 | G^µ^U^µ^U^µ^A^µ^G_s_t_s_G_s_a_s_t_s_a_s_c_s_g_s_a_s_t_s_g^µ^A^µ^U^µ^A^µ^A^µ^A-NH_2_ | 7539.7 | 7541.4 |
| ASO1/m3 | U^µ^G^µ^C^µ^C_s_U_s_t_s_U_s_a_s_g_s_g_s_a_s_t_s_t_s_c_s_t_s_A_s_G^µ^A^µ^C^µ^A-NH_2_ | 7260.6 | 7262.0 |
| ASO2/m3 | C^µ^C^µ^A^µ^G_s_G_s_c_s_U_s_g_s_g_s_t_s_t_s_a_s_t_s_g_s_a_s_C_s_U^µ^C^µ^A^µ^G-NH_2_ | 7299.8 | 7301.8 |
| ASO3/m3 | G^µ^G^µ^G^µ^A_s_G_s_t_s_U_s_a_s_c_s_t_s_t_s_g_s_c_s_c_s_a_s_A_s_C^µ^U^µ^U^µ^G-NH_2_ | 7300 | 7302.1 |
| ASO4/m3 | A^µ^U^µ^G^µ^G_s_A_s_g_s_G_s_t_s_a_s_t_s_g_s_a_s_c_s_a_s_t_s_A_s_U^µ^A^µ^A^µ^U-NH_2_ | 7357.1 | 7358.3 |
| scr-ASO1/m3 | G^µ^U^µ^U^µ^A_s_G_s_t_s_G_s_a_s_t_s_a_s_c_s_g_s_a_s_t_s_g_s_A_s_U^µ^A^µ^A^µ^A-NH_2_ | 7357.1 | 7358.3 |
| ASO1/m4 | U^µ^G_s_C_s_C_s_U_s_t_s_U_s_a_s_g_s_g_s_a_s_t_s_t_s_c_s_t_s_A_s_G_s_A_s_C^µ^A-NH_2_ | 7015.7 | 7017.2 |
| ASO2/m4 | C^µ^C_s_A_s_G_s_G_s_c_s_U_s_g_s_g_s_t_s_t_s_a_s_t_s_g_s_a_s_C_s_U_s_C_s_A^µ^G-NH_2_ | 7056.1 | 7057.7 |
| ASO3/m4 | G^µ^G_s_G_s_A_s_G_s_t_s_U_s_a_s_c_s_t_s_t_s_g_s_c_s_c_s_a_s_A_s_C_s_U_s_U^µ^G-NH_2_ | 7056.3 | 7057.9 |
| ASO4/m4 | A^µ^U_s_G_s_G_s_A_s_g_s_G_s_t_s_a_s_t_s_g_s_a_s_c_s_a_s_t_s_A_s_U_s_A_s_A^µ^U-NH_2_ | 7112.9 | 7114.4 |
| scr-ASO1/m4 | G^µ^U_s_U_s_A_s_G_s_t_s_G_s_a_s_t_s_a_s_c_s_g_s_a_s_t_s_g_s_A_s_U_s_A_s_A^µ^A-NH_2_ | 7112.7 | 7114.1 |
| ASO1/b2 | U^β^G^β^C^β^C^β^U_s_t_s_U_s_a_s_g_s_g_s_a_s_t_s_t_s_c_s_t_s_A^β^G^β^A^β^C^β^A-NH_2_ | 7718.7 | 7720.0 |
| ASO2/b2 | C^β^C^β^A^β^G^β^G_s_c_s_U_s_g_s_g_s_t_s_t_s_a_s_t_s_g_s_a_s_C^β^U^β^C^β^A^β^G-NH_2_ | 7758.1 | 7759.7 |
| ASO3/b2 | G^β^G^β^G^β^A^β^G_s_t_s_U_s_a_s_c_s_t_s_t_s_g_s_c_s_c_s_a_s_A^β^C^β^U^β^U^β^G-NH_2_ | 7759.9 | 7761.4 |
| ASO4/b2 | A^β^U^β^G^β^G^β^A_s_g_s_G_s_t_s_a_s_t_s_g_s_a_s_c_s_a_s_t_s_A^β^U^β^A^β^A^β^U-NH_2_ | 7816.1 | 7817.5 |
| scr-ASO1/b2 | G^β^U^β^U^β^A^β^G_s_t_s_G_s_a_s_t_s_a_s_c_s_g_s_a_s_t_s_g_s_A^β^U^β^A^β^A^β^A-NH_2_ | 7717.9 | 7919.3 |
| ASO1/b3 | U^β^G^β^C^β^C_s_U_s_t_s_U_s_a_s_g_s_g_s_a_s_t_s_t_s_c_s_t_s_A_s_G^β^A^β^C^β^A-NH_2_ | 7512.4 | 7513.9 |
| ASO2/b3 | C^β^C^β^A^β^G_s_G_s_c_s_U_s_g_s_g_s_t_s_t_s_a_s_t_s_g_s_a_s_C_s_U^β^C^β^A^β^G-NH_2_ | 7552.2 | 7553.8 |
| ASO3/b3 | G^β^G^β^G^β^A_s_G_s_t_s_U_s_a_s_c_s_t_s_t_s_g_s_c_s_c_s_a_s_A_s_C^β^U^β^U^β^G-NH_2_ | 7554.1 | 7554.5 |
| ASO4/b3 | A^β^U^β^G^β^G_s_A_s_g_s_G_s_t_s_a_s_t_s_g_s_a_s_c_s_a_s_t_s_A_s_U^β^A^β^A^β^U-NH_2_ | 7609.4 | 7611.2 |
| scr-ASO1/b3 | G^β^U^β^U^β^A_s_G_s_t_s_G_s_a_s_t_s_a_s_c_s_g_s_a_s_t_s_g_s_A_s_U^β^A^β^A^β^A-NH_2_ | 7614.6 | 7611.0 |
| ASO1/b4 | U^β^G_s_C_s_C_s_U_s_t_s_U_s_a_s_g_s_g_s_a_s_t_s_t_s_c_s_t_s_A_s_G_s_A_s_C^β^A-NH_2_ | 7099.9 | 7101.3 |
| ASO2/b4 | C^β^C_s_A_s_G_s_G_s_c_s_U_s_g_s_g_s_t_s_t_s_a_s_t_s_g_s_a_s_C_s_U_s_C_s_A^β^G-NH_2_ | 7140.3 | 7141.9 |
| ASO3/b4 | G^β^G_s_G_s_A_s_G_s_t_s_U_s_a_s_c_s_t_s_t_s_g_s_c_s_c_s_a_s_A_s_C_s_U_s_U^β^G-NH_2_ | 7141.3 | 7142.7 |
| ASO4/b4 | A^β^U_s_G_s_G_s_A_s_g_s_G_s_t_s_a_s_t_s_g_s_a_s_c_s_a_s_t_s_A_s_U_s_A_s_A^β^U-NH_2_ | 7197.4 | 7198.7 |
| scr-ASO1/b4 | G^β^U_s_U_s_A_s_G_s_t_s_G_s_a_s_t_s_a_s_c_s_g_s_a_s_t_s_g_s_A_s_U_s_A_s_A^β^A-NH_2_ | 7197.1 | 7198.3 |
| ASO1 | Alkyne-U_s_G_s_C_s_C_s_U_s_t_s_t_s_a_s_g_s_g_s_a_s_t_s_t_s_c_s_t_s_A_s_G_s_A_s_C_s_A-NH_2_ | 7248.8 | 7250.0 |
| ASO2 | Alkyne-C_s_C_s_A_s_G_s_G_s_c_s_t_s_g_s_g_s_t_s_t_s_a_s_t_s_g_s_a_s_C_s_U_s_C_s_A_s_G-NH_2_ | 6917.4 | 6919.0 |
| ASO3 | Alkyne-G_s_G_s_G_s_A_s_G_s_t_s_t_s_a_s_c_s_t_s_t_s_g_s_c_s_c_s_a_s_A_s_C_s_U_s_U_s_G-NH_2_ | 7202.6 | 7204.4 |
| ASO4 | Alkyne-A_s_U_s_G_s_G_s_A_s_g_s_g_s_t_s_a_s_t_s_g_s_a_s_c_s_a_s_t_s_A_s_U_s_A_s_A_s_U-NH_2_ | 7165.1 | 7166.3 |
| scr-ASO1 | Alkyne-G_s_U_s_U_s_A_s_G_s_t_s_g_s_a_s_t_s_a_s_c_s_g_s_a_s_t_s_g_s_A_s_U_s_A_s_A_s_A-NH_2_ | 7248.8 | 7250.0 |
| ASO1_m | Alkyne-U^µ^G^µ^C^µ^C^µ^U^µ^t^µ^t^µ^a^µ^g^µ^g^µ^a^µ^t^µ^t^µ^c^µ^t^µ^A^µ^G^µ^A^µ^C^µ^A-NH_2_ | 8446.9 | 8448.5 |
| ASO2_m | Alkyne-C^µ^C^µ^A^µ^G^µ^G^µ^c^µ^t^µ^g^µ^g^µ^t^µ^t^µ^a^µ^t^µ^g^µ^a^µ^C^µ^U^µ^C^µ^A^µ^G-NH_2_ | 8487.1 | 8488.4 |
| ASO3_m | Alkyne-G^µ^G^µ^G^µ^A^µ^G^µ^t^µ^t^µ^a^µ^c^µ^t^µ^t^µ^g^µ^c^µ^c^µ^a^µ^A^µ^C^µ^U^µ^U^µ^G-NH_2_ | 8488.1 | 8489.4 |
| ASO4_m | Alkyne-A^µ^U^µ^G^µ^G^µ^A^µ^g^µ^g^µ^t^µ^a^µ^t^µ^g^µ^a^µ^c^µ^a^µ^t^µ^A^µ^U^µ^A^µ^A^µ^U-NH_2_ | 8529.8 | 8531.4 |
| scr-ASO1_m | Alkyne-G^µ^U^µ^U^µ^A^µ^G^µ^t^µ^g^µ^a^µ^t^µ^a^µ^c^µ^g^µ^a^µ^t^µ^g^µ^A^µ^U^µ^A^µ^A^µ^A-NH_2_ | 8530.1 | 8531.6 |
| ASO1_bm | Alkyne-U^β^G^β^C^β^C^β^U^µ^t^µ^t^µ^a^µ^g^µ^g^µ^a^µ^t^µ^t^µ^c^µ^t^β^A^β^G^β^A^β^C^β^A-NH_2_ | 8910.2 | 8911.6 |
| ASO2_bm | Alkyne-C^β^C^β^A^β^G^β^G^µ^c^µ^t^µ^g^µ^g^µ^t^µ^t^µ^a^µ^t^µ^g^µ^a^β^C^β^U^β^C^β^A^β^G-NH_2_ | 8950.0 | 8951.6 |
| ASO3_bm | Alkyne-G^β^G^β^G^β^A^β^G^µ^t^µ^t^µ^a^µ^c^µ^t^µ^t^µ^g^µ^c^µ^c^µ^a^β^A^β^C^β^U^β^U^β^G-NH_2_ | 8950.4 | 8952.0 |
| ASO4_bm | Alkyne-A^β^U^β^G^β^G^β^A^µ^g^µ^g^µ^t^µ^a^µ^t^µ^g^µ^a^µ^c^µ^a^µ^t^β^A^β^U^β^A^β^A^β^U-NH_2_ | 8992.4 | 8993.8 |
| scr-ASO1_bm | Alkyne-G^β^U^β^U^β^A^β^G^µ^t^µ^g^µ^a^µ^t^µ^a^µ^c^µ^g^µ^a^µ^t^µ^g^β^A^β^U^β^A^β^A^β^A-NH_2_ | 8992.7 | 8994.0 |
| ASO1_md | Alkyne-t^µ^g^µ^c^µ^c^µ^t^µ^t^µ^t^µ^a^µ^g^µ^g^µ^a^µ^t^µ^t^µ^c^µ^t^µ^a^µ^g^µ^a^µ^c^µ^a-NH_2_ | 7920.4 | 7922.2 |
| ASO2_md | Alkyne-c^µ^c^µ^a^µ^g^µ^g^µ^c^µ^t^µ^g^µ^g^µ^t^µ^t^µ^a^µ^t^µ^g^µ^a^µ^c^µ^t^µ^c^µ^a^µ^g-NH_2_ | 7947.1 | 7948.4 |
| ASO3_md | Alkyne-g^µ^g^µ^g^µ^a^µ^g^µ^t^µ^t^µ^a^µ^c^µ^t^µ^t^µ^g^µ^c^µ^c^µ^a^µ^a^µ^c^µ^t^µ^t^µ^g-NH_2_ | 7961.2 | 7962.6 |
| ASO4_md | Alkyne-a^µ^t^µ^g^µ^g^µ^a^µ^g^µ^g^µ^t^µ^a^µ^t^µ^g^µ^a^µ^c^µ^a^µ^t^µ^a^µ^t^µ^a^µ^a^µ^t-NH_2_ | 8017.1 | 8018.8 |
| scr-ASO1_md | Alkyne-g^µ^t^µ^t^µ^a^µ^g^µ^t^µ^g^µ^a^µ^t^µ^a^µ^c^µ^g^µ^a^µ^t^µ^g^µ^a^µ^t^µ^a^µ^a^µ^a-NH_2_ | 8017.2 | 8018.7 |
| ASO1_bmd | Alkyne-t^β^g^β^c^β^c^β^t^µ^t^µ^t^µ^a^µ^g^µ^g^µ^a^µ^t^µ^t^µ^c^µ^t^β^a^β^g^β^a^β^c^β^a-NH_2_ | 8340.8 | 8342.5 |
| ASO2_bmd | Alkyne-c^β^c^β^a^β^g^β^g^µ^c^µ^t^µ^g^µ^g^µ^t^µ^t^µ^a^µ^t^µ^g^µ^a^β^c^β^t^β^c^β^a^β^g-NH_2_ | 8366.7 | 8368.3 |
| ASO3_bmd | Alkyne-g^β^g^β^g^β^a^β^g^µ^t^µ^t^µ^a^µ^c^µ^t^µ^t^µ^g^µ^c^µ^c^µ^a^β^a^β^c^β^t^β^t^β^g-NH_2_ | 8382.3 | 8384.0 |
| ASO4_bmd | Alkyne-a^β^t^β^g^β^g^β^a^µ^g^µ^g^µ^t^µ^a^µ^t^µ^g^µ^a^µ^c^µ^a^µ^t^β^a^β^t^β^a^β^a^β^t-NH_2_ | 8741.6 | 8473.9 |
| scr-ASO1_bmd | Alkyne-g^β^t^β^t^β^a^β^g^µ^t^µ^g^µ^a^µ^t^µ^a^µ^c^µ^g^µ^a^µ^t^µ^g^β^a^β^t^β^a^β^a^β^a-NH_2_ | 8437.9 | 8439.5 |
| _s_: phosphorothioate group; ^µ^: mesyl (methanesulfonyl) phosphoramidates; ^β^: busyl (1-butanesulfonyl) phosphoramidates; NH_2_ – 6-aminohexyl linker; upper case: 2’-*O*-methylribonucleotide; lower case: deoxynucleotide. | | | |

**Table S2. Malat1 RNA template sequences used for the analysis of RNAse H cleavage efficiency.**

| **Code** | **Name** | **Oligonucleotide sequence, 5′-3′** |
| --- | --- | --- |
| C1 | Malat(ASO1) | AGGUCUGUCUAGAAUCCUAAAGGCAAAUGA-sCY5 |
| C2 | Malat(ASO2) | AUCUUCUGAGUCAUAACCAGCCUGGCAGUA-sCY5 |
| C3 | Malat(ASO3) | ACUUCCAAGUUGGCAAGUACUCCCAAUGA-sCY5 |
| C4 | Malat(ASO4) | AAAAGAUUAUAUGUCAUACCUCCAUUGGGG-sCY5 |
|  | Malat(scr) | AAUUAUUUUAUCAUCGUAUCACUAACCUAAC-sCY5 |


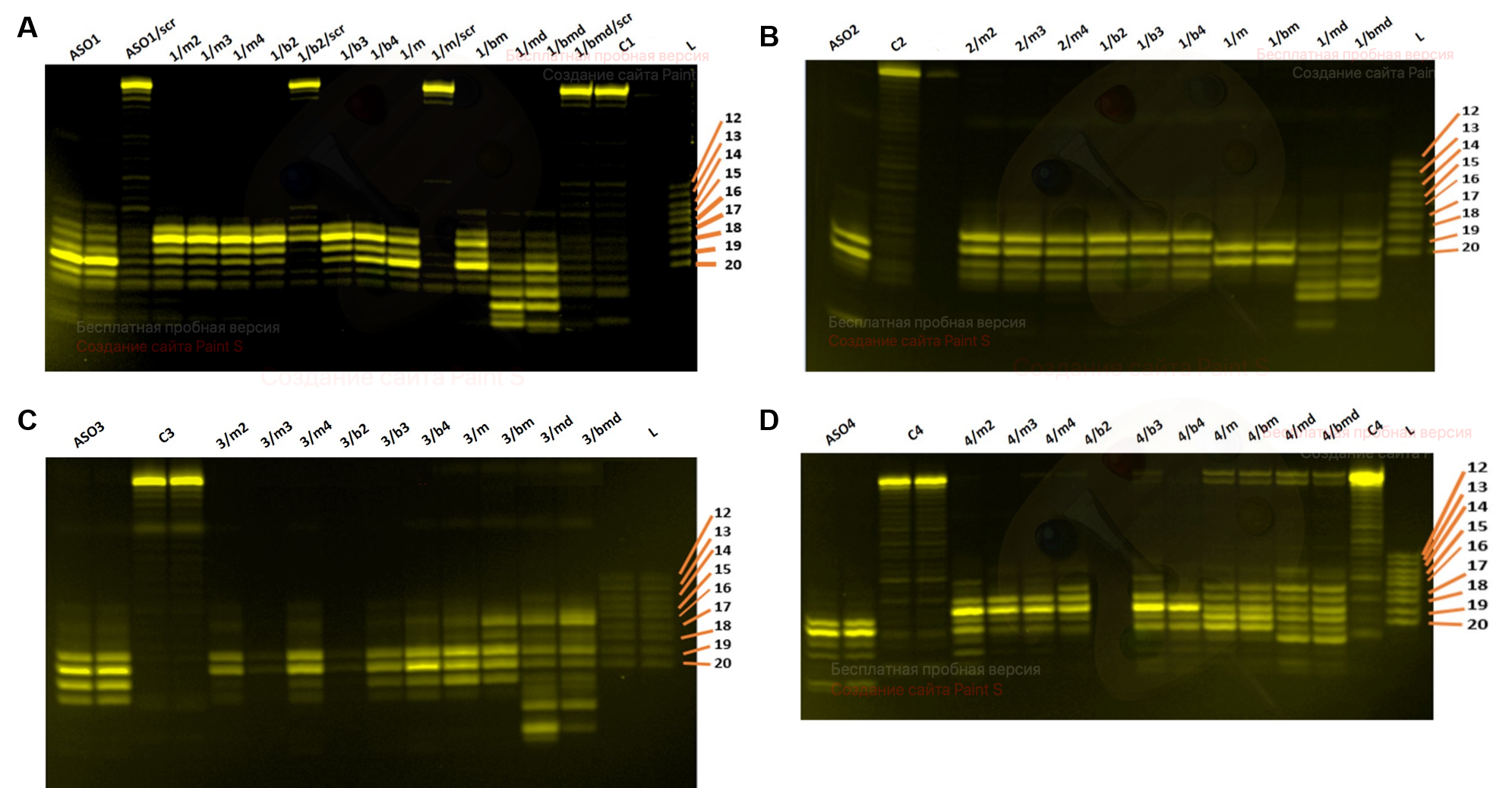


**Figure S1.** RNAse H cleavage of Malat1 RNA templates complexed with modified ASOs. ASO1-4: phosphorothioate; m2, m3, m4: mesyl-modified ASOs; b2, b3, b4: busyl-modified ASOs; m: all-mesyl-ASO; scr: control scrambled ASO; bm: ASO containing mesyl in the gap and busyl in the wings; C1-4: control RNA templates (Table S2); L: ladder of Cy5-labelled fragments of Malat1 template with defined lengths.


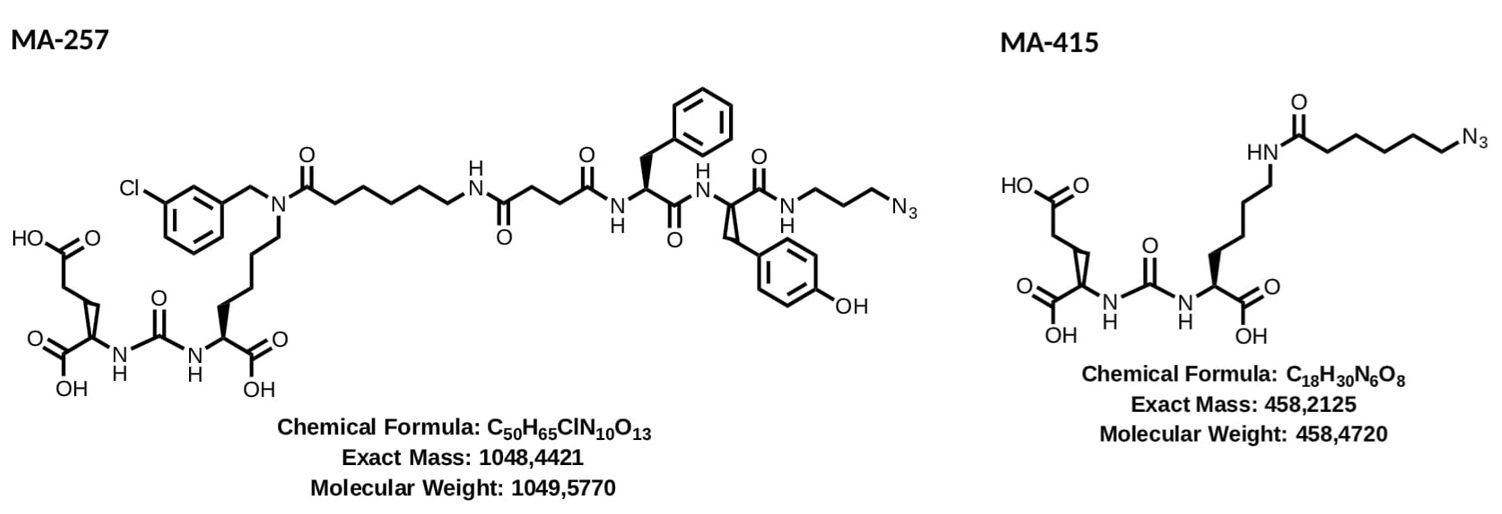


**Figure S2**. Structures and chemical formulae of the two validated PSMA ligands MA-257 and MA-415 used for the conjugation with antisense oligonucleotides *via* the corresponding azido derivatives.


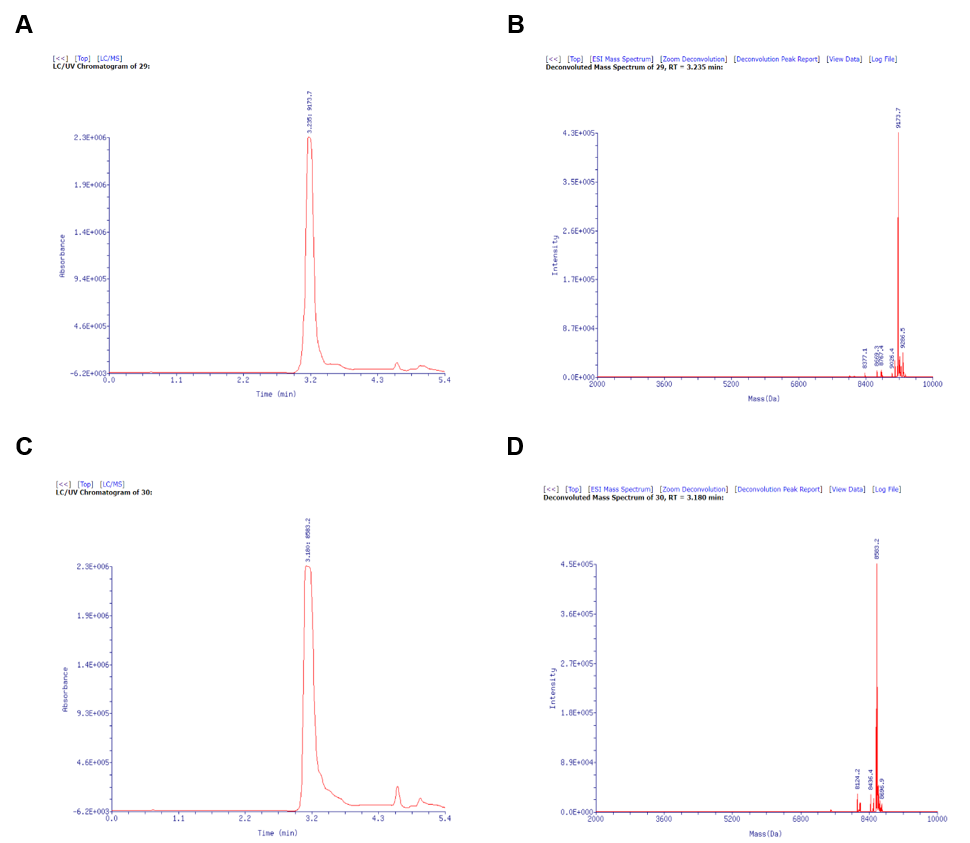


**Figure S3**. LC-UV traces (**A**, **C**) and ESI MS spectra (**B**, **D**) of the PSMA-oligonucleotide conjugates 257-ASO2 md (**A**, **B**) and 415-ASO2 md (**C**, **D**) (see Fig. 3 for the linker structures).


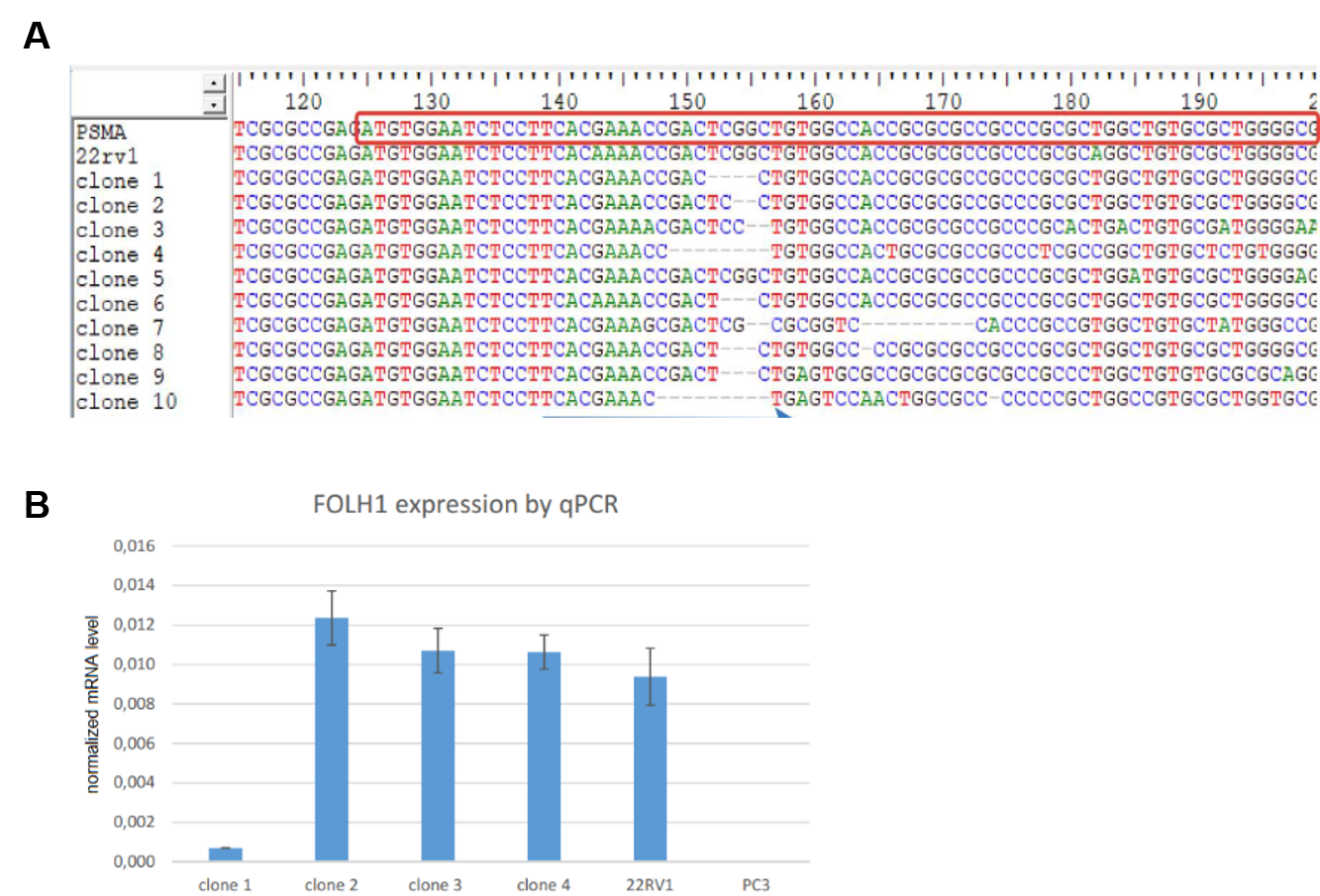


**Figure S4.** Validation of the PSMA–/– cell line generation using CRISPR/Cas approach. **A**. Sanger sequencing of the selected PSMA–/– cell clones. **B**. Validation of PSMA (FOLH1) depletion by RT-qPCR with normalization on the GAPDH mRNA level.
