# Supplementary material for: Structure-activity relationship study of mesyl and busyl phosphoramidate antisense oligonucleotides for unaided and PSMA-mediated uptake into prostate cancer cells
Source: Front Chem. 2024 Mar 4;12:1342178. doi: 10.3389/fchem.2024.1342178 (PMC10944894; doi:10.3389/fchem.2024.1342178)
Supplement: Supplementary file 2 [file DataSheet2.docx]

Supplementary Material Part 2

Structure-activity relationship study of mesyl and busyl phosphoramidate antisense oligonucleotides for unaided and PSMA-mediated uptake into prostate cancer cells

***Oligonucleotide ESI LC-MS profiles***

**ASO1/m2**


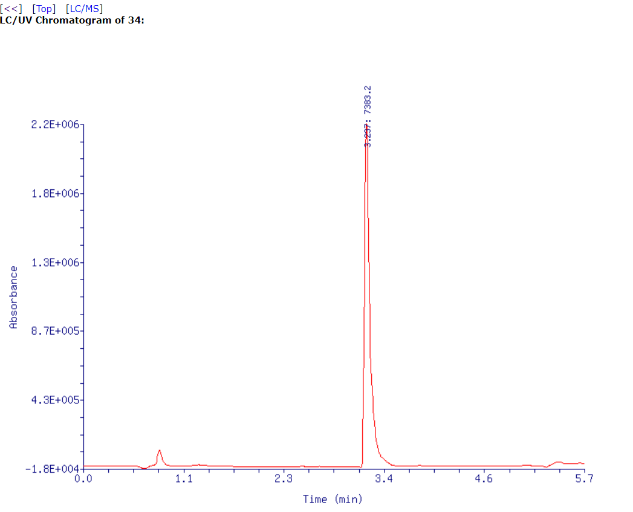

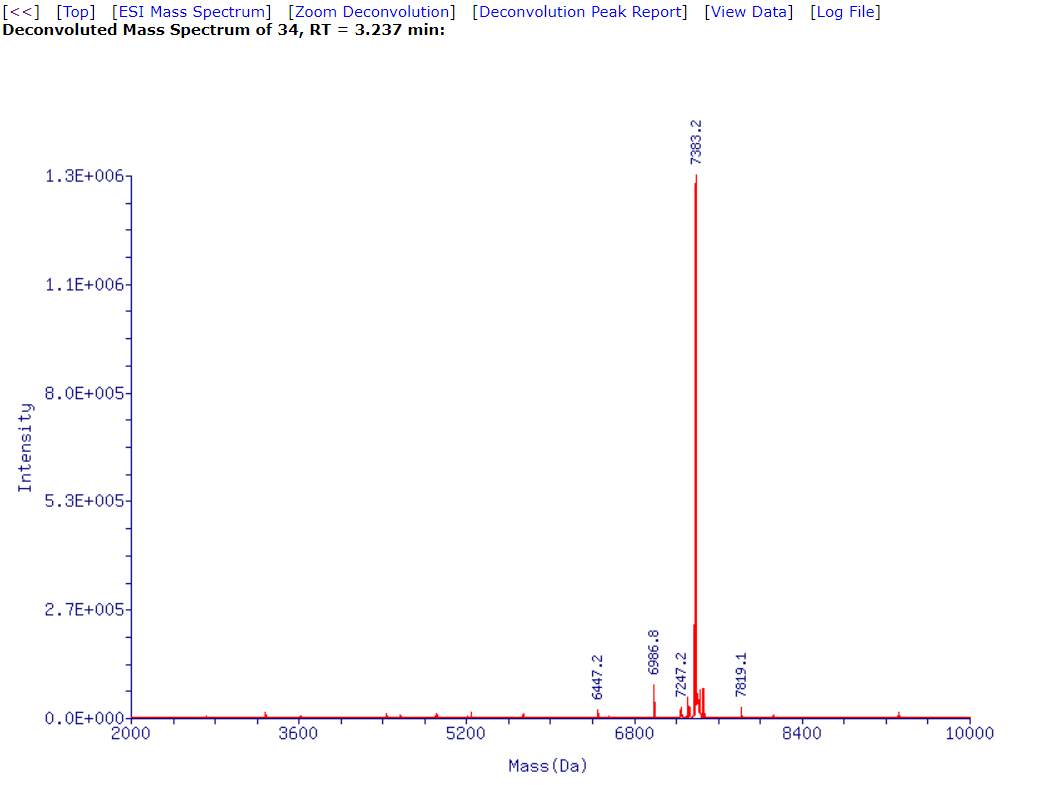


**ASO2/m2**


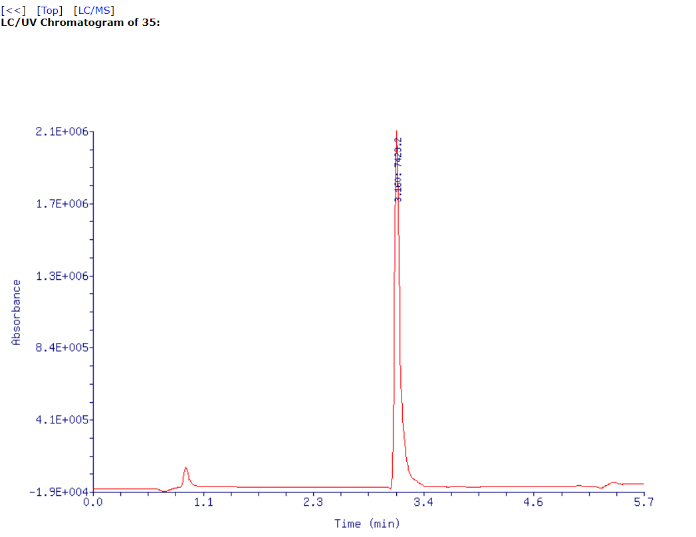

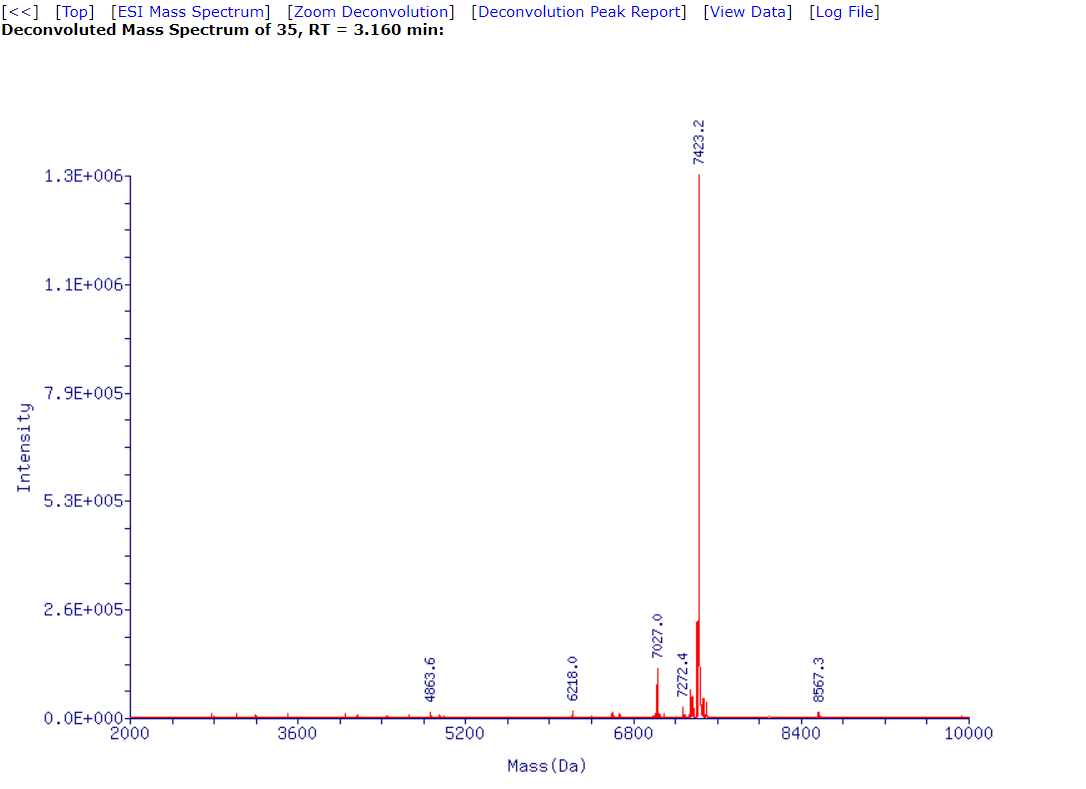


**ASO3/m2**


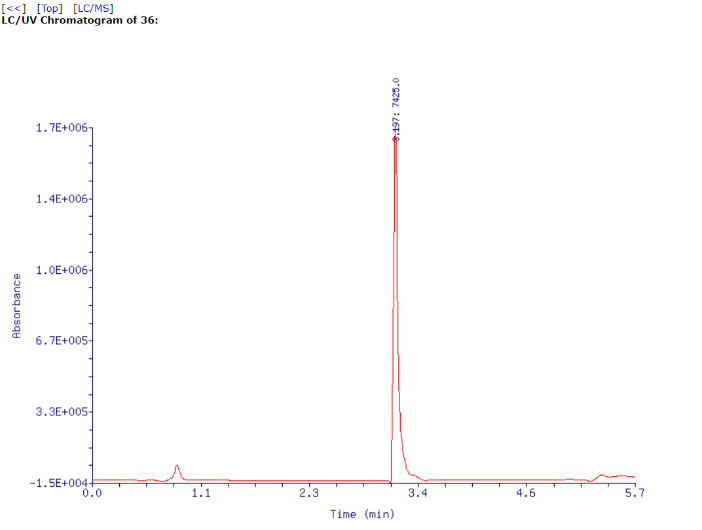

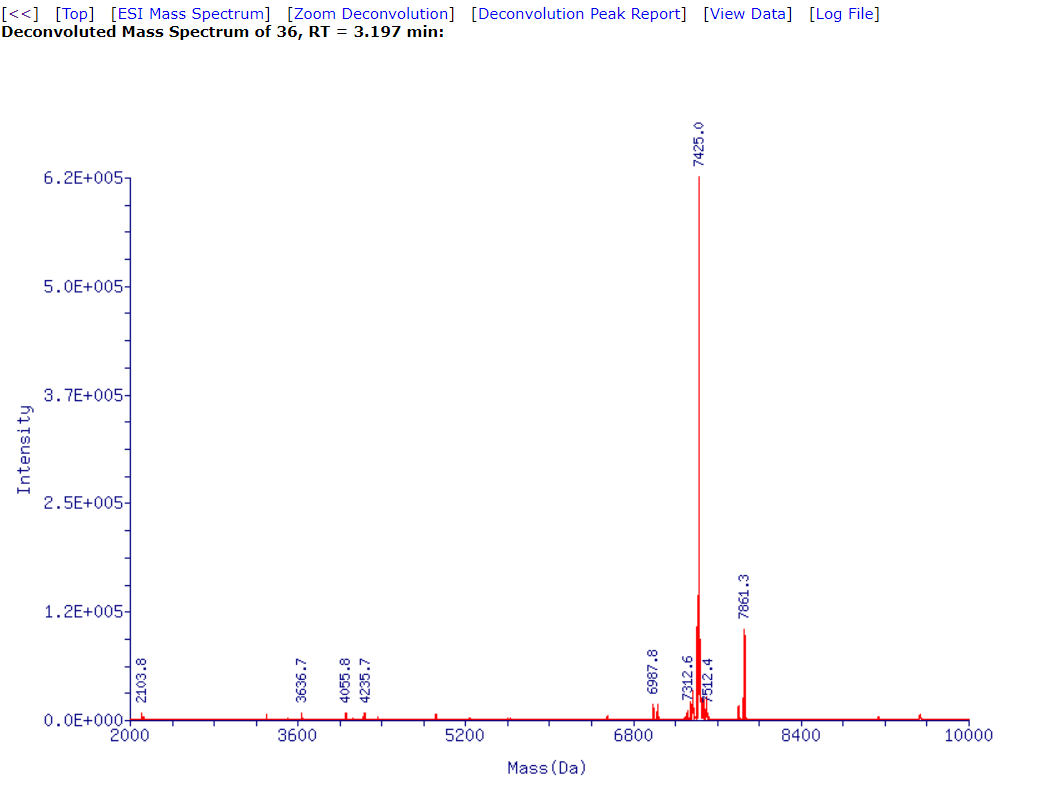


**ASO4/m2**


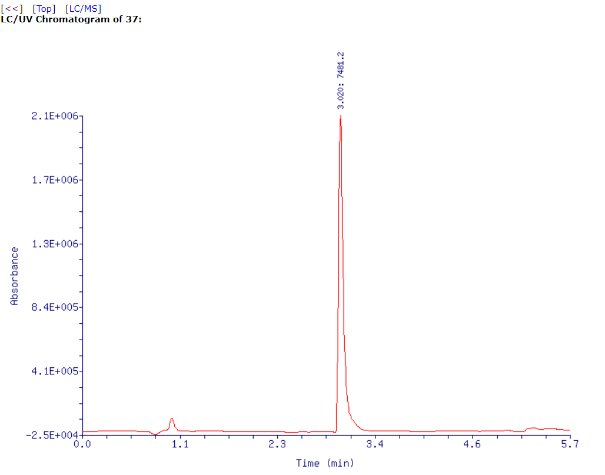

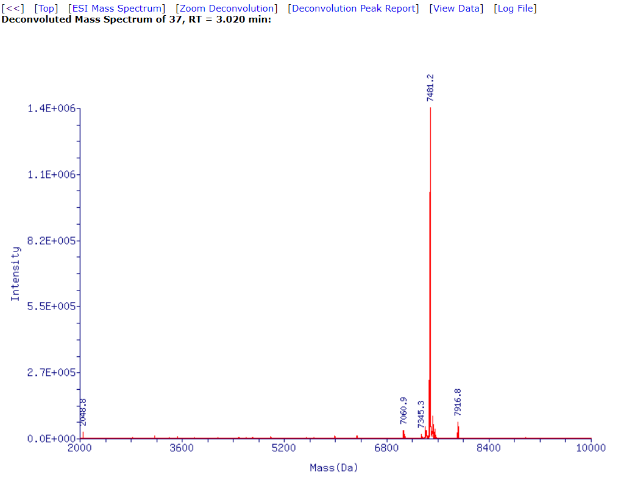


**ASO1/m2-sCy5**


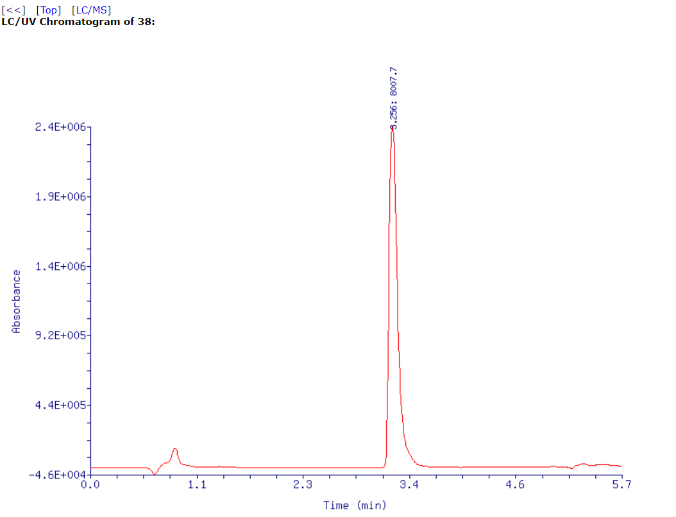

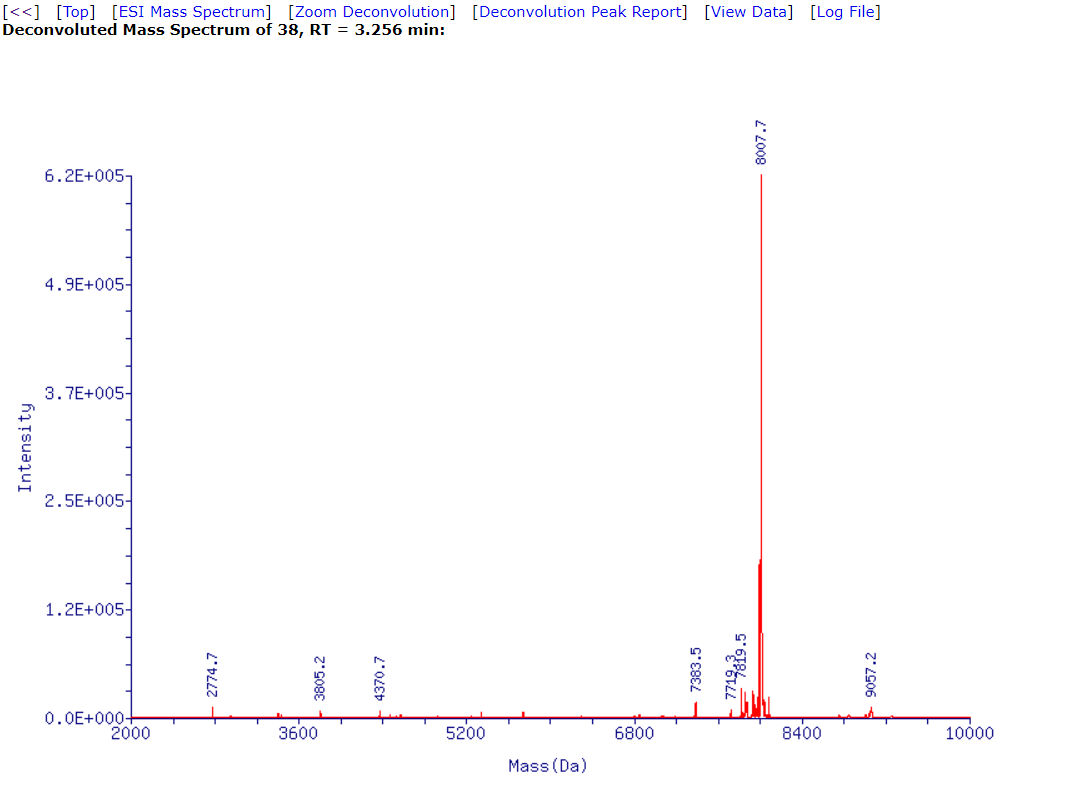


**ASO2/m2-sCy5**


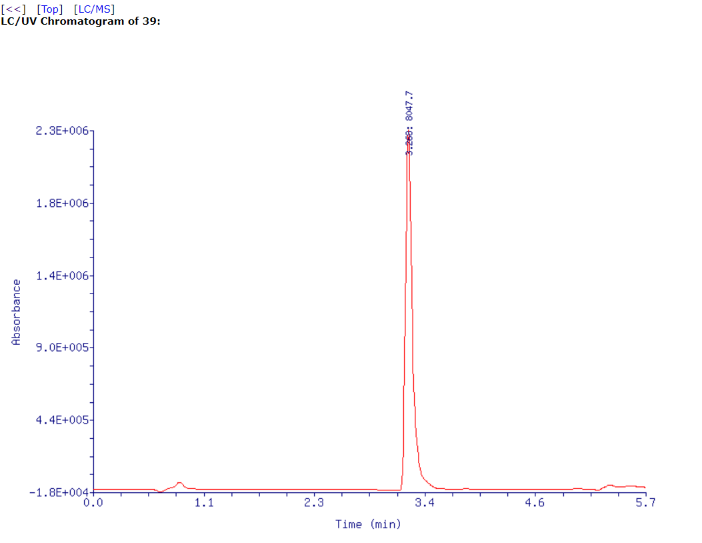

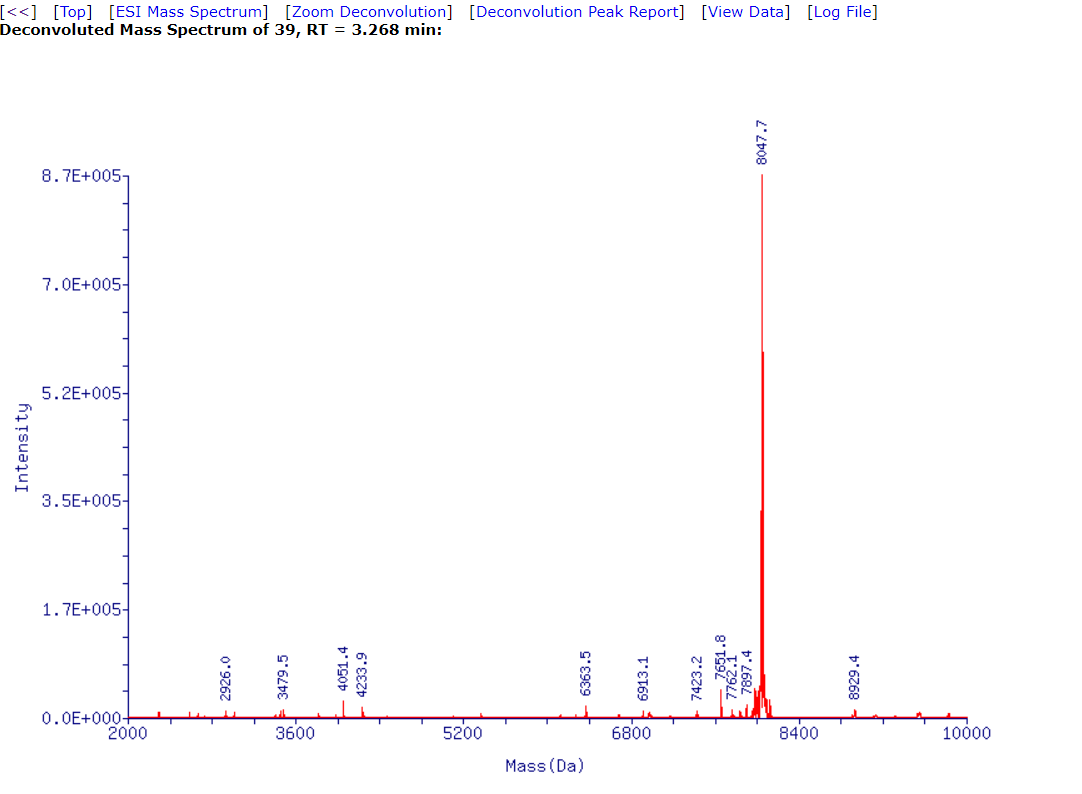


**ASO4/m2-sCy5**


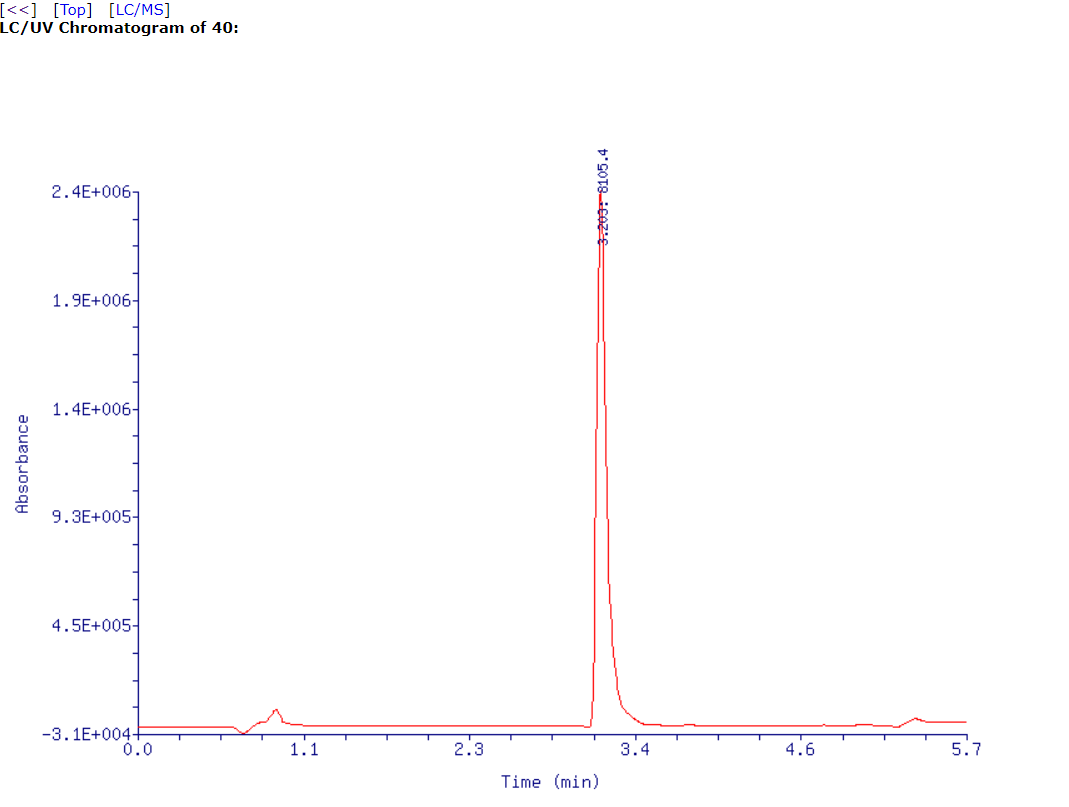

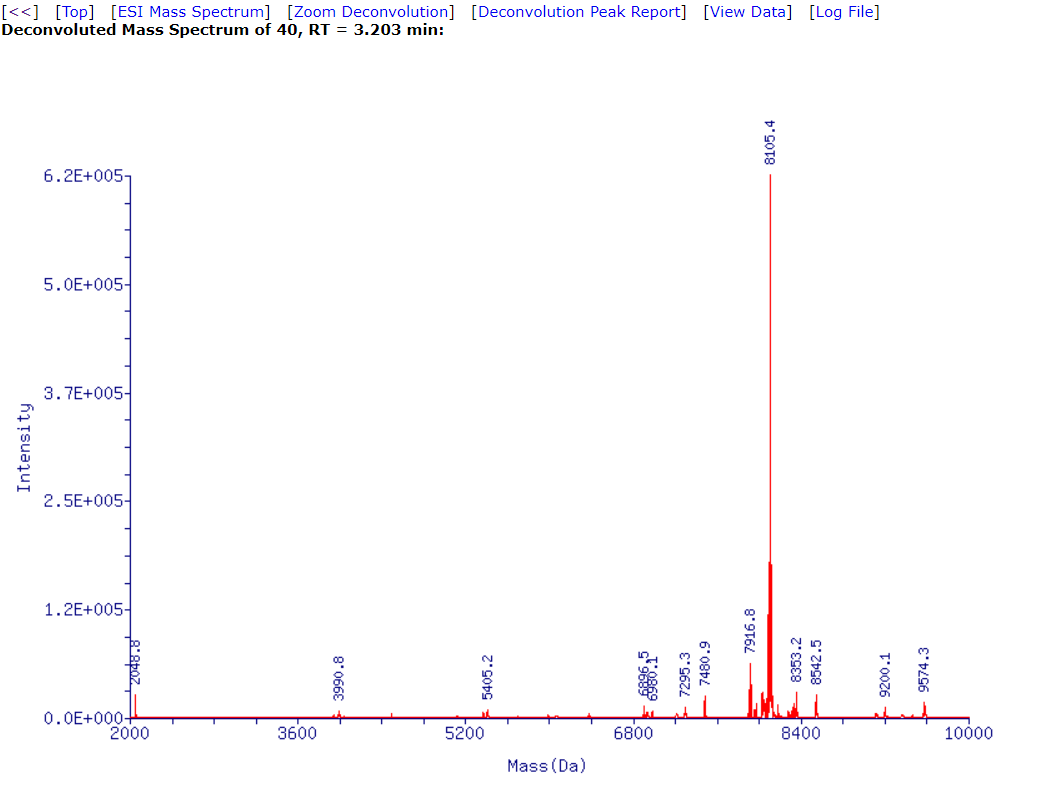


**scr-ASO1/m2**


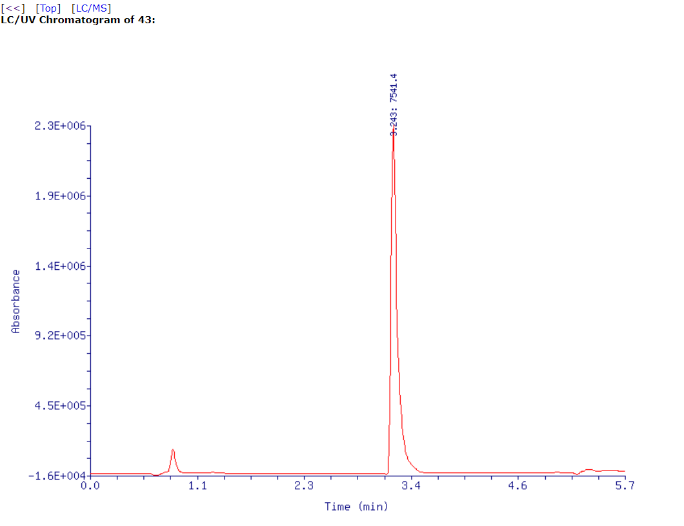

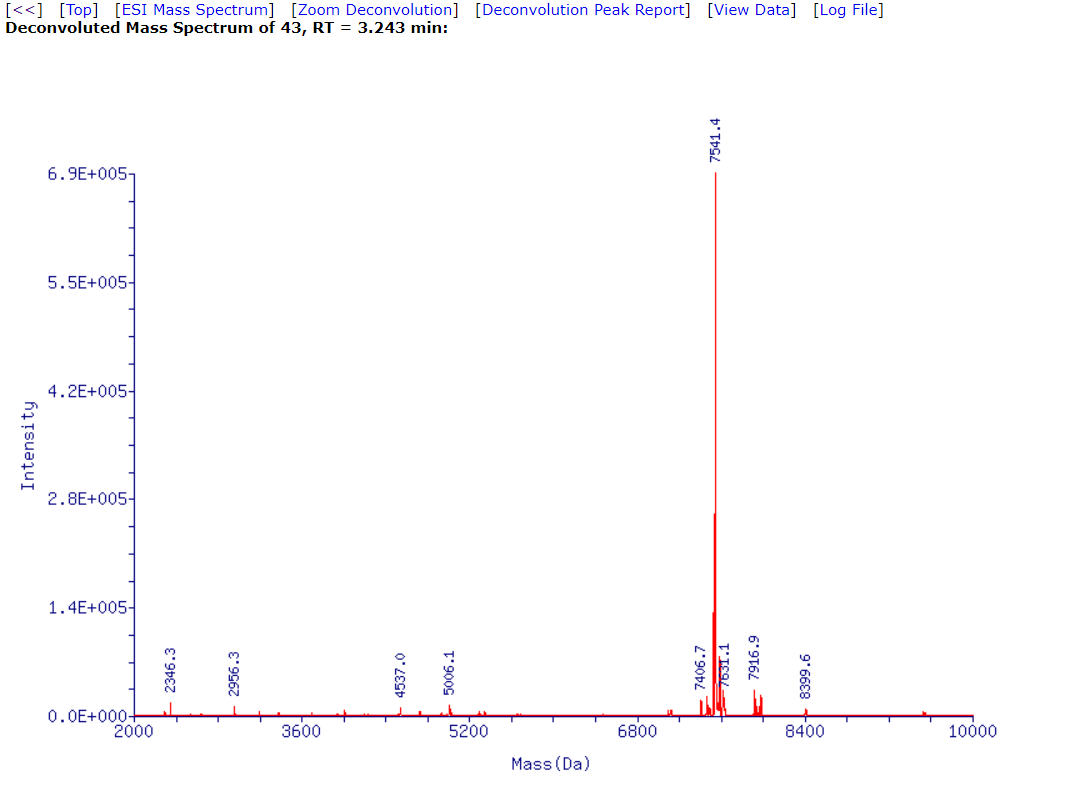


**scr-ASO1/m2-sCy5**


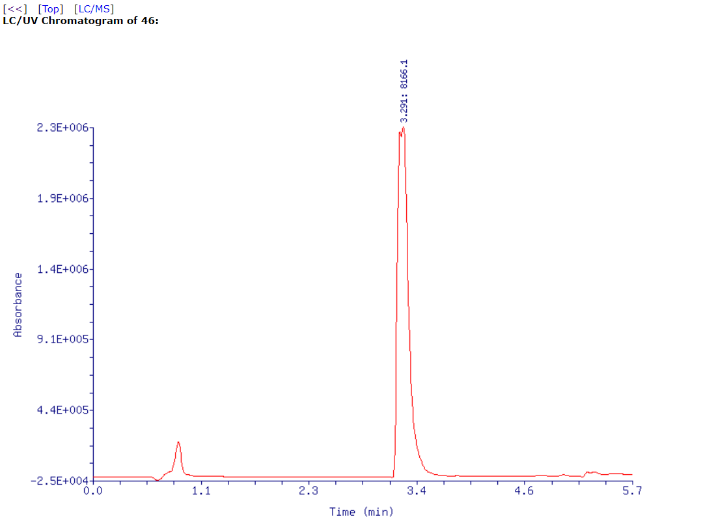

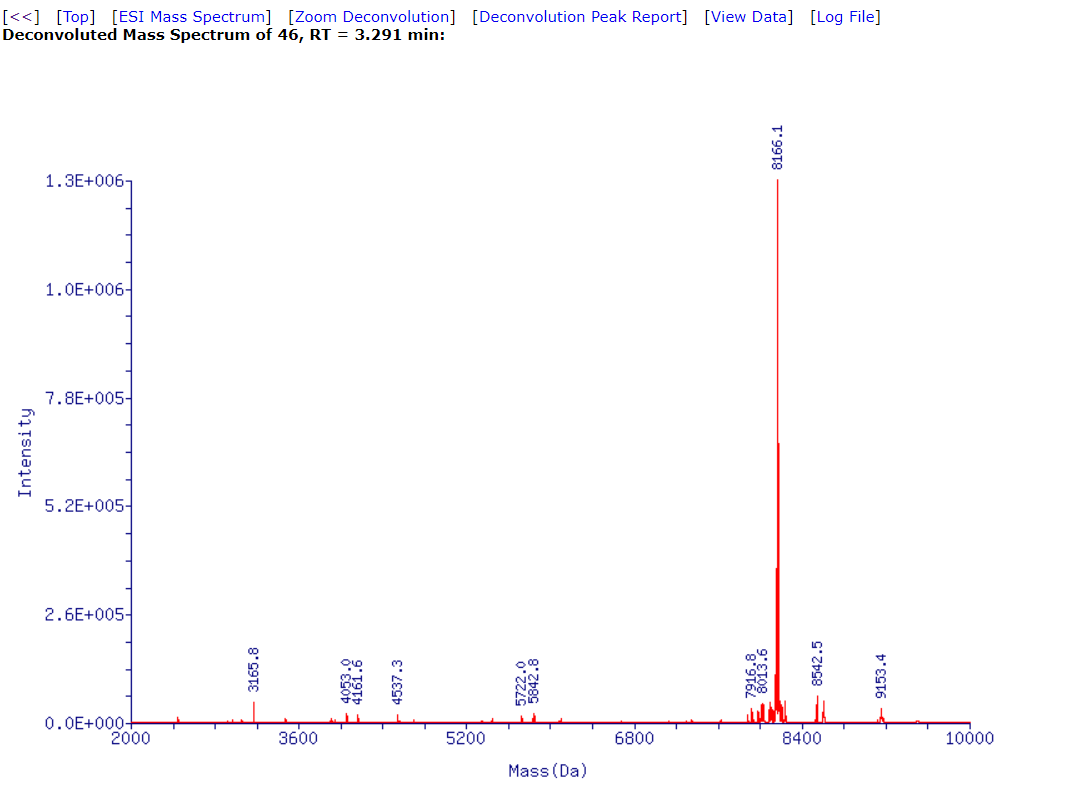


**ASO1/m3**


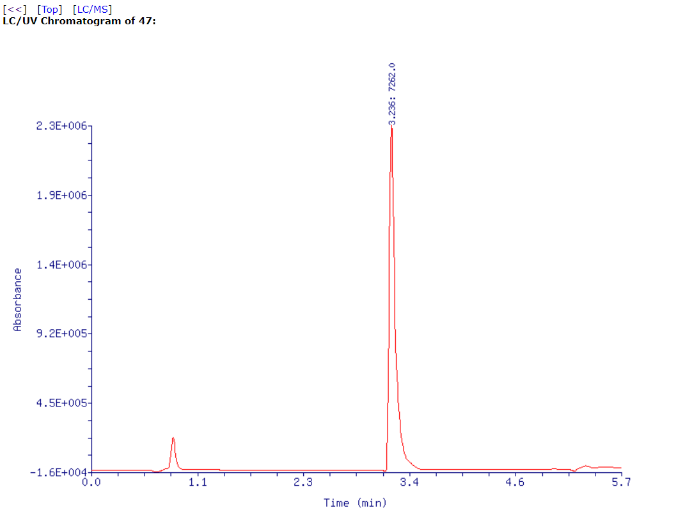

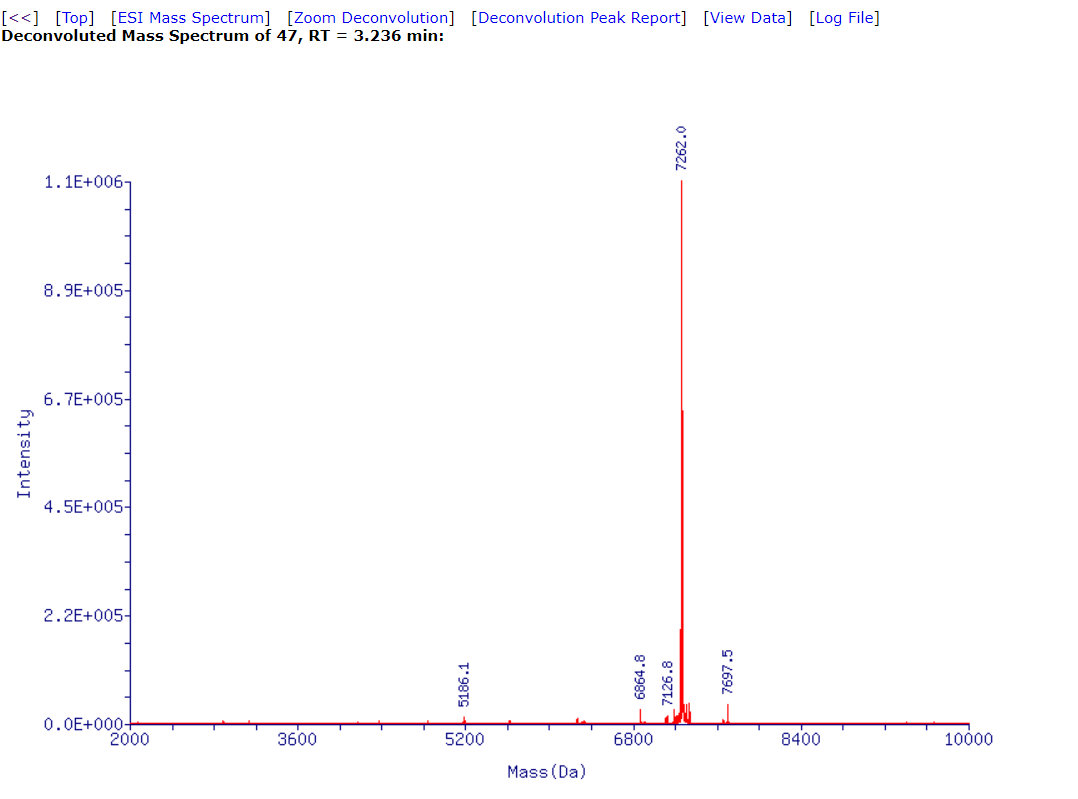


**ASO2/m3**


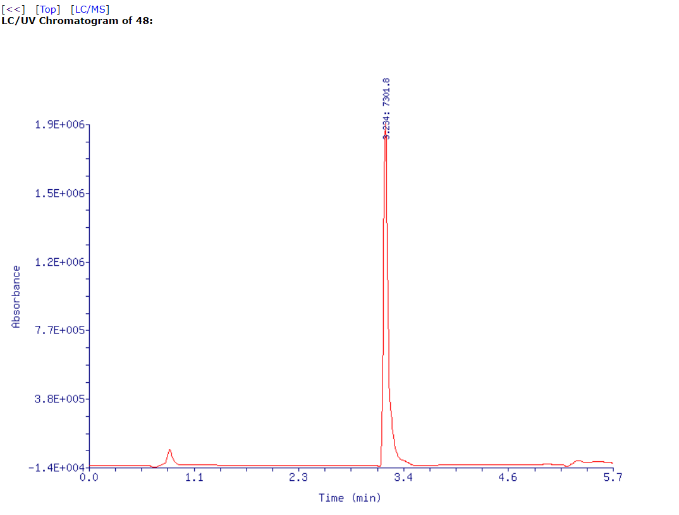

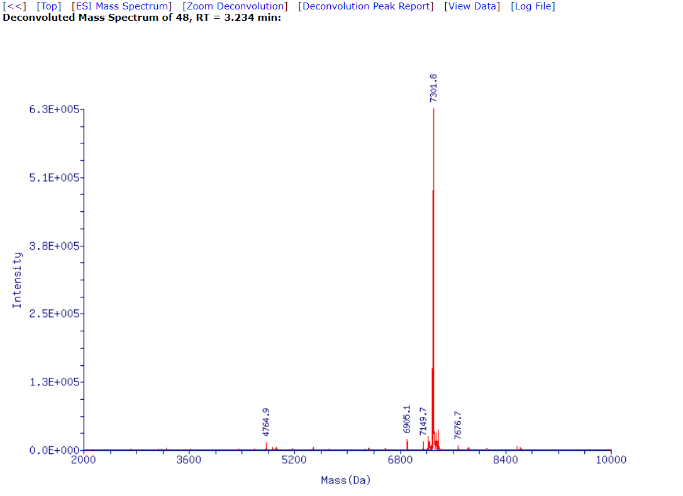


**ASO3/m3**


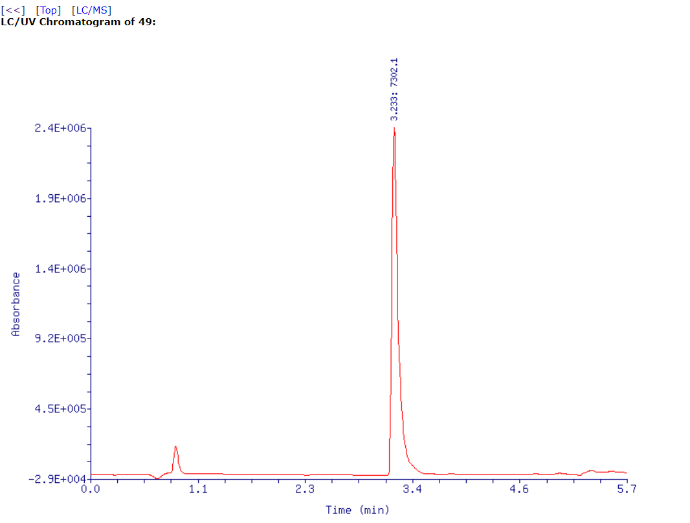

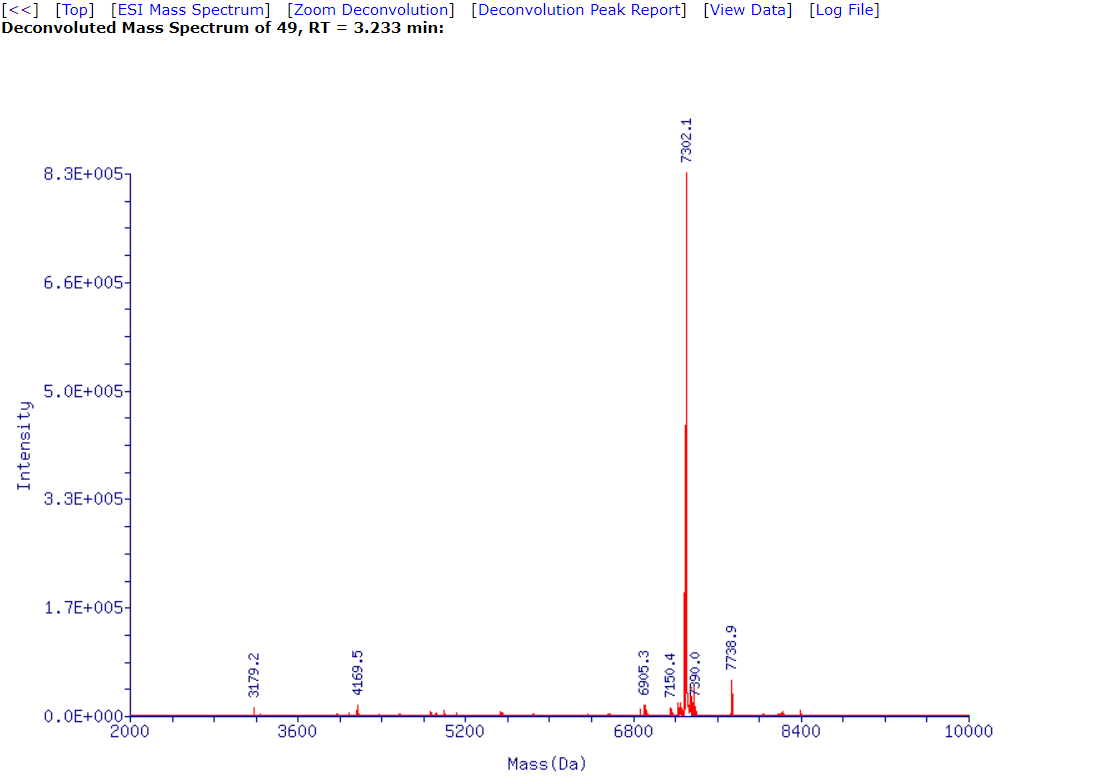


**ASO4/m3**


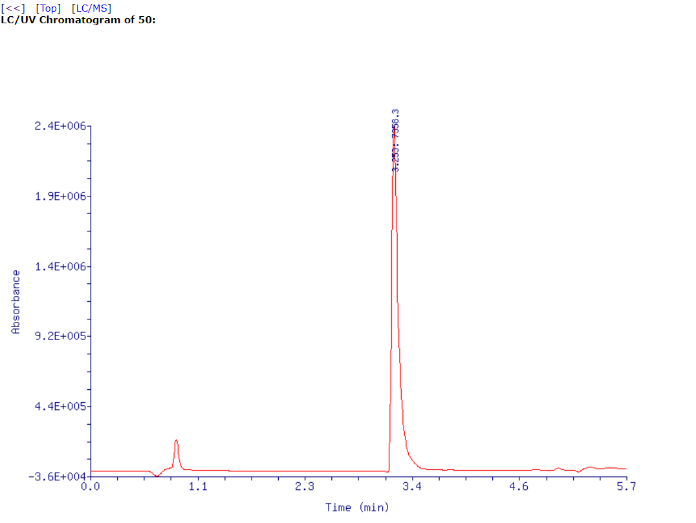

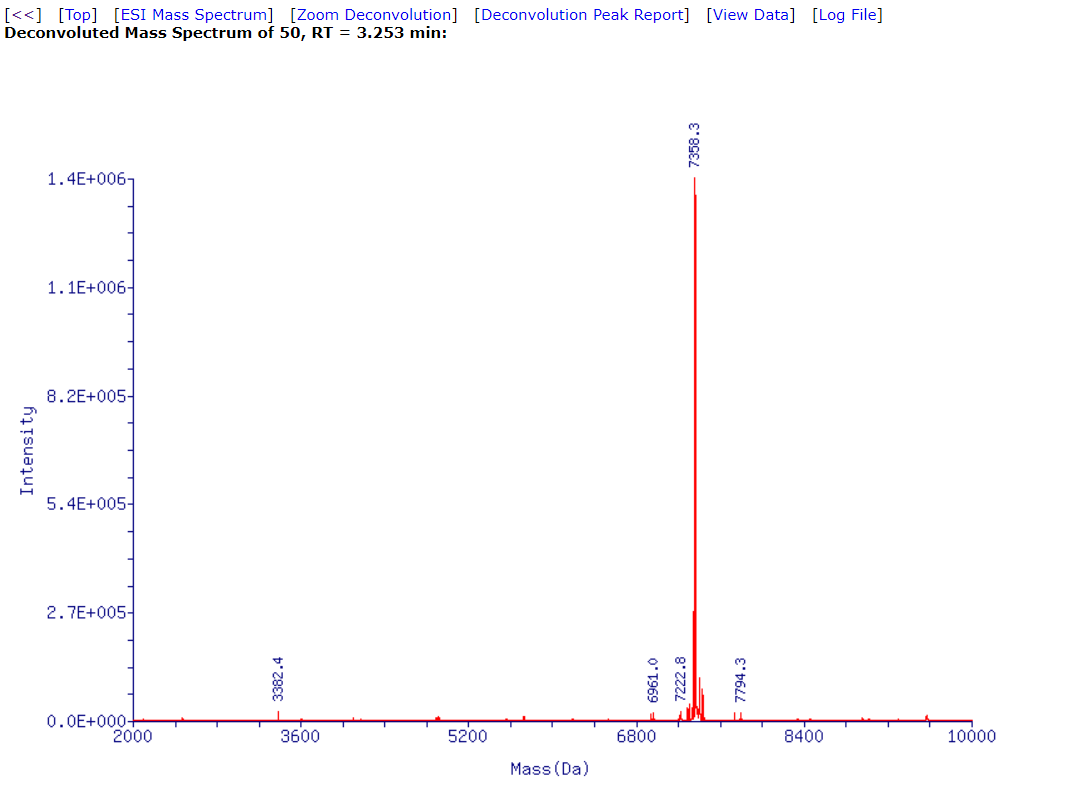


**ASO1/m3-sCy5**


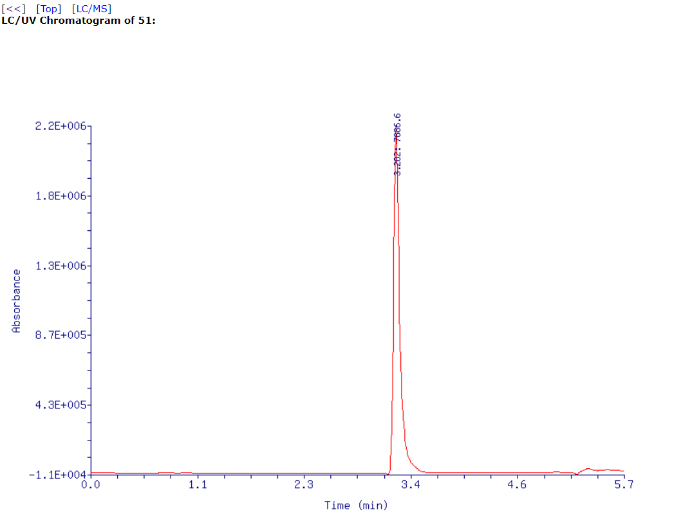

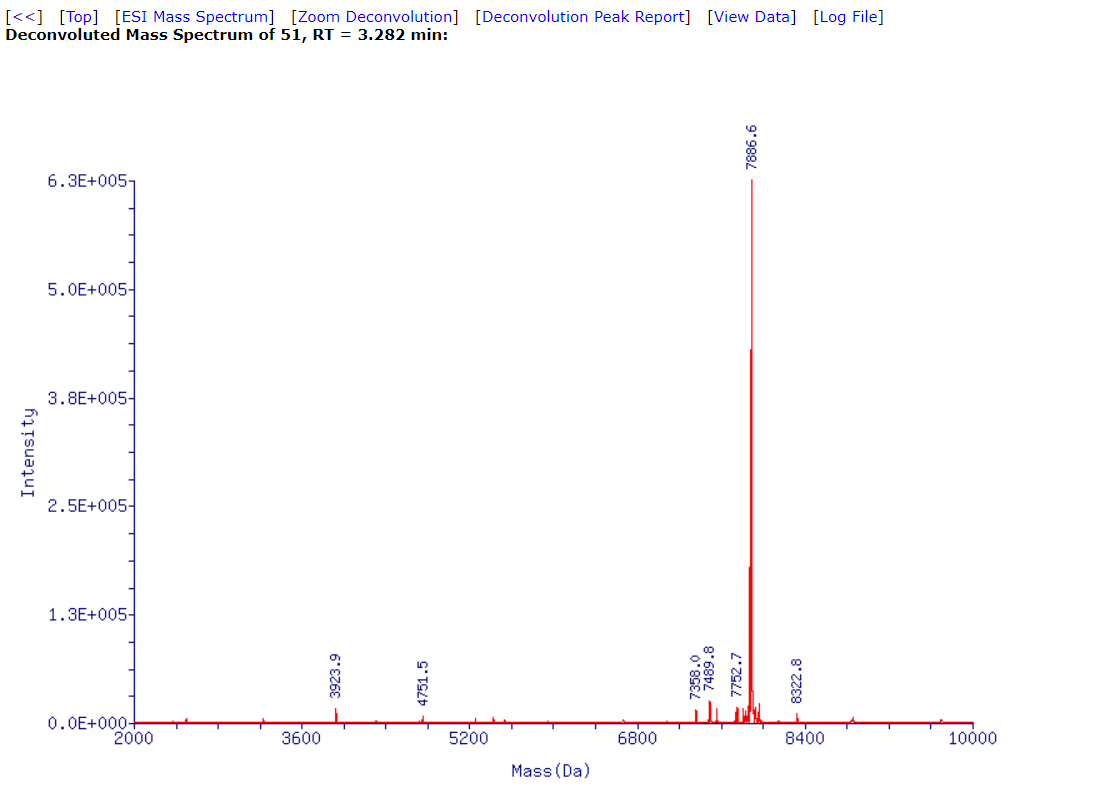


**ASO2/m3-sCy5**


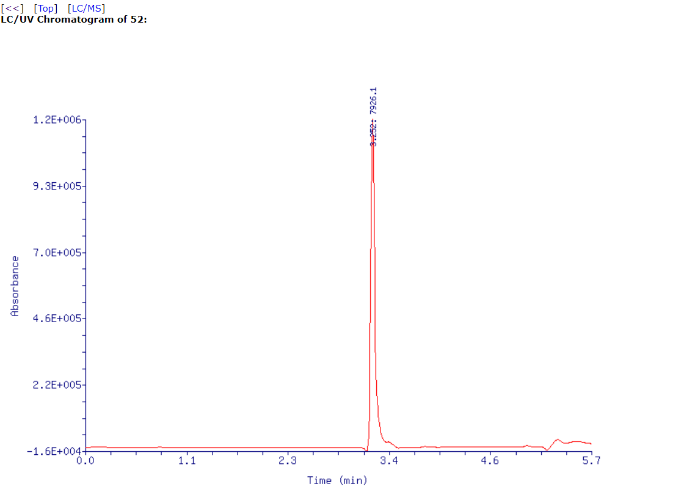

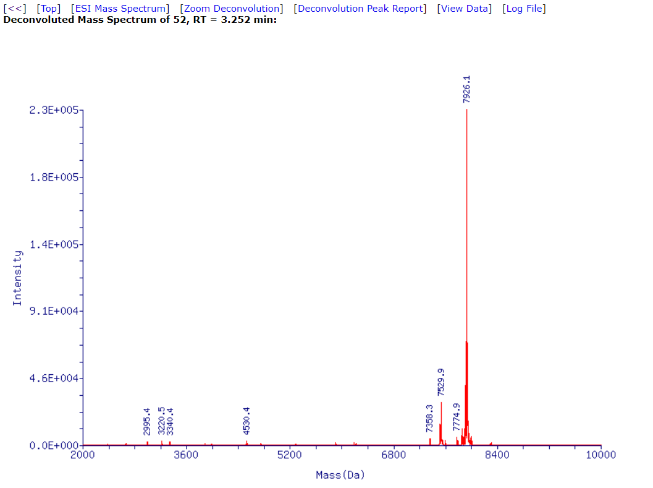


**ASO3/m3-sCy5**


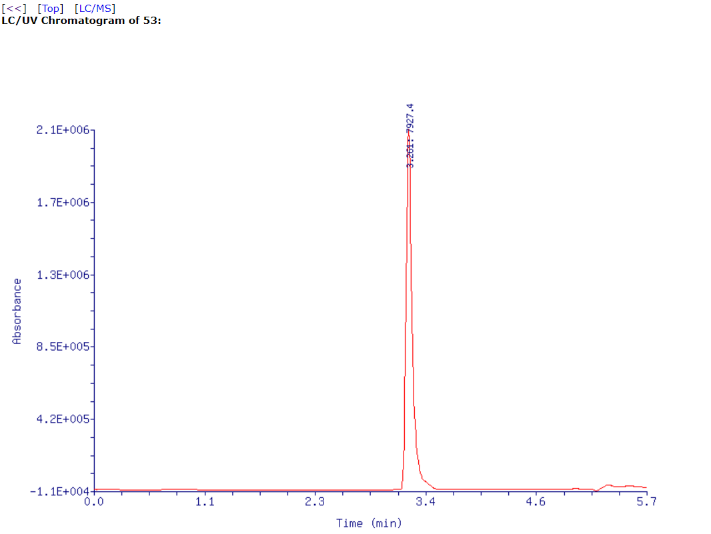

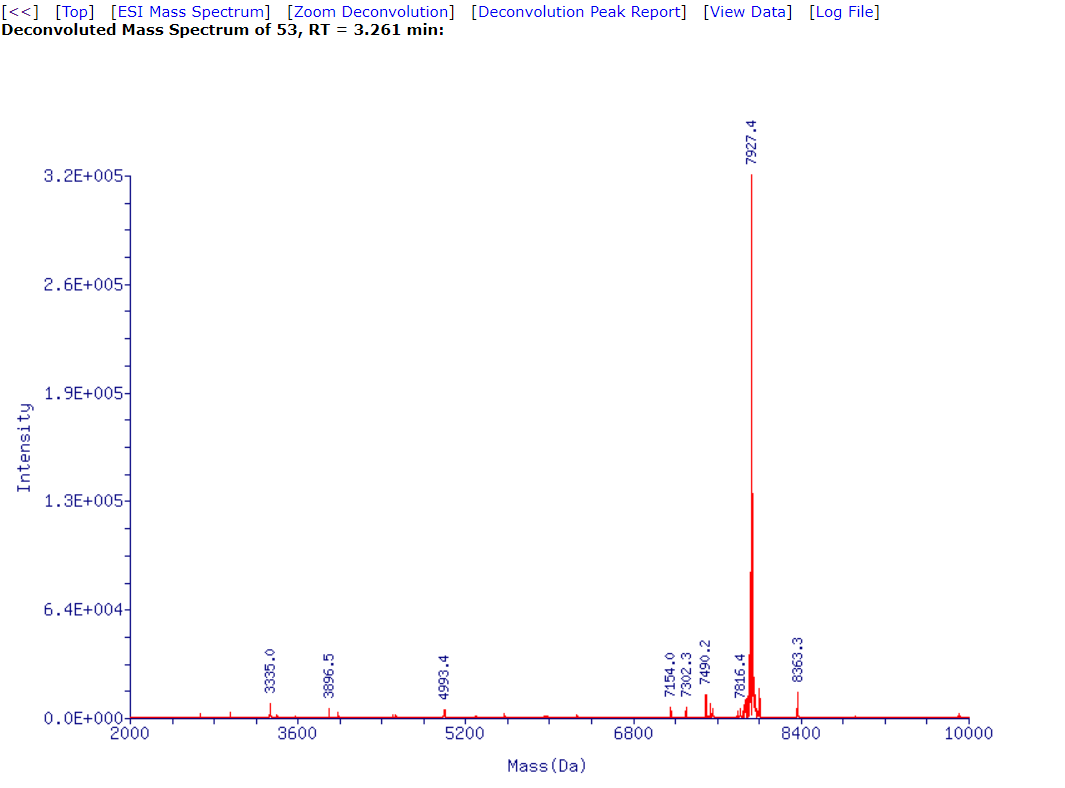


**ASO4/m3-sCy5**


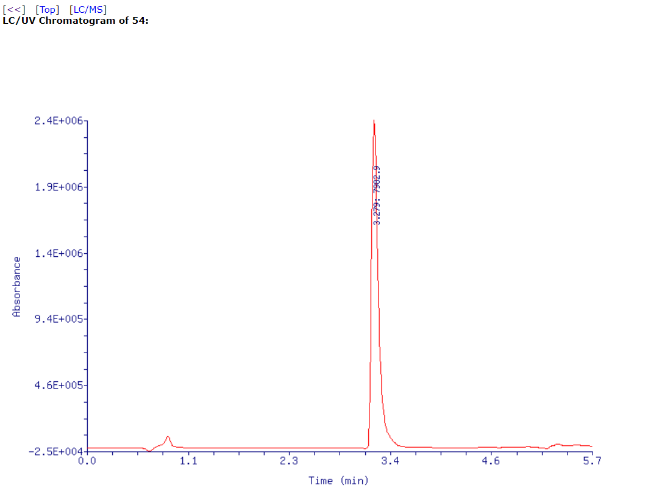

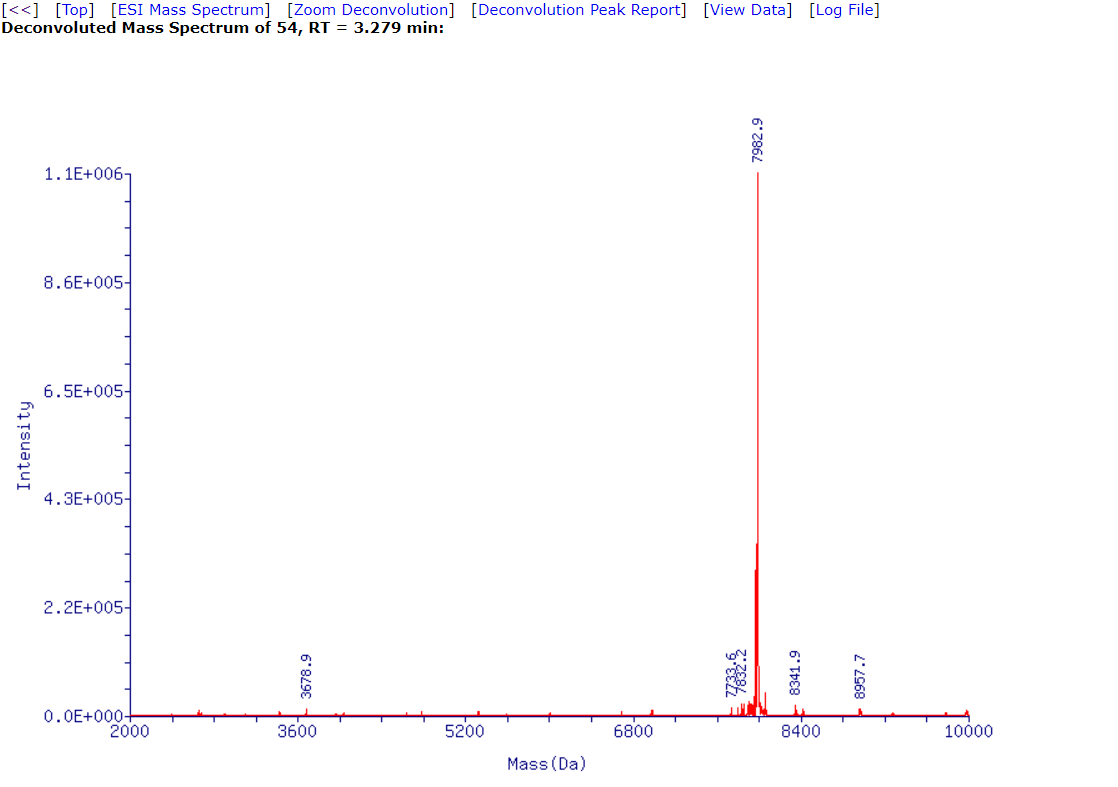


**scr-ASO1/m3**


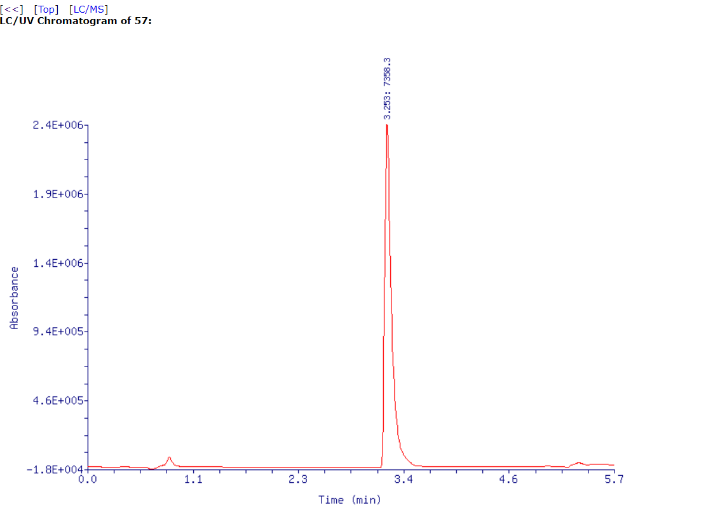

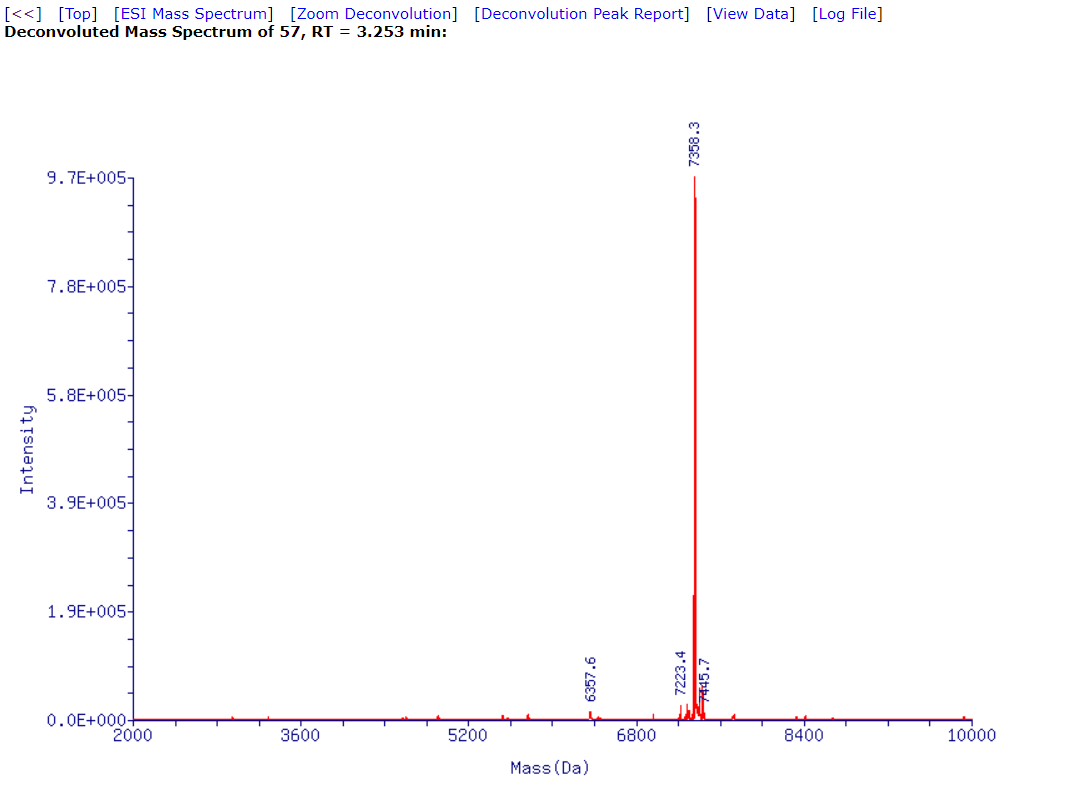


**scr-ASO1/m3-sCy5**


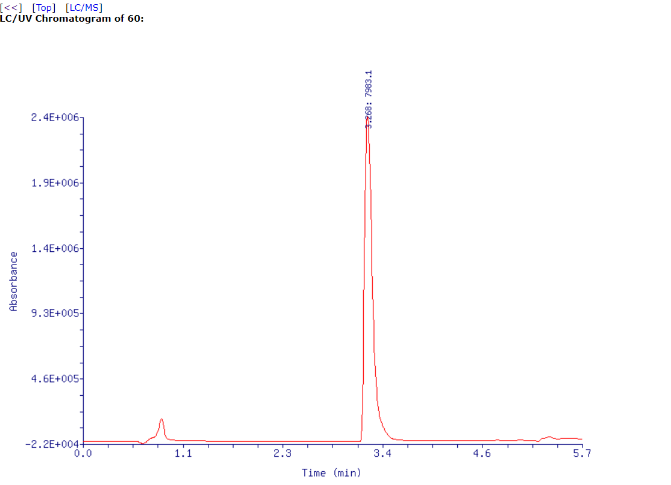

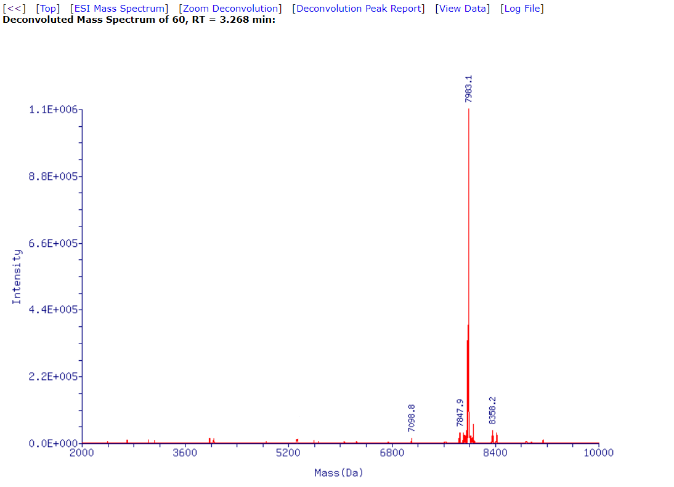


**ASO1/m4**


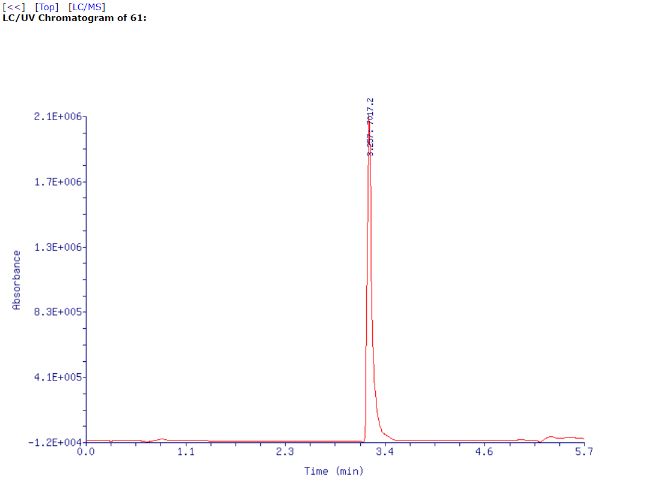

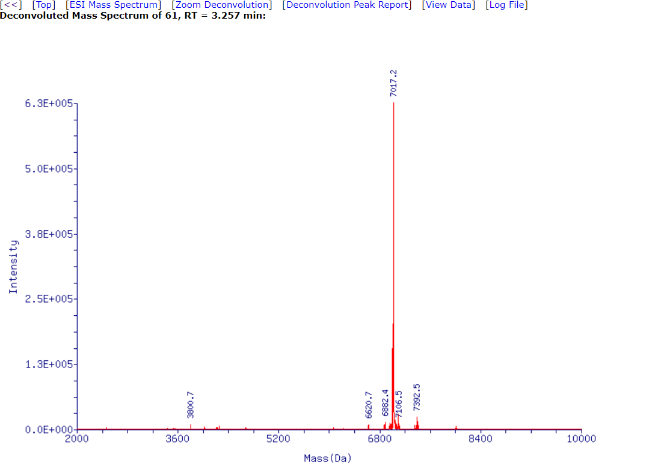


**ASO2/m4**


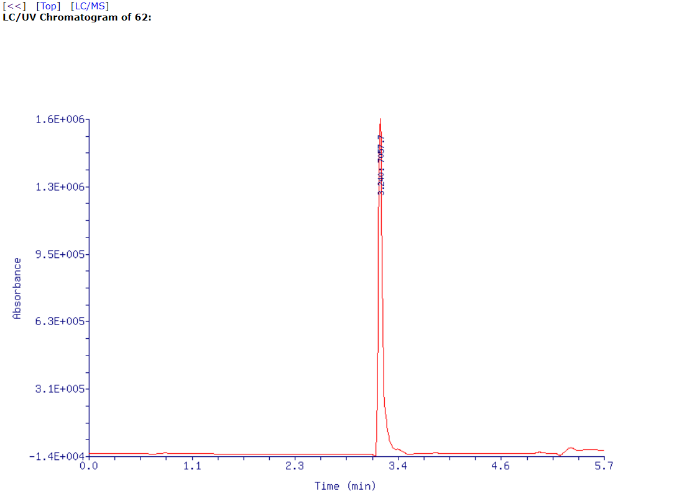

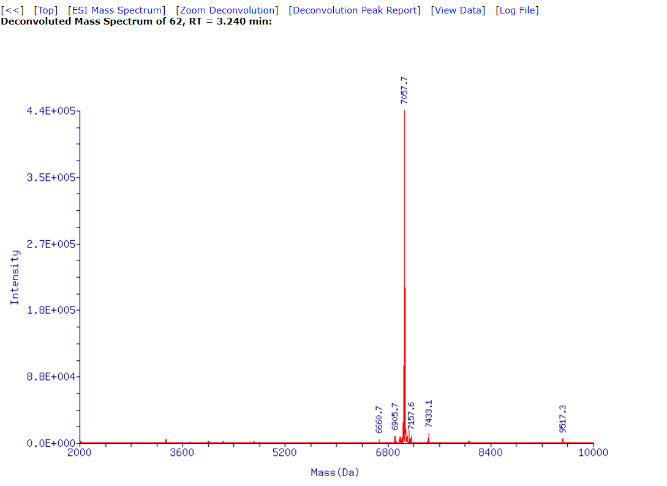


**ASO3/m4**


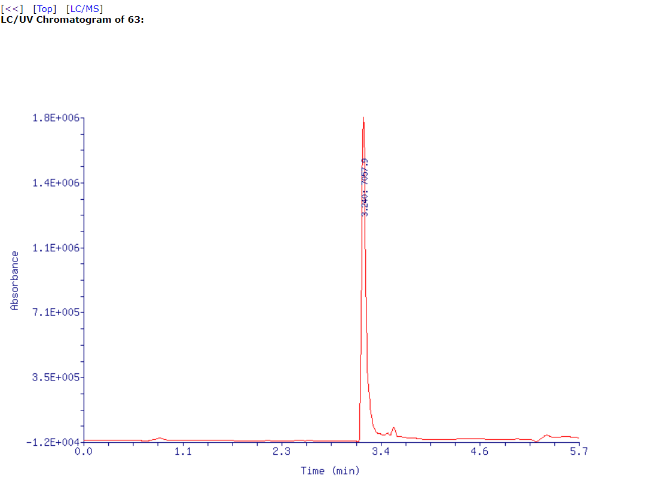

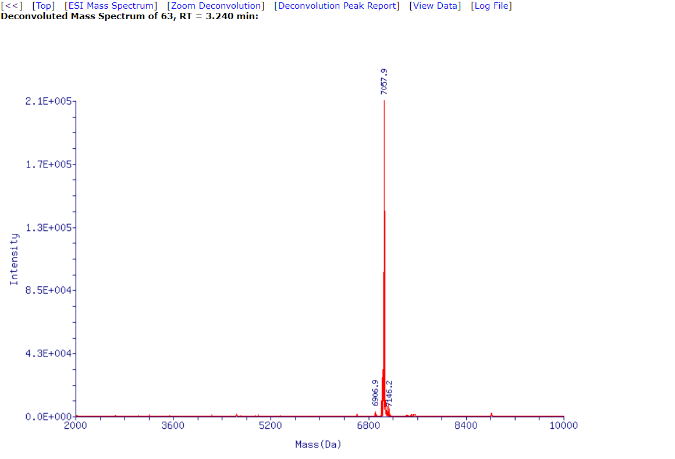


**ASO4/m4**


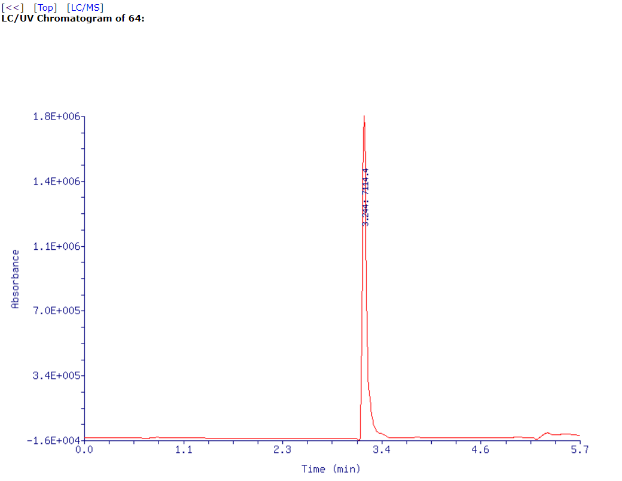

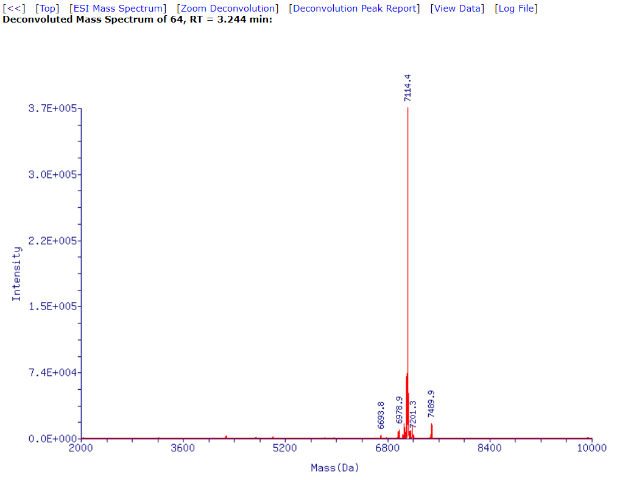


**ASO1/m4-sCy5**


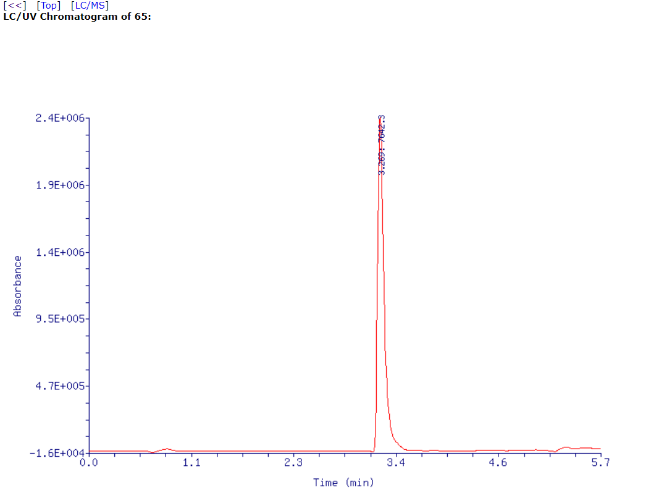

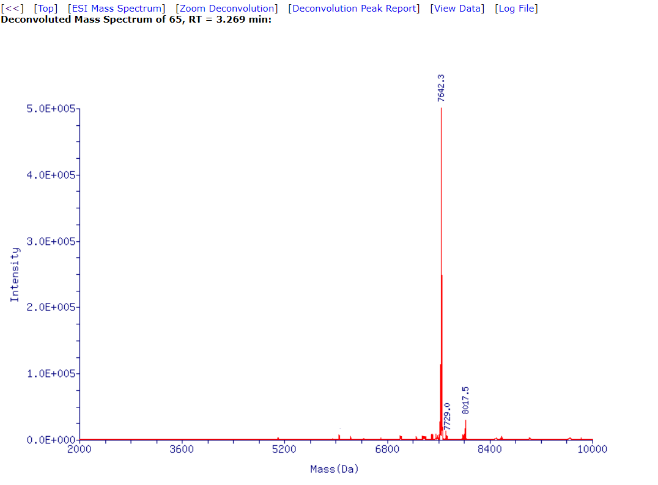


**scr-ASO1/m4**


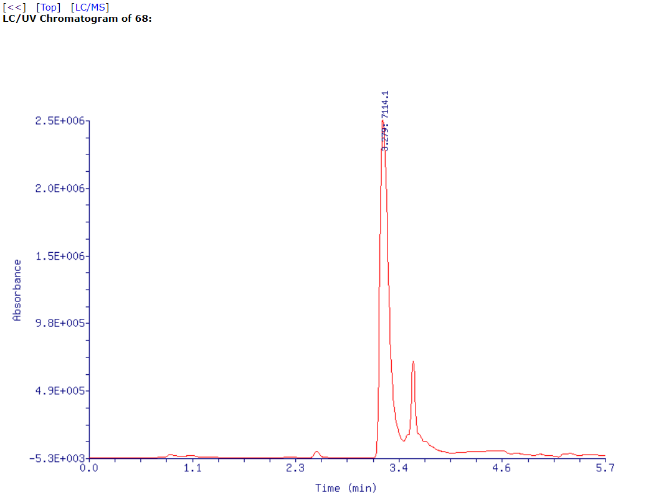

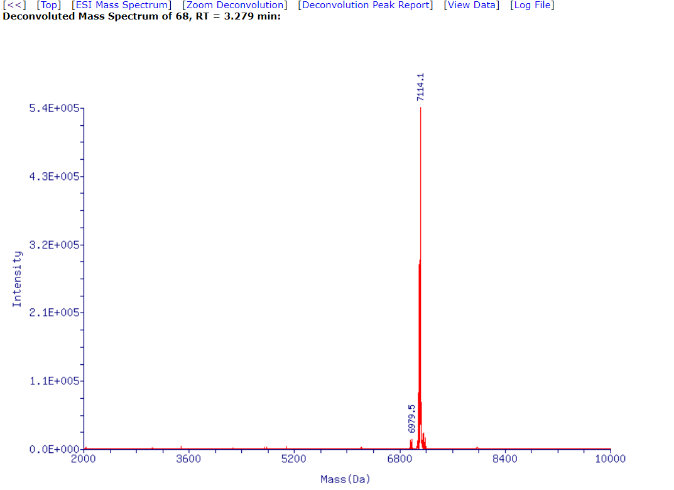


**scr-ASO1/m4-sCy5**


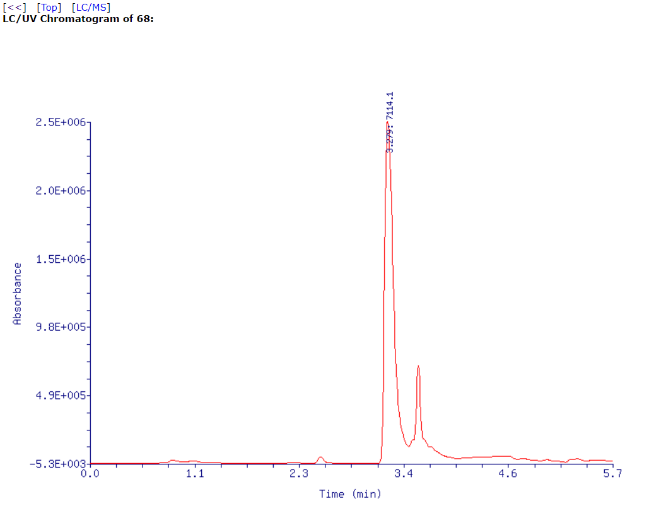

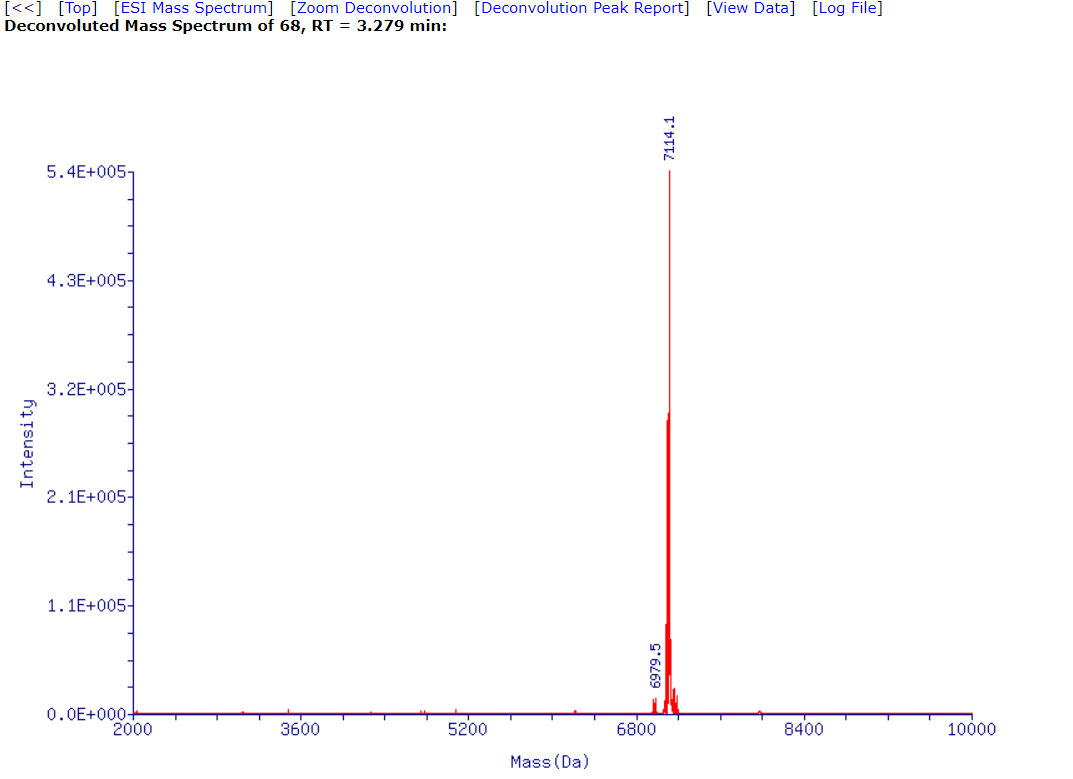


**ASO1/b2**


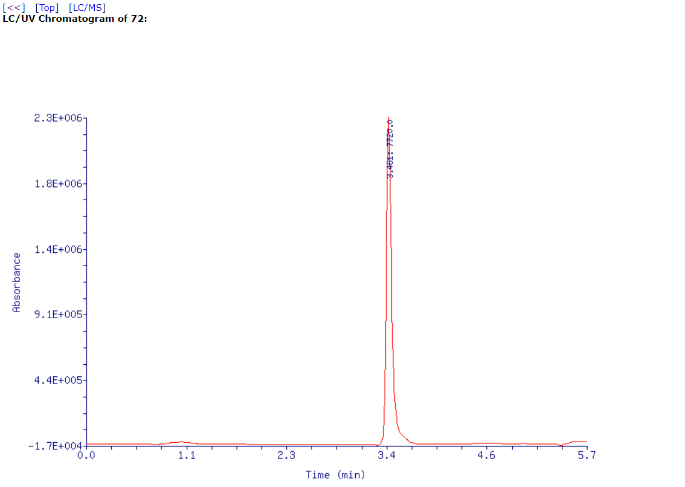

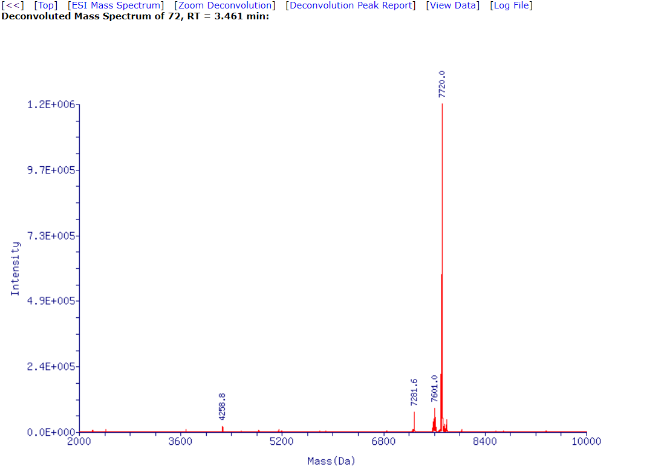


**ASO2/b2**


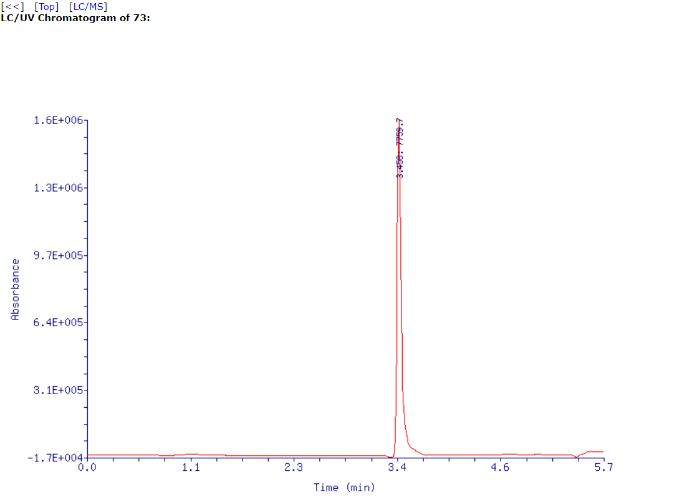

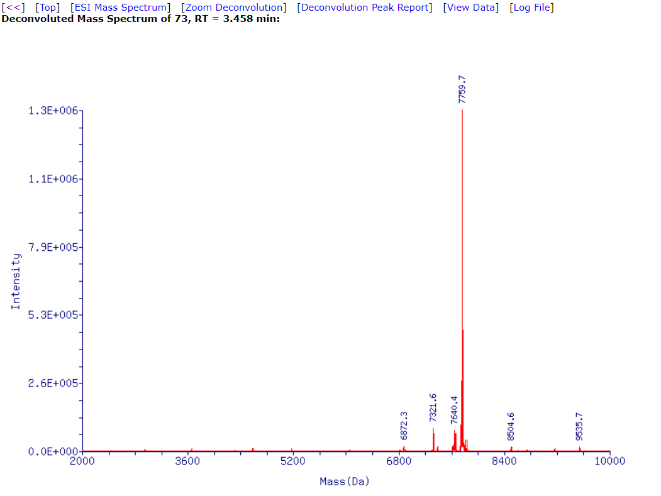


**ASO3/b2**


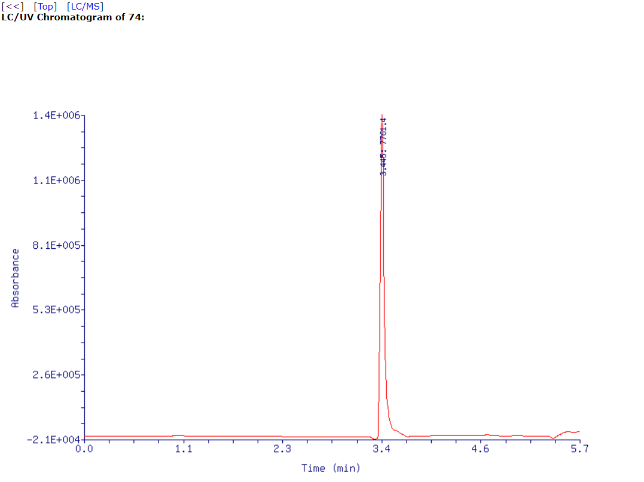

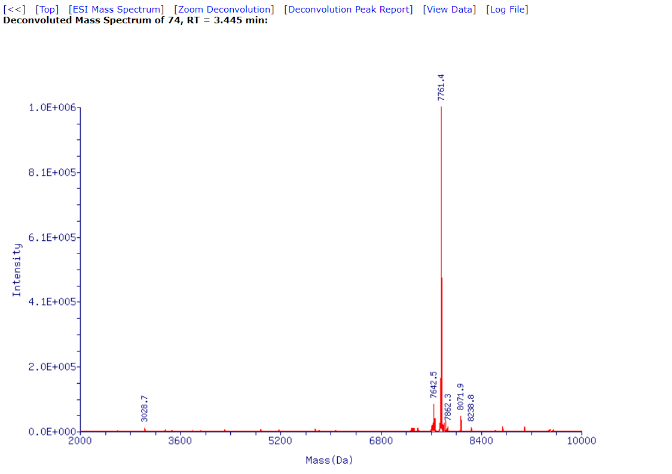


**ASO4/b2**


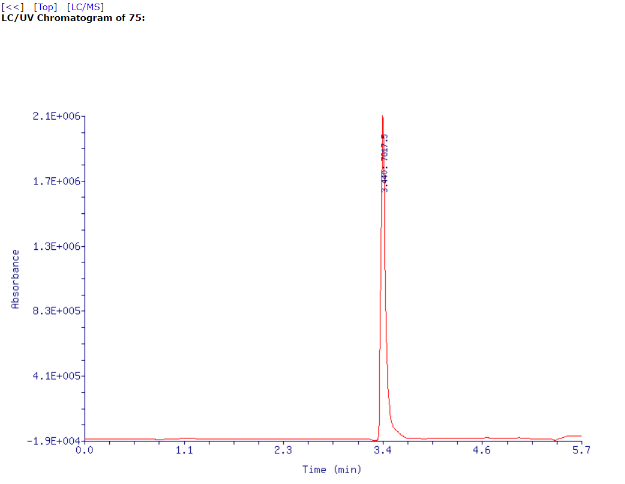

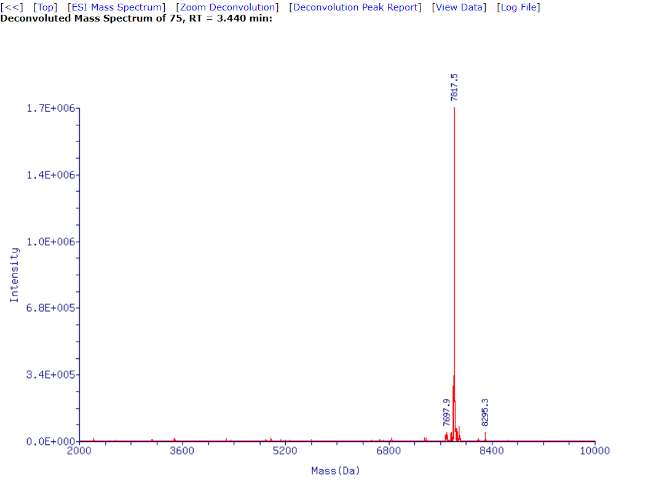


**ASO1/b2-sCy5**


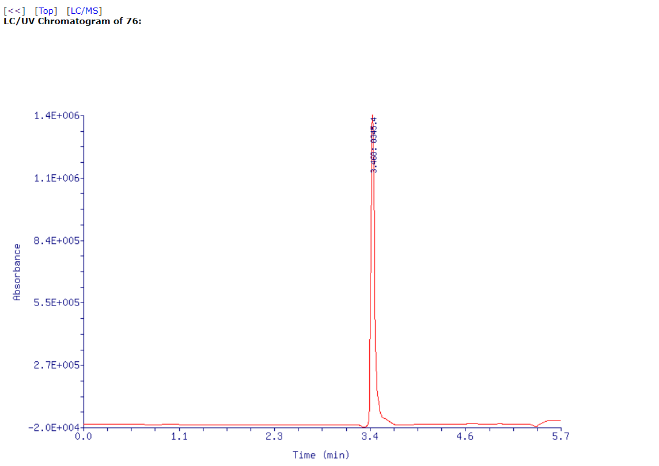

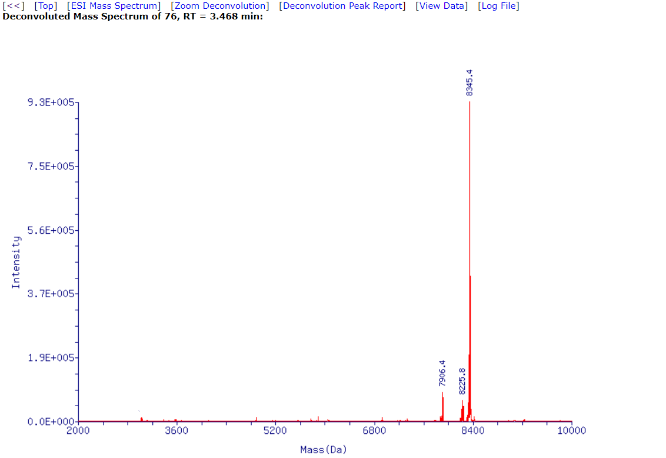


**ASO2/b2-sCy5**


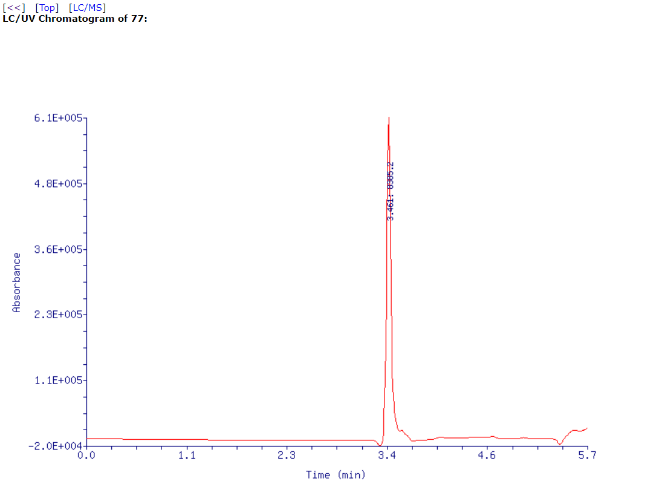

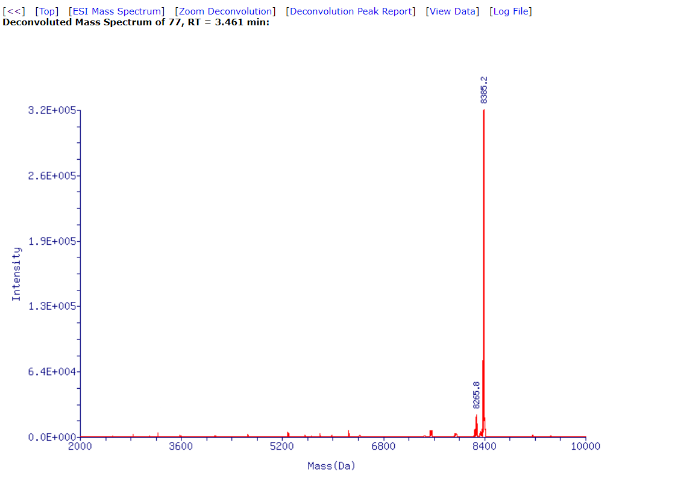


**ASO3/b2-sCy5**


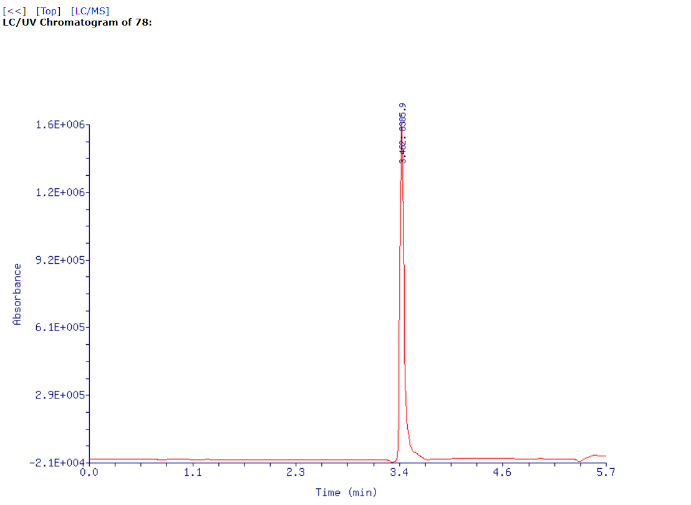

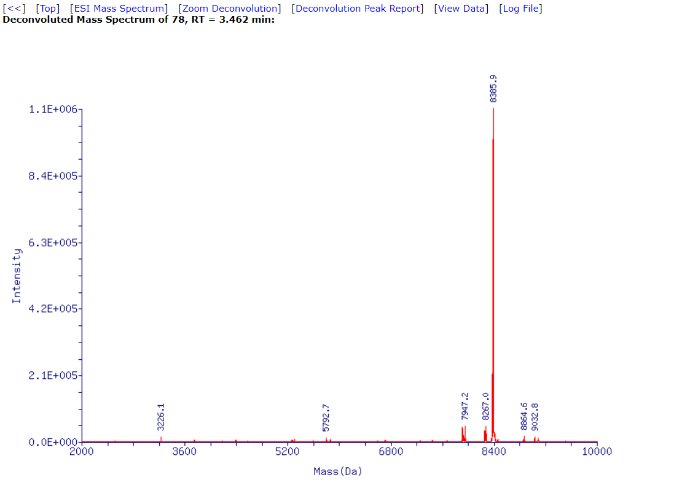


**ASO4/b2-sCy5**


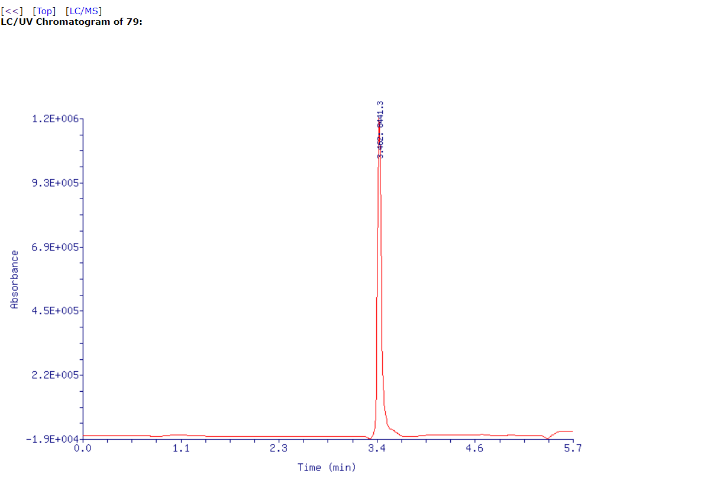

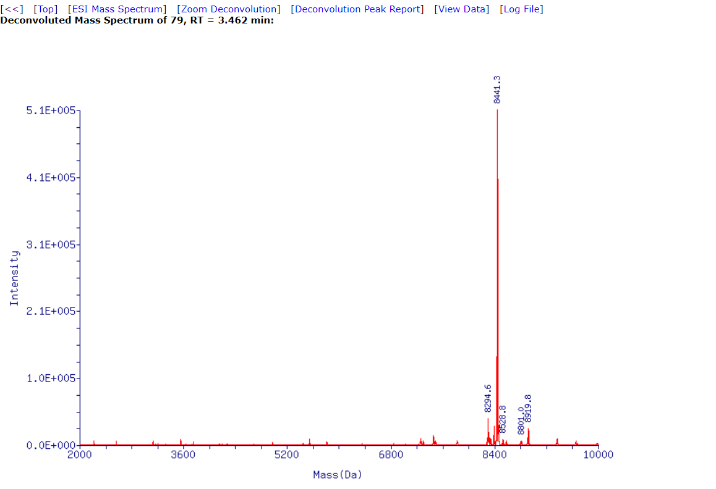


**scr-ASO1/b2**


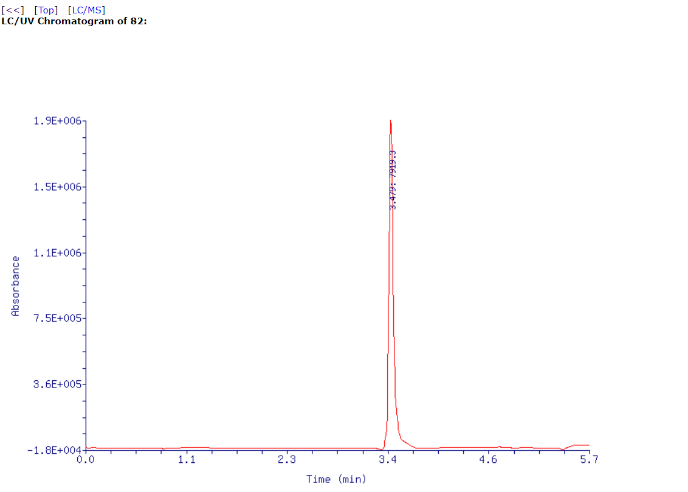

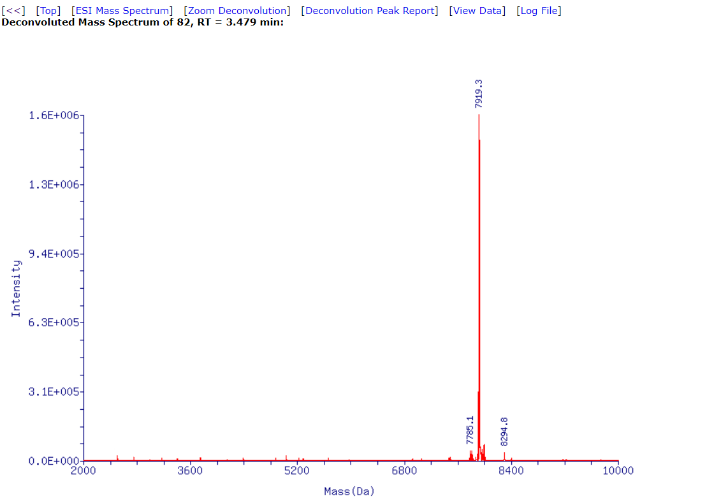


**scr-ASO1/b2-sCy5**


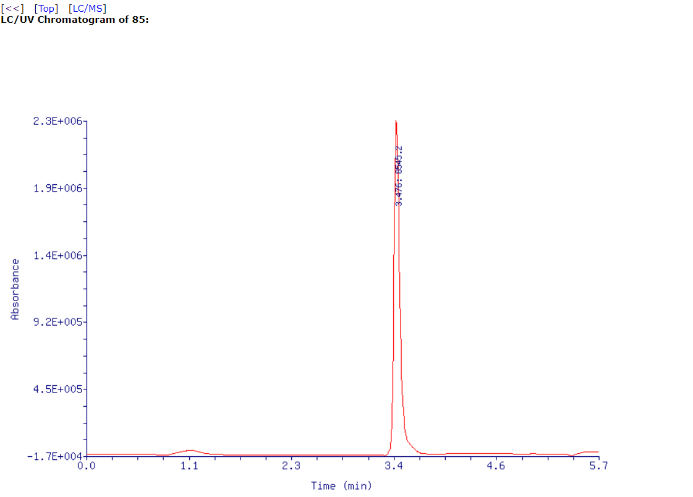

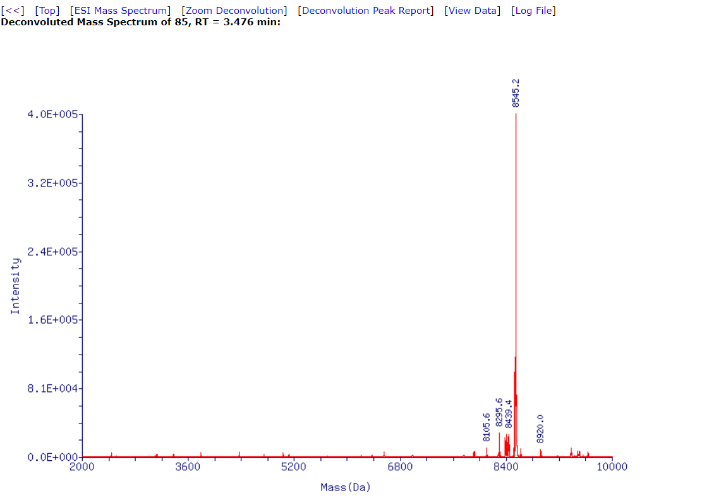


**ASO1/b3**


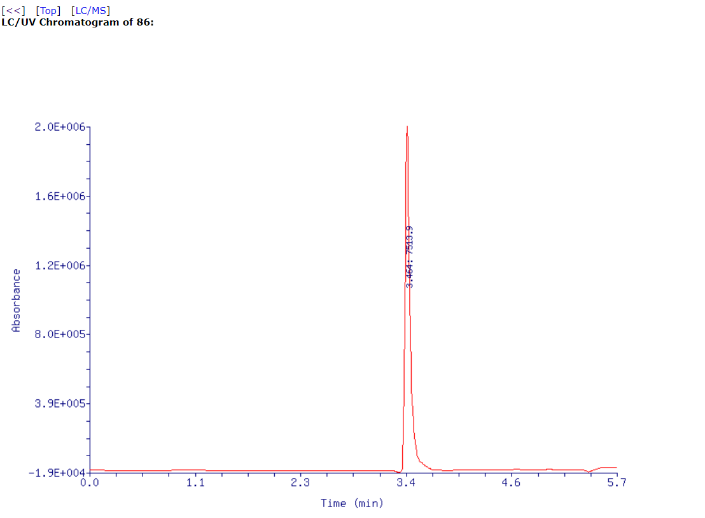

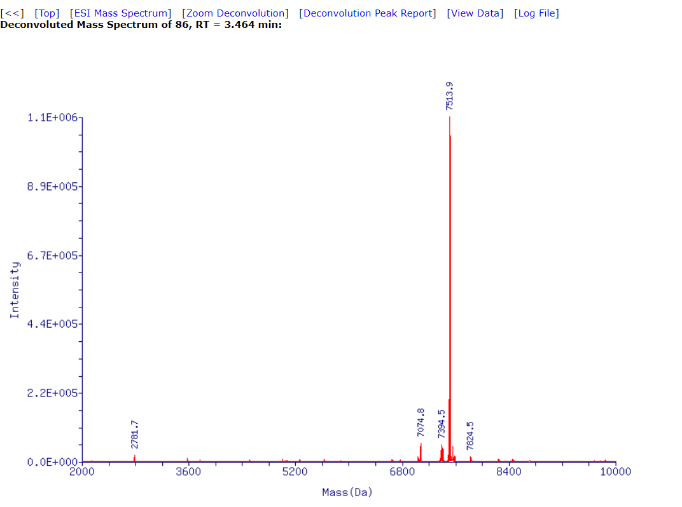


**ASO2/b3**


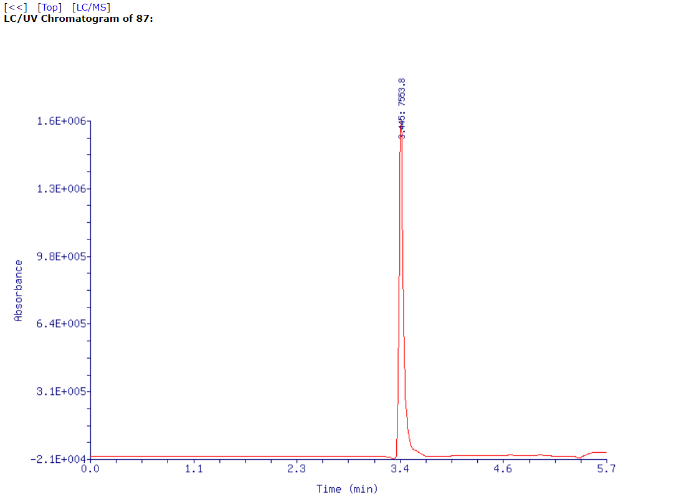

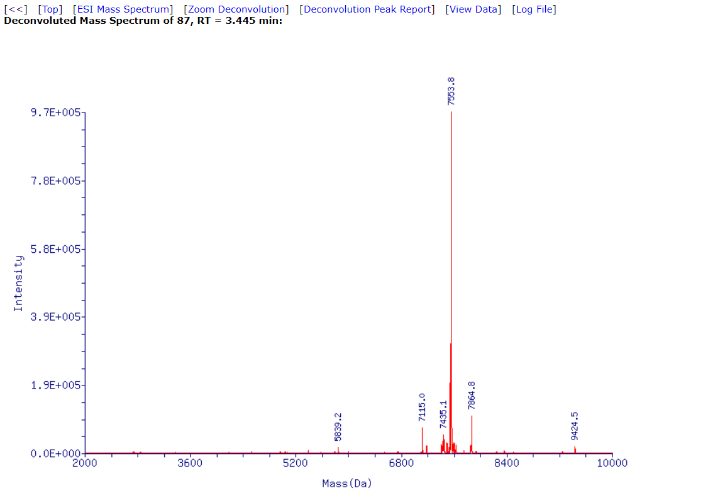


**ASO3/b3**


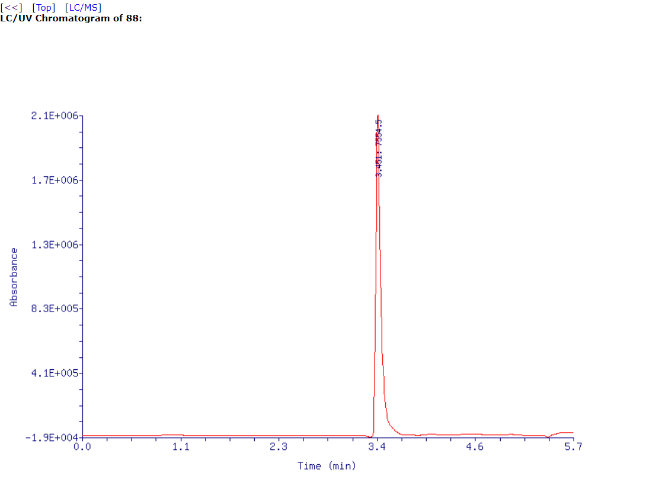

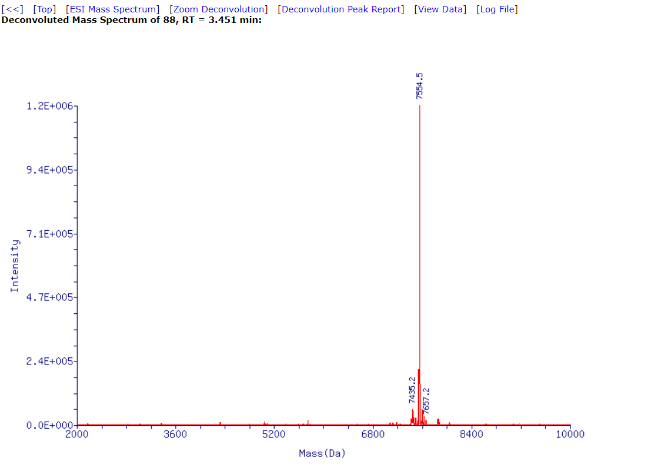


**ASO4/b3**


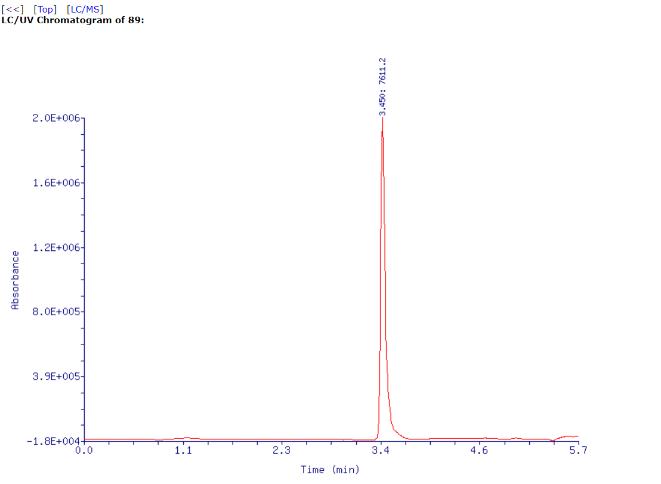

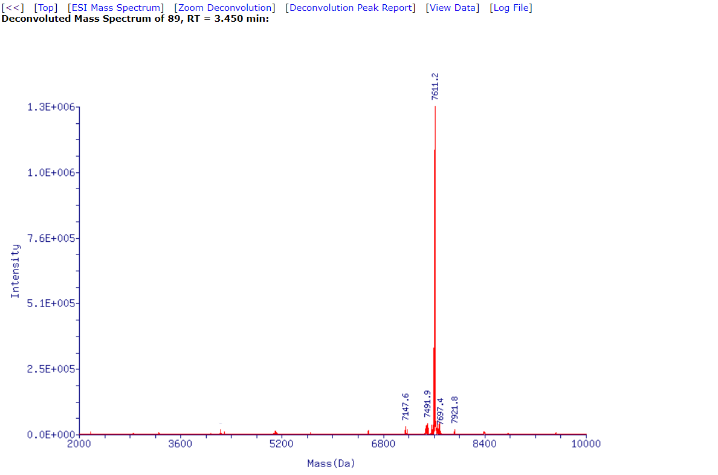


**ASO1/b3-sCy5**


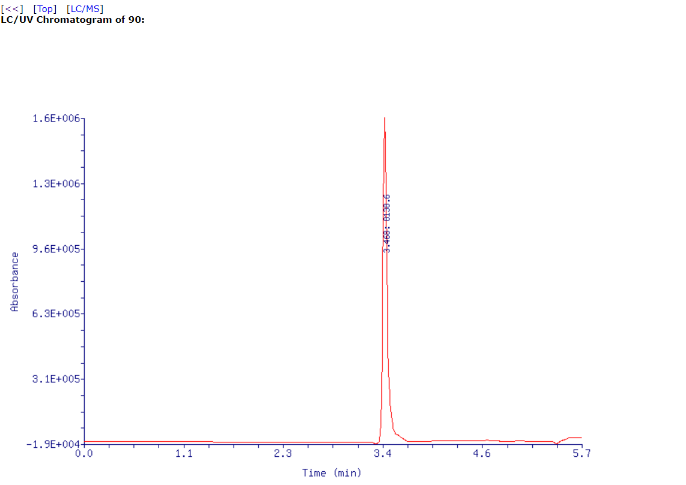

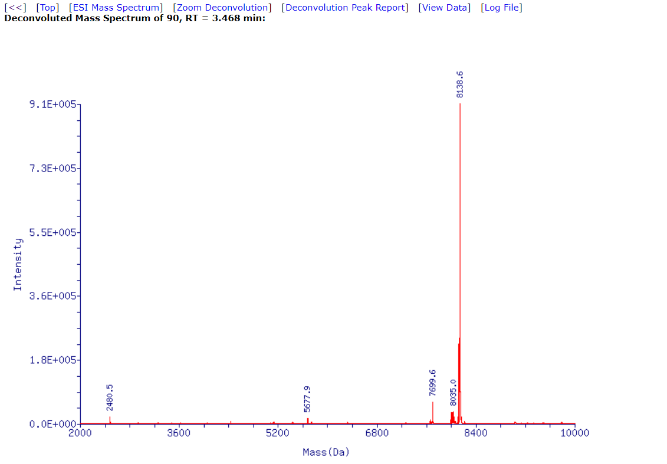


**ASO2/b3-sCy5**


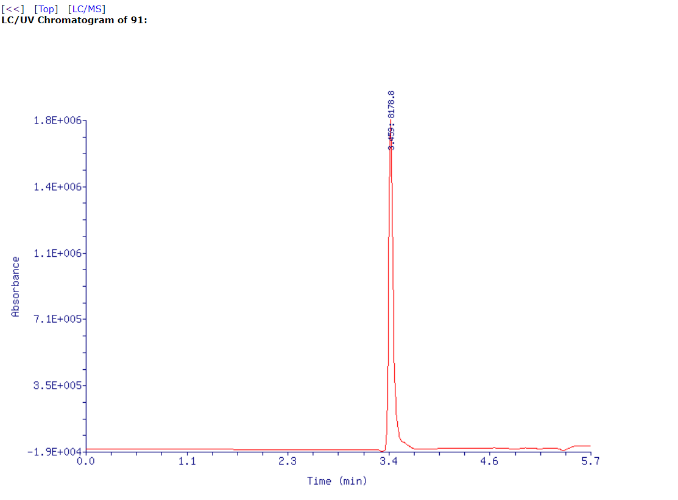

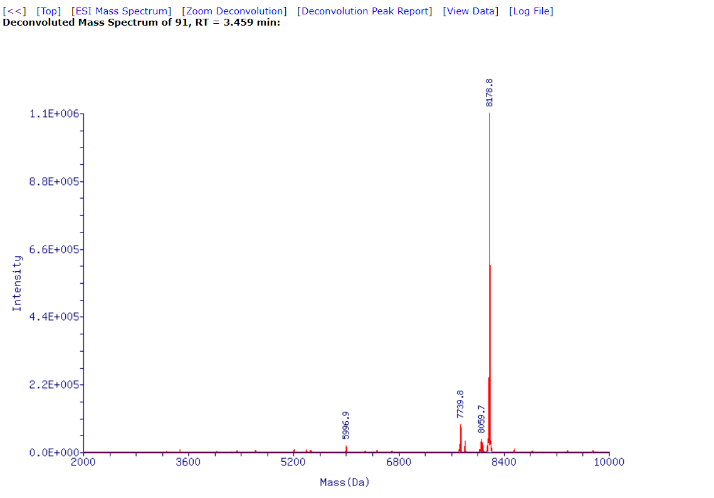


**ASO3/b3-sCy5**


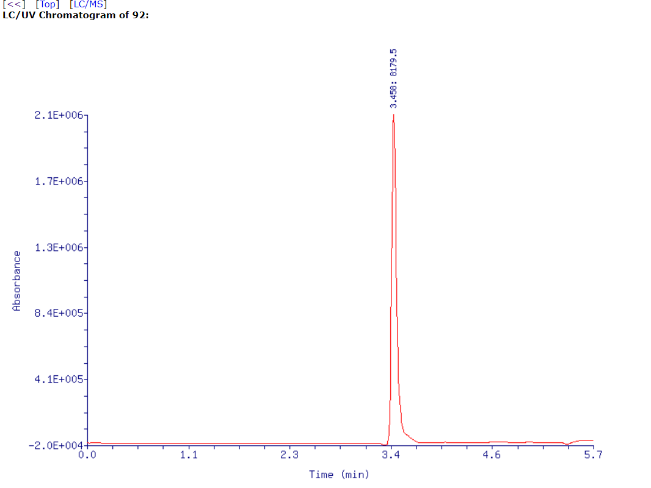

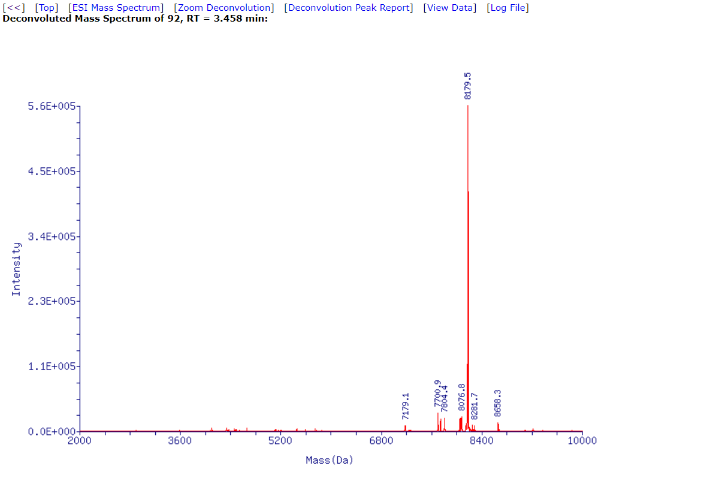


**ASO4/b3-sCy5**


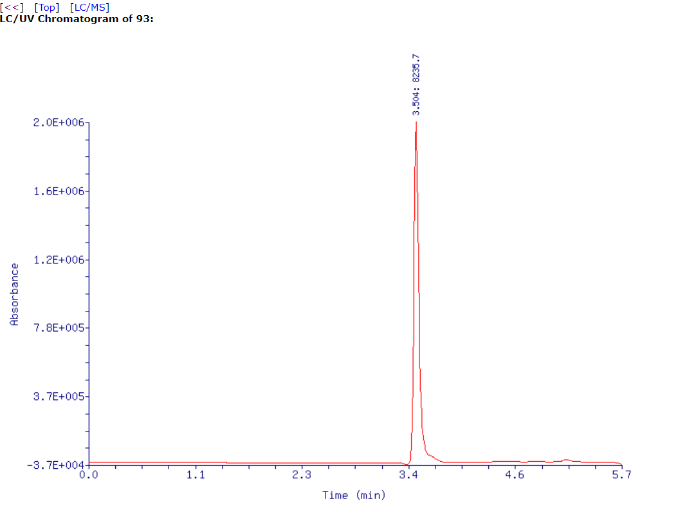

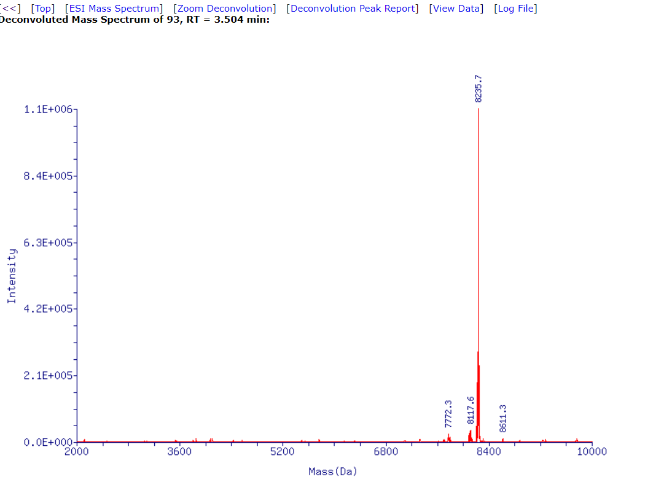


**scr-ASO1/b3**


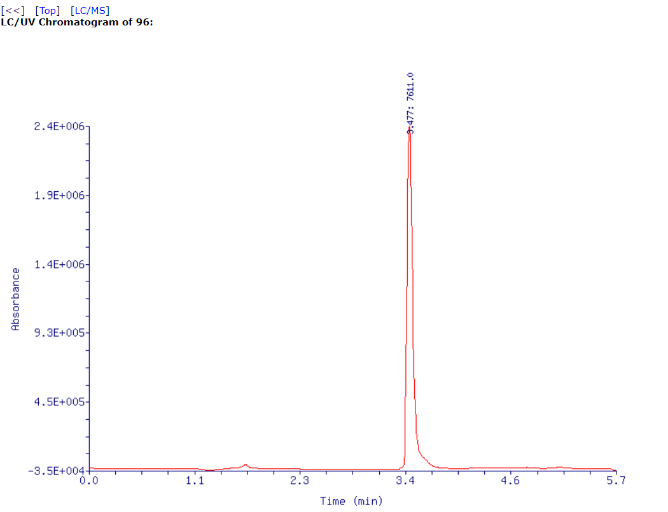

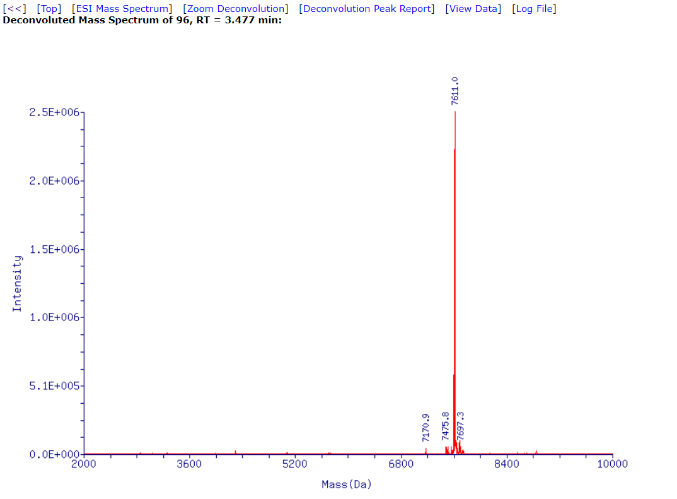


**scr-ASO1/b3-sCy5**


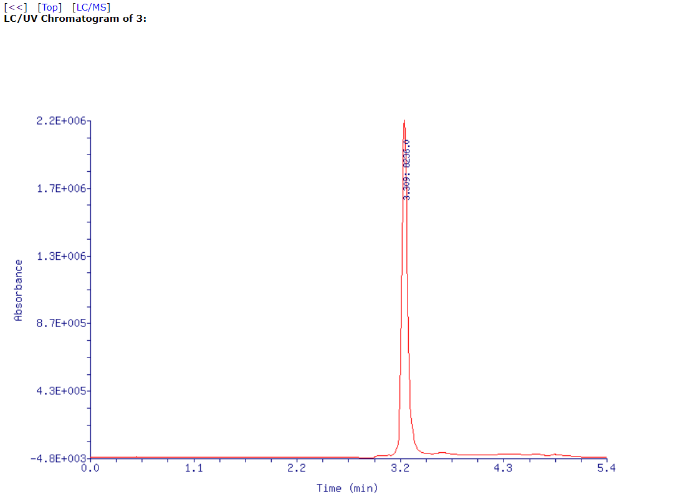

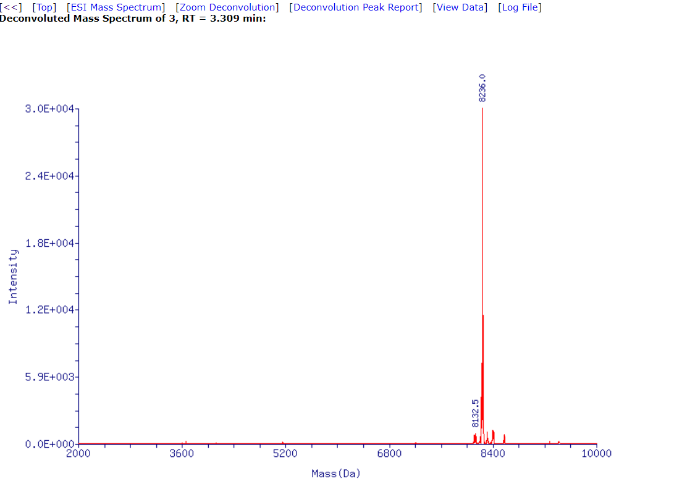


**ASO1/b4**


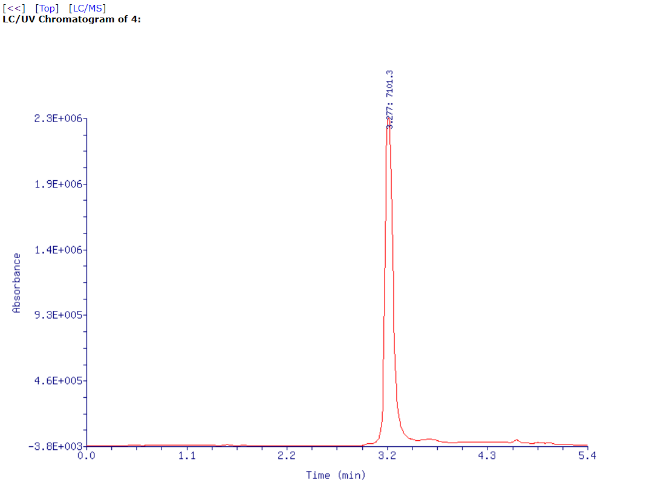

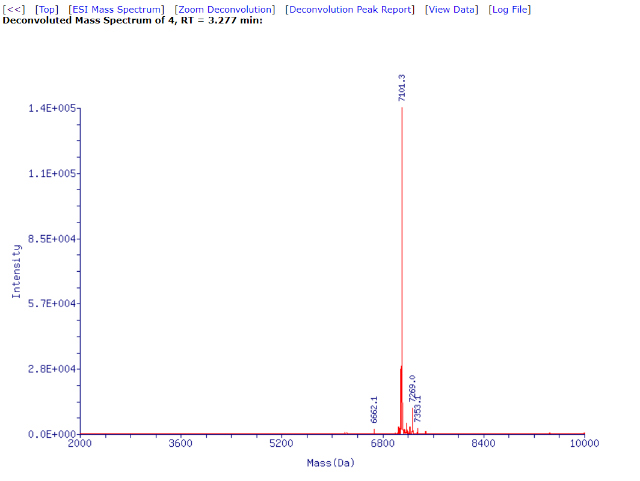


**ASO2/b4**


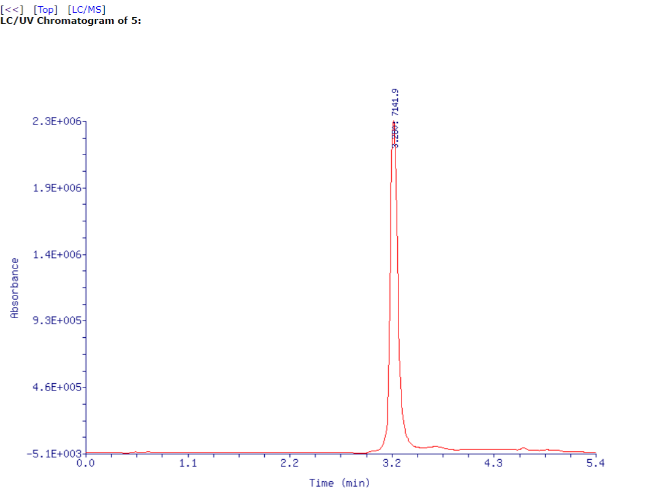

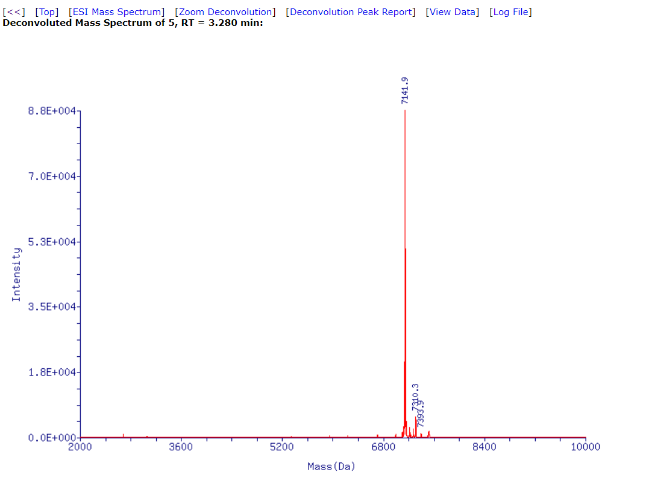


**ASO3/b4**


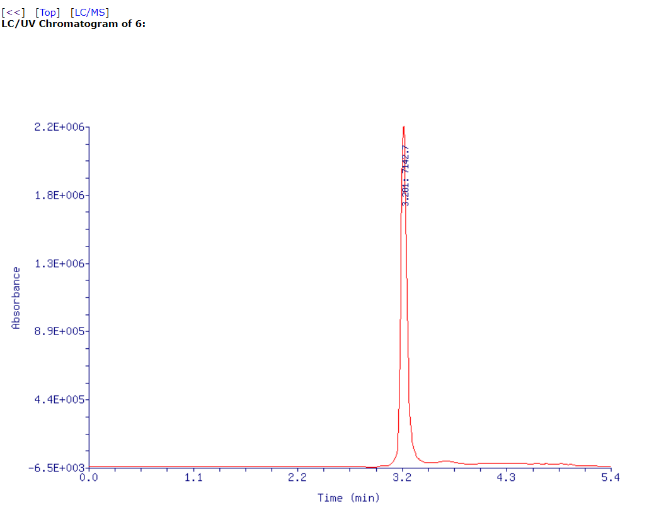

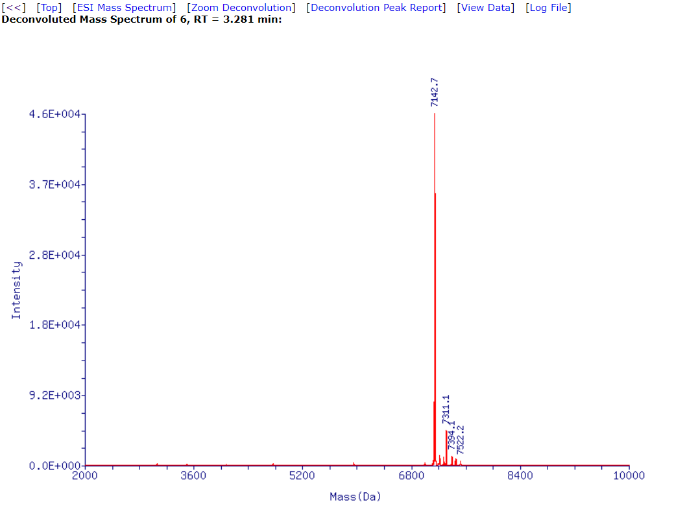


**ASO4/b4**


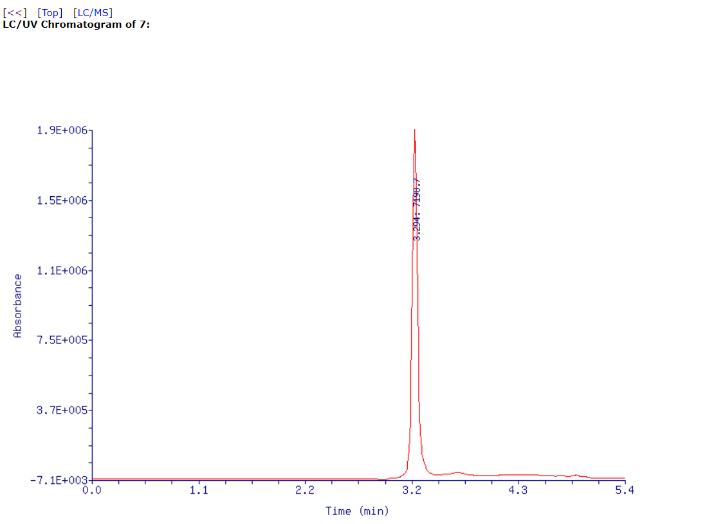

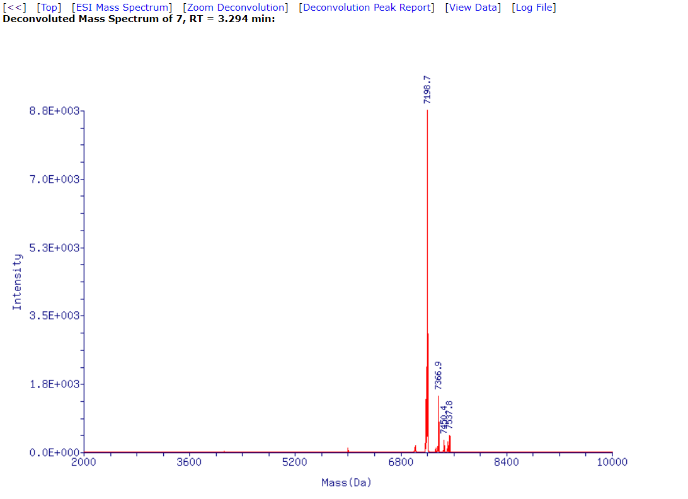


**ASO1/b4-sCy5**

**ASO2/b4-sCy5**

**ASO4/b4-sCy5**

**scr-ASO1/b4**

**scr-ASO1/b4-sCy5**

**ASO1**

**ASO2**

**ASO3**

**ASO4**

**scr-ASO1**

**ASO1_m**

**ASO2_m**

**ASO3_m**

**ASO4_m**

**ASO1_m-sCys**

**ASO2_m-sCys**

**ASO3_m-sCys**

**ASO4_m-sCys**

**scr-ASO1_m**

**scr-ASO1_m-sCy5**

**ASO1_md**

**ASO2_md**

**ASO3_md**

**ASO4_md**

**scr-ASO1_md**

**ASO2_md-MA257**

**ASO2_md-MA415**

**ASO4_md-MA257**

**scr-ASO1_md-MA415**

**scr-ASO1_md-MA257**

**ASO3_bmd-MA415**

**ASO3_bmd-MA257**

**Malat (ASO1)**

**Malat (ASO2)**

**Malat (ASO3)**

**Malat (ASO4)**

**Malat scr**
